# Microbial Transformation of *neo*-Clerodane Diterpenoid, Scutebarbatine F, by *Streptomyces* sp. CPCC 205437

**DOI:** 10.3389/fmicb.2021.662321

**Published:** 2021-04-14

**Authors:** Dewu Zhang, Xiaoyu Tao, Guowei Gu, Yujia Wang, Wenxia Zhao, Wuli Zhao, Yan Ren, Shengjun Dai, Liyan Yu

**Affiliations:** ^1^Institute of Medicinal Biotechnology, Chinese Academy of Medical Sciences and Peking Union Medical College, Beijing, China; ^2^School of Pharmacy, Binzhou Medical University, Yantai, China; ^3^School of Pharmacy, Yantai University, Yantai, China

**Keywords:** *neo*-clerodane diterpene, scutebarbatine F, biotransformation, cytotoxicity, antiviral activity, *Streptomyces*

## Abstract

Biotransformation of the *neo*-clerodane diterpene, scutebarbatine F (**1**), by *Streptomyces* sp. CPCC 205437 was investigated for the first time, which led to the isolation of nine new metabolites, scutebarbatine F_1_–F_9_ (**2**–**10**). Their structures were determined by extensive high-resolution electrospray ionization mass spectrometry (HRESIMS) and NMR data analyses. The reactions that occurred included hydroxylation, acetylation, and deacetylation. Compounds **2**–**4** and **8**–**10** possess 18-OAc fragment, which were the first examples of 13-spiro *neo*-clerodanes with 18-OAc group. Compounds **7**–**10** were the first report of 13-spiro *neo*-clerodanes with 2-OH. Compounds **1**–**10** were biologically evaluated for the cytotoxic, antiviral, and antibacterial activities. Compounds **5**, **7**, and **9** exhibited cytotoxic activities against H460 cancer cell line with inhibitory ratios of 46.0, 42.2, and 51.1%, respectively, at 0.3 μM. Compound **5** displayed a significant anti-influenza A virus activity with inhibitory ratio of 54.8% at 20 μM, close to the positive control, ribavirin.

## Introduction

Biotransformation of natural products has been proved to be an efficient and environmentally friendly approach to the preparation of new compounds, and biotransformation is a powerful tool for the regioselective and stereoselective introduction under mild conditions (Bianchini et al., 2015; Cao et al., 2015; Hegazy et al., 2015; Zafar et al., 2016; Khojasteh et al., 2019). Microbial biotransformation can be an attractive alternative to introduce hydroxyl and acetyl groups at specific positions, which were difficult to achieve by conventional synthesis methods. So biotransformation method has been widely used for structural modification of terpenoids (Silva et al., 2013; Bhatti and Khera, 2014; Rico-Martínez et al., 2014; de Sousa et al., 2018; Luchnikova et al., 2020).

**FIGURE 1:**
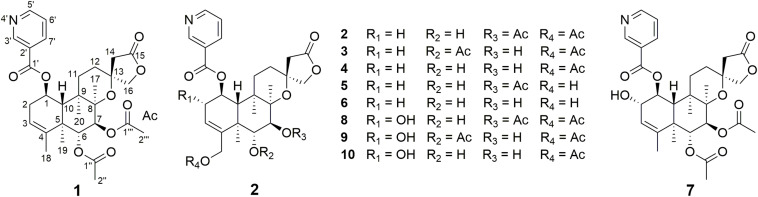
Metabolites **2**–**10** obtained by microbial transformation of **1** by *Streptomyces* sp.

Clerodane diterpenoids are a widespread class of natural products and widely distributed in medicinal herbs from various families, such as Lamiaceae, Euphorbiaceae, Compositae, and Salicaceae (Li et al., 2016). So far, over 1,300 clerodane diterpene derivatives have been reported, and they displayed a broad range of promising biological properties, such as cytotoxic, insect antifeedant, anti-inflammatory, antiprotozoal, and antibacterial activities (Li et al., 2016; Luan et al., 2019; Shen et al., 2021). Structurally, all of the natural clerodane diterpenes were classified as seven different types on the basis of the decalin ring and C-11–C-16 moiety (Supplementary Figure 1; Li et al., 2016). Although more than 1,300 clerodane diterpenoids have been reported, there are a few reports on biotransformation of *neo*-clerodane diterpenes (Hoffmann and Fraga, 1993; Choudhary et al., 2013; Mafezoli et al., 2014; Kumar et al., 2016; Vasconcelos et al., 2017). To the best of our knowledge, there is no report about biotransformation of 13-spiro *neo*-clerodane diterpenes till now. Scutebarbatine F (**1**) with 13-spiro *neo*-clerodane skeleton was firstly isolated from *Scutellaria barbata* in 2006 by Dai group and showed significant cytotoxic activities against HONE-1 nasopharyngeal, KB oral epidermoid carcinoma, and HT29 colorectal carcinoma cell lines (Dai et al., 2006). Subsequently, the chemical structure of scutebarbatine F was revised by Wang et al. (2010). Furthermore, the compound with the same chemical structure was also reported to be names as barbatine C and scutebata F (Nguyen et al., 2009; Zhu et al., 2010). In an attempt to obtain new biologically active *neo*-clerodane diterpenoids. We performed the biotransformation of scutebarbatine F using the *Streptomyces* sp. CPCC 205437, which resulted in the formation of nine previously undescribed metabolites (**2**–**10**) (Figure 1). Biotransformation of **1** introduces hydroxyl groups at C-2 and C-18, followed by introducing acetyl moiety in 18-OH. Herein, we describe the isolation, structure elucidation, and biological activities of nine biotransformation products (**2**–**10**).

## Materials and Methods

### General Experimental Procedures

Optical rotations were measured on an Autopol IV automatic polarimeter. UV spectra were recorded on a Persee TU-1901 UV-vis spectrometer. IR spectra were acquired on a PerkinElmer FT-IR/NIR spectrometer. The CD spectra were obtained on a JASCO J-815 spectropolarimeter. 1D and 2D NMR spectra were measured on Bruker ARX-600 spectrometer. Chemical shifts (δ) are given in ppm, and coupling constants (*J*) are given in hertz (Hz). Electrospray ionization mass spectrometry (ESIMS) data were taken on a Thermo LTQ mass spectrometer. High-resolution ESIMS (HRESIMS) was carried out using a Thermo LTQ Orbitrap XL mass spectrometer. Semi-preparative high-performance liquid chromatography (HPLC) was performed using NP7000 module (Hanbon Co. Ltd., China) equipped with a Shodex RI102 detector, and YMC ODS-A C_18_ column (250 mm × 10 mm, i.d., 5 μm) or SunFire C_18_ column (250 × 10 mm, i.d., 5 μm). Column chromatography (CC) was performed with silica gel (200–300 mesh, Qingdao Marine Chemical Inc., Qingdao, PR China) and Sephadex LH-20 (GE Healthcare). Analytical thin-layer chromatography (TLC) was carried out using pre-coated silica gel GF_254_ plates (Yan Tai Zi Fu Chemical Group Co., Yantai, China).

### Substrate

Scutebarbatine F was isolated and purified from *Scutellaria barbata* by the Dai group. Its structure was elucidated by HRESIMS and 1D and 2D NMR analyses as previously reported (Dai et al., 2006), and the purity was > 95% by HPLC analysis. The substrate was dissolved in dimethylformamide (DMF) before being used for biotransformation.

### Microorganism and Culture Medium

The strain was originally purchased from the BeNa Culture Collection (BNCC) and identified on the basis of 16s rRNA gene sequence analysis by China Pharmaceutical Culture Collection. The 16S rRNA sequence was strongly homologous to *Streptomyces kronopolitis* NEAU-ML8(T) (99.3%) and has been deposited in GenBank with accession number MW699542. The strain was deposited at the China Pharmaceutical Culture Collection (No. CPCC 205437). It was grown on potato dextrose agar (PDA) slant at 28°C and stored at 4°C. The medium of *Streptomyces* sp. was prepared on modified potato dextrose broth (PDB) medium (potato 200 g, glucose 20 g, KH_2_PO_4_ 3 g, MgSO_4_ 0.73 g, vitamin B_1_ 10 mg, distilled water 1 L).

### Fermentation and Biotransformation

The medium was distributed in 24 Erlenmeyer flasks of 1,000 ml containing 200 ml of modified PDB liquid medium and autoclaved at 121°C. The mycelial agar plugs were transferred into the flasks and incubated at 28°C for 3 days. Compound **1** (240 mg) was dissolved in DMF, added to each, flask and incubated at 28°C for an additional 6 days.

### Extraction and Isolation

After 6 days of incubation, the cultures were filtered under reduced pressure to obtain the filtrate and mycelia. The mycelia were extracted with methanol three times, and organic solvent was evaporated under reduced pressure to yield a crude extract. The CH_3_OH extract was suspended in water and further extracted with EtOAc to obtain EtOAc residue. The filtrate was extracted with EtOAc to afford EtOAc residue. The above two parts of EtOAc residues (2.4 g) were combined and firstly subjected to Sephadex LH-20 CC with CHCl_3_–CH_3_OH (1:1) to give five fractions (Fr.1–Fr.5). Fraction Fr.3 (1.0 g) was separated by silica gel CC using CHCl_3_-acetone gradient (100:0–0:100) to provide 14 fractions (Fr.3.1–Fr.3.14) on the basis of TLC analysis. Fraction Fr.3.7 (105 mg) was separated by reversed-phase semi-preparative HPLC eluting with CH_3_CN–H_2_O (45:55) at 5 ml/min to yield **2** (40 mg, 16.7%). Fraction Fr.3.8 (60 mg) was isolated by reversed-phase semi-preparative HPLC eluting with CH_3_CN–H_2_O (45:55) at 5 ml/min to give **3** (16 mg, 6.7%). Fraction Fr.3.9 (187 mg) was purified via reversed-phase semi-preparative HPLC eluting with CH_3_CN–H_2_O (45:55) at 5 ml/min to obtain **4** (26 mg, 10.8%). Fraction Fr.3.10 (40.5 mg) was applied to reversed-phase semi-preparative HPLC eluting with CH_3_CN–H_2_O (45:55) at 5 ml/min to afford four fractions (Fr.3.10.1-Fr.3.10.4); fraction Fr.3.10.2 (22 mg) was further purified by reversed-phase semi-preparative HPLC eluting with CH_3_CN–H_2_O (40:60) at 5 ml/min to yield **8** (1.8 mg, 0.8%), **9** (2.1 mg, 0.9%), and **10** (4.3 mg, 1.8%); fraction Fr.3.10.3 (15 mg) was further isolated via reversed-phase semi-preparative HPLC eluting with CH_3_CN–H_2_O (35:65) at 5 ml/min to give **5** (9.2 mg, 3.8%) and **7** (6.5 mg, 2.7%). Fraction Fr.3.11 (43 mg) was separated by reversed-phase semi-preparative HPLC eluting with CH_3_CN–H_2_O (30:70) at 5 ml/min to yield **6** (8.2 mg, 3.4%).

Scutebarbatine F_1_ (**2**): white amorphous powder; [α]^25^_*D*_ -37.7° (*c* 0.05, MeOH); IR (ν_*max*_): 3,475, 2,983, 1,782, 1,717, 1,374, 1,241, 1,110, 1,023, and 743 cm^–1^; CD (MeOH) Δε (nm): -2.34 (237.5); ESIMS *m*/*z* 572.3 [M + H]^+^; HRESIMS *m*/*z* 572.2498 [M + H]^+^ (calcd for C_30_H_38_NO_10_, 572.2496); ^1^H and ^13^C NMR data (see Tables 1, 2).

**TABLE 1:** ^1^H NMR (600 MHz, *J* in Hz) data of **2**–**6** in DMSO-*d*_6_.

No.	2	3	4	5	6
1	5.68, dt (9.6, 6.0)	5.64, dt (9.6, 6.0)	5.65, dt (9.6, 6.0)	5.68, dt (9.6, 6.0)	5.66, dt (9.6, 6.0)
2	2.71, m 2.29, m	2.71, m 2.33, m	2.67, m 2.27, m	2.71, m 2.27, m	2.68, m 2.24, m
3	5.56, m	5.68, m	5.52, m	5.64, m	5.60, m
6	3.90, d (10.2)	5.12, d (9.6)	3.63, d (9.6)	3.90, d (10.2)	3.63, d (9.6)
7	4.96, d (10.2)	3.52, d (9.6)	3.30, d (9.6)	4.97, d (10.2)	3.31, d (9.6)
10	2.65, d (9.6)	2.67, d (9.6)	2.58, d (9.6)	2.62, d (9.6)	2.54, d (9.6)
11	1.73, m 1.65, m	1.70, m 1.67, m	1.70, m 1.63, m	1.68, m 1.64, m	1.65, m 1.62, m
12	2.12, m 1.51, m	2.07, m 1.47, m	2.06, m 1.47, m	2.09, m 1.49, m	2.04, m 1.45, m
14	2.68, d (16.2) 2.66, d (16.2)	2.85, d (17.4) 2.69, d (17.4)	2.72, d (16.8) 2.63, d (16.8)	2.69, d (17.4) 2.65, d (17.4)	2.71, d (17.4) 2.61, d (17.4)
16	4.25, d (9.0) 4.16, d (9.0)	4.30, d (8.4) 4.11, d (8.4)	4.27, d (9.0) 4.13, d (9.0)	4.25, d (9.0) 4.15, d (9.0)	4.27, d (9.0) 4.12, d (9.0)
17	1.09, s	1.18, s	1.22, s	1.09, s	1.22, s
18	4.84, d (14.4) 4.69, dd (14.4, 1.8)	4.52, d (13.8) 4.40, d (13.8)	4.88, d (14.4) 4.69, d (14.4)	4.22, br d (15.0) 4.05, ddd (15.0, 4.2, 1.8)	4.22, br d (15.0) 4.03, ddd (15.0, 4.2, 1.8)
19	1.18, s	1.28, s	1.12, s	1.16, s	1.11, s
20	0.96, s	0.95, s	0.91, s	0.97, s	0.92, s
3’	9.08, d (2.4)	9.10, br s	9.07, d (2.4)	9.08, br s	9.07, d (2.4)
5’	8.84, br d (4.8)	8.84, d (4.8)	8.83, dd (4.8, 1.8)	8.84, d (4.8)	8.83, d (4.8)
6’	7.61, dd (7.8, 4.8)	7.60, dd (7.8, 4.8)	7.60, dd (7.8, 4.8)	7.60, dd (7.8, 4.8)	7.59, d (8.4, 4.8)
7’	8.28, ddd (7.8, 2.4, 1.8)	8.29, ddd (7.8, 2.4, 1.8)	8.27, ddd (7.8, 2.4, 1.8)	8.28, ddd (7.8, 1.8, 1.8)	8.26, ddd (7.8, 2.4, 1.8)
2”		2.02, s			
2”’	2.08, s				
2””	2.05, s	2.02, s	2.05, s		

**TABLE 2:** ^13^C NMR (150 MHz) data of **2**–**10** in DMSO-*d*_6_.

No.	2	3	4	5	6	7	8	9	10
1	71.4	71.3	71.7	71.9	72.2	75.1	75.4	74.9	75.6
2	32.2	31.9	32.1	32.1	32.0	71.5	71.7	71.3	71.7
3	119.0	122.6	118.2	117.3	116.9	126.8	123.4	126.0	122.8
4	143.3	141.4	143.9	148.7	149.3	141.4	142.5	140.4	143.1
5	44.3	43.2	43.9	44.3	43.8	45.5	46.3	45.3	46.0
6	68.9	75.3	71.9	69.1	72.2	72.4	69.2	75.5	72.2
7	75.6	72.4	73.7	75.6	73.7	73.2	75.4	72.0	73.5
8	80.3	81.1	80.8	80.3	80.7	80.6	80.6	81.5	81.2
9	38.2	37.9	37.9	38.2	37.9	37.9	37.8	37.5	37.5
10	42.5	42.9	42.5	42.6	42.6	41.1	41.3	41.4	41.2
11	28.3	28.5	28.6	28.2	28.6	28.1	28.3	28.6	28.6
12	27.3	27.9	27.8	27.3	27.8	27.5	27.2	27.9	27.7
13	76.5	76.2	76.2	76.5	76.2	76.5	76.5	76.3	76.2
14	43.4	43.2	43.4	43.4	43.4	43.1	43.2	43.1	43.2
15	174.4	174.2	174.4	174.3	174.4	174.0	174.4	174.3	174.5
16	76.2	75.8	76.2	76.2	76.2	76.0	76.3	76.0	76.4
17	19.5	19.5	19.8	19.6	19.9	18.4	18.9	19.0	19.2
18	64.1	63.7	64.2	61.7	62.0	19.4	63.4	62.5	63.6
19	15.9	16.8	15.8	16.1	16.1	17.2	16.8	17.6	16.7
20	20.8	20.7	21.0	21.0	21.1	20.6	20.8	20.8	20.9
1’	164.0	164.0	164.1	164.1	164.1	164.2	164.3	164.3	164.3
2’	125.6	125.6	125.6	125.7	125.7	125.9	125.9	126.3	126.0
3’	149.9	150.0	150.0	150.0	150.0	150.2	150.2	150.3	150.2
5’	153.8	153.8	153.8	153.9	153.8	153.8	153.8	153.9	153.8
6’	124.2	124.2	124.1	124.1	124.1	124.1	124.2	124.2	124.1
7’	137.0	137.1	137.0	137.0	136.9	137.1	137.1	137.1	137.1
1”		170.0				169.6		170.2	
2”		21.4				21.1		21.5	
1”’	170.7			170.6		170.3	170.7		
2”’	21.1			21.0		20.6	21.1		
1””	170.0	170.0	170.0				170.0	170.1	170.0
2””	20.9	20.8	20.9				20.9	20.7	20.9

Scutebarbatine F_2_ (**3**): white amorphous powder; [α]^25^_*D*_ -23.2° (*c* 0.04, MeOH); IR (ν_*max*_): 3,527, 2,990, 1,783, 1,717, 1,374, 1,241, 1,109, 1,023, and 743 cm^–1^; CD (MeOH) Δε (nm): -1.88 (237.5); ESIMS *m*/*z* 572.3 [M + H]^+^; HRESIMS *m*/*z* 572.2499 [M + H]^+^ (calcd for C_30_H_38_NO_10_, 572.2496); ^1^H and ^13^C NMR data (see Tables 1, 2).

Scutebarbatine F_3_ (**4**): white amorphous powder; [α]^25^_*D*_ -23.3° (*c* 0.06, MeOH); IR (ν_*max*_): 3,436, 2,983, 1,779, 1,716, 1,284, 1,241, 1,109, 1,022, and 743 cm^–1^; CD (MeOH) Δε (nm): -3.29 (239.5); ESIMS *m*/*z* 530.2 [M + H]^+^; HRESIMS *m*/*z* 530.2386 [M + H]^+^ (calcd for C_28_H_36_NO_9_, 530.2390); ^1^H and ^13^C NMR data (see Tables 1, 2).

Scutebarbatine F_4_ (**5**): white amorphous powder; [α]^25^_*D*_ -52.2° (*c* 0.05, MeOH); IR (ν_*max*_): 3,406, 1,780, 1,718, 1,286, 1,245, 1,112, 1,024, and 742 cm^–1^; CD (MeOH) Δε (nm): -2.91 (239); ESIMS *m*/*z* 530.3 [M + H]^+^; HRESIMS *m*/*z* 530.2382 [M + H]^+^ (calcd for C_28_H_36_NO_9_, 530.2390); ^1^H and ^13^C NMR data (see Tables 1, 2).

Scutebarbatine F_5_ (**6**): white amorphous powder; [α]^25^_*D*_ -20.8° (*c* 0.05, MeOH); IR (ν_*max*_): 3,387, 2,988, 1,780, 1,717, 1,286, 1,246, 1,112, 1,024, and 742 cm^–1^; CD (MeOH) Δε (nm): + 0.52 (218.5), -2.97 (240); ESIMS *m*/*z* 488.3 [M + H]^+^; HRESIMS *m*/*z* 488.2281 [M + H]^+^ (calcd for C_26_H_34_NO_8_, 488.2284); ^1^H and ^13^C NMR data (see Tables 1, 2).

Scutebarbatine F_6_ (**7**): white amorphous powder; [α]^25^_*D*_ + 33.3° (*c* 0.03, MeOH); IR (ν_*max*_): 3,406, 2,981, 1,780, 1,723, 1,287, 1,247, 1,203, 1,113, 1,024, and 741 cm^–1^; CD (MeOH) Δε (nm): -0.44 (264.5); ESIMS *m*/*z* 572.2 [M + H]^+^; HRESIMS *m*/*z* 572.2498 [M + H]^+^ (calcd for C_30_H_38_NO_10_, 572.2496); ^1^H and ^13^C NMR data (see Tables 2, 3).

**TABLE 3:** ^1^H NMR (600 MHz, *J* in Hz) data of **7**–**10** in DMSO-*d*_6_.

No.	7	8	9	10
1	5.63, dd (11.4, 7.8)	5.66, dd (11.4, 7.8)	5.60, dd (11.4, 7.8)	5.63, dd (11.4, 7.8)
2	4.28, m	4.34, dd (7.8, 2.4)	4.35, m	4.31, m
3	5.23, br s	5.44, d (2.4)	5.51, br s	5.40, dd (4.2, 1.8)
6	5.17, d (10.2)	3.72, d (10.2)	4.96, d (9.6)	3.46, dd (9.6, 3.6)
7	5.11, d (10.2)	4.91, d (10.2)	3.46, d (9.6)	3.25, dd (9.6, 7.8)
10	2.74, d (11.4)	2.65, d (11.4)	2.69, d (11.4)	2.58, d (11.4)
11	2.02, m 1.63, m	1.99, m 1.59, m	2.02, m 1.60, m	1.98, m 1.58, m
12	2.17, m 1.63, m	2.15, m 1.61, m	2.12, m 1.60, m	2.08, m 1.58, m
14	2.76, d (16.8) 2.76, d (16.8)	2.73, d (17.4) 2.64, d (17.4)	2.82, d (16.8) 2.73, d (16.8)	2.70, d (16.8) 2.66, d (16.8)
16	4.31, d (9.0) 4.07, d (9.0)	4.26, d (9.0) 4.12, d (9.0)	4.30, d (9.0) 4.07, d (9.0)	4.27, d (9.0) 4.09, d (9.0)
17	1.05, s	1.04, s	1.17, s	1.18, s
18	1.58, s	4.77, d (15.0) 4.70, br d (15.0)	4.57, d (13.8) 4.32, d (13.8)	4.79, br d (15.6) 4.72, br d (15.6)
19	1.38, s	1.29, s	1.38, s	1.24, s
20	0.95, s	0.91, s	0.89, s	0.86, s
3’	9.10, br s	9.12, br s	9.11, br s	9.08, d (1.8)
5’	8.86, br s	8.87, br s	8.86, br s	8.84, dd (5.4, 1.8)
6’	7.62, dd (7.8, 4.2)	7.63, dd (7.8, 4.2)	7.62, dd (7.8, 4.2)	7.61, dd (7.8, 5.4)
7’	8.28, ddd (7.8, 4.2, 1.8)	8.29, br d (7.8)	8.28, br d (7.8)	8.28, ddd (7.8, 1.8, 1.8)
2”	1.97, s		2.02, s	
2”’	2.01, s	2.07, s		
2””		2.07, s	2.04, s	2.06, s

Scutebarbatine F_7_ (**8**): white amorphous powder; [α]^25^_*D*_ + 66.0° (*c* 0.05, MeOH); IR (ν_*max*_): 3,355, 1,780, 1,726, 1,682, 1,240, 1,202, 1,111, 1,023, and 720 cm^–1^; CD (MeOH) Δε (nm): -0.39 (251.5); ESIMS *m*/*z* 588.2 [M + H]^+^; HRESIMS *m*/*z* 588.2441 [M + H]^+^ (calcd for C_30_H_38_NO_11_, 588.2445); ^1^H and ^13^C NMR data (see Tables 2, 3).

Scutebarbatine F_8_ (**9**): white amorphous powder; [α]^25^_*D*_ + 125.0° (*c* 0.06, MeOH); IR (ν_*max*_): 3,386, 1,781, 1,723, 1,682, 1,243, 1,203, 1,132, 1,025, and 721 cm^–1^; CD (MeOH) Δε (nm): -0.39 (248.5); ESIMS *m*/*z* 588.3 [M + H]^+^; HRESIMS *m*/*z* 588.2440 [M + H]^+^ (calcd for C_30_H_38_NO_11_, 588.2445); ^1^H and ^13^C NMR data (see Tables 2, 3).

Scutebarbatine F_9_ (**10**): white amorphous powder; [α]^25^_*D*_ + 32.6° (*c* 0.05, MeOH); IR (ν_*max*_): 3,364, 1,718, 1,684, 1,285, 1,203, 1,132, 1,024, 1,007, and 720 cm^–1^; ESIMS *m*/*z* 546.3 [M + H]^+^; HRESIMS *m*/*z* 546.2342 [M + H]^+^ (calcd for C_28_H_36_NO_10_, 546.2339); ^1^H and ^13^C NMR data (see Tables 2, 3).

### Cytotoxic Activity Assays

MTT assay was used to measure the cytotoxicity of compounds. Five human cancer cell lines (H460, HCT8, HT15, H1975, and MIA-PaCa-2) were obtained from American Type Culture Collection (ATCC). Cells (5 × 10^3^ cells/well) were added to 96-well culture dishes and grown for 24 h followed by the addition of fresh medium (100 ml) and the test compound. After an additional 48 h, the medium was removed, and fresh medium with MTT solution was added. The cells were incubated for 1 h, and then the optical density at 450 nm was determined. Compounds **1**–**10** were tested for H460 and HT15 cancer cell lines at six concentrations (10, 5, 2.5, 1.25, 0.625, and 0.3125 μM), and compounds **1**–**10** were tested for HCT8, H1975, and MIA-PaCa-2 cancer cell lines at three concentrations (100, 10, and 1 μM). Each concentration of the compounds was tested in three parallels. IC_50_ values for each cell line were determined with SigmaPlot software (Chen et al., 2019).

### Anti-influenza A Virus Activity Assays

All the metabolites were evaluated for their anti-influenza A virus (anti-IAV) (H1N1) activities as in previously described methods (Gao et al., 2014).

### Anti-HIV Activity Assays

All the metabolites were tested for their anti-HIV activities as in previously reported methods (Zhang et al., 2017).

### Antibacterial Activity Assays

All the metabolites were tested for their antibacterial activities as in previously described methods (Zhang et al., 2017).

## Results and Discussion

### Structure Elucidation

In this study, the incubation of scutebarbatine F (**1**, 240 mg) with *Streptomyces* sp. CPCC 205437 for 6 days led to the isolation of nine new metabolites (**2**–**10**). Their structures were elucidated by the HRESIMS and 1D and 2D NMR data.

**FIGURE 2:**
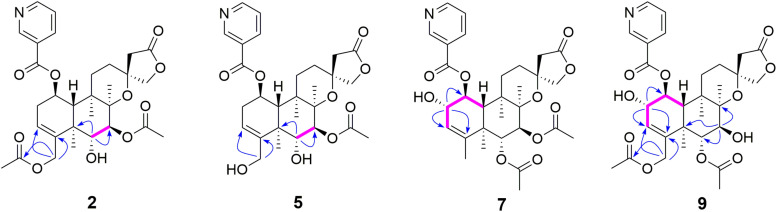
Key correlated spectroscopy (COSY) and heteronuclear multiple bond correlations (HMBCs) of **2**, **5**, **7**, and **9**.

**FIGURE 3:**
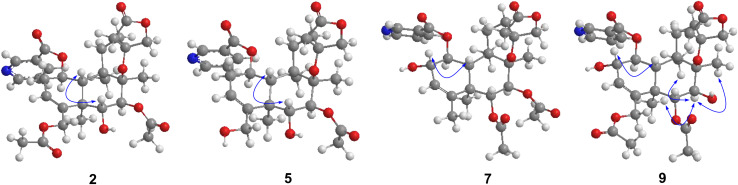
Key nuclear Overhauser enhancement spectroscopy (NOESY) correlations of **2**, **5**, **7**, and **9**.

Metabolite **2** was isolated as a white amorphous powder. The HRESIMS spectrum of **2** showed an ion peak at *m*/*z* 572.2498 [M + H]^+^ (calcd for C_30_H_38_NO_10_, 572.2496), 16 amu more massive than that of **1**. The ^1^H NMR spectrum of **2** displayed characteristic proton resonances of *neo*-clerodane diterpene skeleton including three tertiary methyls at δ_*H*_ 0.96 (3H, s, H-20), 1.09 (3H, s, H-17), and 1.18 (3H, s, H-19); two oxygenated methylenes at δ_*H*_ 4.16 (1H, d, *J* = 9.0 Hz, H_*a*_-16), 4.25 (1H, d, *J* = 9.0 Hz, H_*b*_-16), 4.69 (1H, dd, *J* = 14.4, 1.8 Hz, H_*a*_-18), and 4.84 (1H, d, *J* = 14.4 Hz, H_*b*_-18); three oxygenated methines at δ_*H*_ 3.90 (1H, d, *J* = 10.2 Hz, H-6), 4.96 (1H, d, *J* = 10.2 Hz, H-7), and 5.68 (1H, dt, *J* = 9.6, 6.0 Hz, H-1); and one olefinic proton at 5.56 (1H, m, H-3). The ^13^C NMR and distortionless enhancement by polarization transfer (DEPT) spectra of **2** showed 20 typical carbon signals for *neo*-clerodane diterpene skeleton, which consisted of three methyl carbons (δ_*C*_ 20.8, 19.5, and 15.9), six methylene carbons (δ_*C*_ 76.2, 64.1, 43.4, 32.2, 28.3, and 27.3, including two oxygenated), five methine carbons (δ_*C*_ 119.0, 75.6, 71.4, 68.9, and 42.5, including one olefinic and three oxygenated), and six quaternary carbons (δ_*C*_ 174.4, 143.3, 80.3, 76.5, 44.3, and 38.2, including one carbonyl, one olefinic, and two oxygenated). In addition, the ^1^H and ^13^C NMR spectra of **2** revealed the presence of two acetoxy groups [δ_*H*_ 2.08 (3H, s, H-2”’), δ_*C*_ 170.7 (C-1”’) and 21.1 (C-2”’); δ_*H*_ 2.05 (3H, s, H-2””), δ_*C*_ 170.0 (C-1””) and 20.9 (C-2””)] and a nicotinic acid ester unit [δ_*H*_ 9.08 (1H, d, *J* = 2.4 Hz, H-3’), 8.84 (1H, br d, *J* = 4.8 Hz, H-5’), 7.61 (1H, d, *J* = 7.8, 4.8 Hz, H-6’), and 8.28 (1H, ddd, *J* = 7.8, 2.4, 1.8 Hz, H-7’), δ_*C*_ 164.0 (C-1’), 125.6 (C-2’), 149.9 (C-3’), 153.8 (C-5’), 124.2 (C-6’), and 137.0 (C-7’)]. The general features of its ^1^H and ^13^C NMR spectra closely resembled those of substrate **1**, except that the methyl signal [δ_*H*_ 1.68 (H-18), δ_*C*_ 20.2 (C-18)] in **1** had disappeared, and a new oxygenated methylene signal [δ_*H*_ 4.84, 4.69, δ_*C*_ 64.1] in **2** was observed, suggesting the introduction of hydroxyl group at C-18. A detailed inspection of ^1^H and ^13^C NMR spectra of **2** showed high-field shifts for H-6 (δ_*H*_ 3.90) and C-6 (δ_*C*_ 68.9), in comparison with those of **1**. In the heteronuclear multiple bond correlation (HMBC) spectrum (Figure 2), the correlations from H-6 to C-4, C-5, C-7, and C-19, and from H-18 to C-3, C-4, C-5, and C-1””, indicates that acetoxy substituent at C-6 disappeared, and an acetoxy group was attached to C-18. Furthermore, the nuclear Overhauser enhancement spectroscopy (NOESY) correlation (Figure 3) of H-6/H-10, along with the large coupling constant of ^3^*J*_*H*–6,H–7_ (10.2 Hz), established the β-configuration of H-6. Therefore, metabolite **2** was determined as scutebarbatine F_1_.

Metabolite **3** was obtained as a white amorphous powder, and its molecular formula C_30_H_37_NO_10_ was established by HRESIMS at *m*/*z* 572.2499 [M + H]^+^, which is the same as that of **2**. The ^1^H NMR data of **3** showed oxygenated methine signals at δ_*H*_ 5.12 and 3.52, instead of the resonances at δ_*H*_ 3.90 and 4.96 in **2**. The HMBC cross-peaks from H-6 to C-4, C-5, C-7, C-19, and C-1”, and from H-7 to C-5, C-6, C-8, and C-17, assigned the acetoxy group at C-6, rather than C-7. The NOESY correlations of H-7/H-17 and H-19, together with the large coupling constant of ^3^*J*_*H*–6,H–7_ (9.6 Hz), assigned the α-configuration of H-7. Thus, metabolite **3** was elucidated as scutebarbatine F_2_.

Metabolite **4** was obtained as a white amorphous powder. HRESIMS gave an ion peak at *m*/*z* 530.2386 [M + H]^+^, which refers to the molecular formula C_28_H_35_NO_9_ (calcd for C_28_H_36_NO_9_, 530.2390), with 42 amu less than that of **3**. This suggested that **4** lacked an acetoxy moiety compared with **3**, which was further confirmed by the HMBCs from H-6 (δ_*H*_ 3.63) to C-4, C-5, C-7, and C-19. The NOESY correlations of H-6/H-10; H-7/H-17, H-19, and H-20, along with the large coupling constant of ^3^*J*_*H*–6,H–7_ (9.6 Hz), established the stereochemistry of **4** as the same as substrate **1**. Accordingly, metabolite **4** was determined to be scutebarbatine F_3_. Metabolite **6** was obtained as a white amorphous powder. HRESIMS data of **6** showed an [M + H]^+^ ion at *m*/*z* 488.2281, consistent with the molecular formula C_26_H_33_NO_8_, which is 42 amu less than that of **4**, indicating the loss of an acetoxy moiety in **4**. The HMBCs from H-6 to C-4, C-5, C-7, and C-19; from H-7 to C-5, C-6, C-8, and C-17; and from H-18 to C-3, C-4, and C-5 established the molecular structure of **6**. The enhancement of H-10 was observed when H-6 was irradiated, and irradiation of H-7 enhanced H-17, H-19, and H-20 in the NOE experiments, indicating the relative configuration of **6**. Thus, metabolite **6** was elucidated as scutebarbatine F_5_.

Metabolite **5** was obtained as a white amorphous powder and possessed the same molecular formula C_28_H_35_NO_9_ with **4** as supported by the HRESIMS ion peak at *m*/*z* 530.2382 [M + H]^+^ (calcd for C_28_H_36_NO_9_, 530.2390). The ^1^H and ^13^C NMR spectroscopic data of **5** resembled those of **2**, except for the absence of an acetoxy substituent. The HMBC cross-peaks (Figure 2) from H-18 to C-3, C-4, and C-5 suggest that the C-18 acetoxy group disappeared in **5** compared with **2**. In the NOE experiment, irradiation of H-6 resulted in the enhancement of H-10, together with the large coupling constant of ^3^*J*_*H*–6,H–7_ (10.2 Hz), suggesting that H-6 and H-10 were *syn*-oriented. Therefore, metabolite **5** was established as scutebarbatine F_4_.

Metabolite **7** was isolated as a white amorphous powder, and its molecular formula of C_30_H_38_NO_10_ was determined by the HRESIMS peak at *m*/*z* 572.2498 [M + H]^+^ (calcd for C_30_H_38_NO_10_, 572.2496), with 16 amu higher than that of **1**. The ^1^H and ^13^C NMR data of **7** were similar to those of **1**, except for the presence of oxygenated methine proton at δ_*H*_ 4.28 and oxygenated carbon at δ_*C*_ 71.5, suggesting that a hydroxyl group might be introduced. The location of introduced OH group was attached to C-2 by the ^1^H–^1^H correlated spectroscopy (COSY) correlations of H-1/H-2/H-3 and the HMBC cross-peaks (Figure 2) from H-2 to C-1, C-3, and C-4. The NOESY correlation (Figure 3) between H-2 and H-10, together with the large coupling constant of ^3^*J*_*H*–1,H–2_ (7.8 Hz), assigned the α-orientation of 2-OH. Therefore, metabolite **7** was determined to be scutebarbatine F_6_.

Metabolite **8** was obtained as a white amorphous powder. The HRESIMS spectrum displayed [M + H]^+^ ion peak at *m*/*z* 588.2441 (calcd for C_30_H_38_NO_11_, 588.2445), suggesting the substitution of one additional OH moiety compared with **2**. The ^1^H NMR data of **8** were similar to those of **2** except that the methylene signals of H-2 at δ_*H*_ 2.71 and 2.29 had disappeared, and an additional oxygen-bearing methine signal at δ_*H*_ 4.34 was observed, indicating that the OH group may be introduced at C-2. It was further confirmed by the ^1^H–^1^H COSY correlations of H-1/H-2/H-3 and the HMBCs from H-2 to C-1, C-3, and C-4. The stereochemistry of 2-OH was determined as α-orientation on the basis of the large coupling constant of ^3^*J*_*H*–1,H–2_ (7.8 Hz) and the NOE experiment, in which the integration value of H-10 when H-2 was irradiated. The integration value of H-10 was enhanced when H-6 was irradiated, indicating the α-configuration of 6-OH. Accordingly, metabolite **8** was elucidated to be scutebarbatine F_7_. Metabolite **9** was isolated as a white amorphous powder. The molecular formula C_30_H_37_NO_11_ was deduced from the HRESIMS peak at *m*/*z* 588.2440 [M + H]^+^, which is the same as that of **8**. Comparison of the ^1^H NMR data of **9** and **8** revealed an oxymethine signal at δ_*H*_ 3.46 in **9** in place of a signal at δ_*H*_ 3.72 in **8**, indicating that changes happened in the C-6 and C-7 positions, and the HMBC cross-peaks from H-6 to C-4, C-5, C-7, C-19, and C-1″ determined the acetoxy group at C-6. The NOESY correlations for H-2/H-10, H-7/H-19, and H-20 assigned the α-orientation of 2-OH and H-7. Thus, metabolite **9** was determined as scutebarbatine F_8_.

Metabolite **10** was isolated as a white amorphous powder. HRESIMS spectrum of **10** provided a major ion peak at *m*/*z* 546.2342 [M + H]^+^ (calcd for C_28_H_36_NO_10_, 546.2339), consistent with the molecular formula of C_28_H_35_NO_10_, suggesting that a hydroxyl group was introduced in comparison with **4**. The ^1^H and ^13^C NMR spectroscopic data of **10** were similar to those of **4**. The obvious difference was the presence of oxygenated methine signal at δ_*H*_ 4.31 instead of methylene signals at δ_*H*_ 2.67 and 2.27 in **4**. The OH moiety was attached to C-2 according to the ^1^H–^1^H COSY correlations of H-1/H-2/H-3 and the HMBC cross-peaks from H-2 to C-1, C-3, and C-4. The NOESY correlations of H-2/H-10, H-7/H-19, and H-20, together with the large coupling constants of ^3^*J*_*H*–1,H–2_ (7.8 Hz) and ^3^*J*_*H*–6,H–7_ (9.6 Hz), established the relative configuration of C-2, C-6, and C-7. Therefore, metabolite **10** was established as scutebarbatine F_9_.

### Biological Activity

**FIGURE 4:**
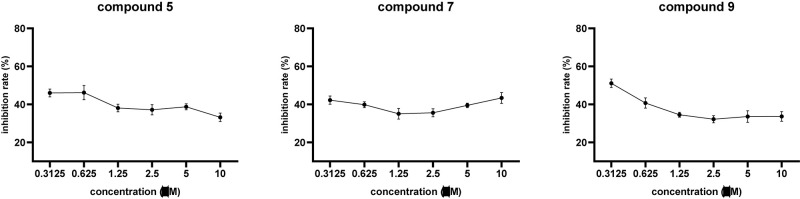
The inhibitory effects of compounds **5**, **7**, and **9** against H460 cancer cell.

Compounds **1**–**10** were evaluated for cytotoxic activities against five human cancer cell lines including H460 (large cell lung carcinoma), HCT8 (colon carcinoma), HT15 (colon carcinoma), H1975 (non-small cell lung carcinoma), and MIA-PaCa-2 (pancreatic carcinoma). Compounds **5**, **7**, and **9** showed cytotoxic activities against H460 cancer cell line with inhibitory ratios of 46.0, 42.2, and 51.1%, respectively, at 0.3 μM (Figure 4 and Supplementary Table 1). Compounds **1**–**10** were tested for antiviral activities; compound **5** displayed a significant anti-IAV (H1N1) activity with inhibitory ratio of 54.8% at 20 μM, which is close to the positive control, ribavirin (inhibitory ratio of 49.5% at 20 μM); and none of them shows anti-HIV activities at the concentration of 20 μM (Table 4). Compounds **1**–**10** were also evaluated for antibacterial activities against *Staphylococcus aureus* ATCC 29213 and *Escherichia coli* ATCC 25922. All compounds showed no antibacterial activities with the minimal inhibitory concentration (MIC) values > 32 μg/ml.

**TABLE 4:** Antiviral activities of **1**–**10**.

	Inhibition rate (%) in 20 μM	
	
Compound	IAV	HIV-1
**1**	14.16 ± 7.22	16.63 ± 1.57
**2**	0	12.85 ± 1.91
**3**	21.02 ± 11.73	0
**4**	20.09 ± 6.23	21.06 ± 0.87
**5**	54.75 ± 4.10	11.33 ± 2.39
**6**	0	0
**7**	0	0
**8**	24.57 ± 4.65	13.28 ± 1.13
**9**	26.51 ± 18.76	0
**10**	14.18 ± 6.76	7.96 ± 0.07
Ribavirin	49.46 ± 1.55	
Efavirenz		99.97 ± 0.25

**IAV, influenza A virus.**

## Conclusion

In conclusion, the biotransformation of 13-spiro *neo*-clerodane diterpene was investigated for the first time. Nine previously undescribed *neo*-clerodane diterpenoids **2**–**10** were afforded from microbial transformation of scutebarbatine F (**1**) by *Streptomyces* sp. CPCC 205437. Three types of reactions were involved in the biotransformation, including hydroxylation, acetylation, and deacetylation, in which the hydroxylation follows region- and stereo-selective routes. Hydroxylation was performed at C-2 and C-18; acetylation was further catalyzed at 18-OH; and deacetylation was carried out at C-6 and C-7. Compounds **2**–**4** and **8**–**10** were the first examples of 13-spiro *neo*-clerodanes with 18-OAc group. Compounds **7**–**10** were the first report of 13-spiro *neo*-clerodanes with 2-OH. Compounds **5**, **7**, and **9** displayed good cytotoxic activities against H460 cancer cell line at 0.3 μM. Compound **5** exhibited a significant anti-IAV activity. Our findings not only enrich the structural diversity of *neo*-clerodane family but also provide the guidance to biotransformation of *neo*-clerodane diterpenoids.

## Data Availability Statement

The original contributions presented in the study are included in the article/Supplementary Material, further inquiries can be directed to the corresponding author/s.

## Author Contributions

DZ and LY conceived and designed the experiments. DZ, XT, and GG contributed to fermentation, extraction, and isolation. DZ and SD contributed to structure elucidation and wrote the manuscript. YW, WeZ, and WuZ contributed to bioactivities tests. YR and LY were the project leaders guiding the experiments and manuscript writing. All authors contributed to the article and approved the submitted version.

## Conflict of Interest

The authors declare that the research was conducted in the absence of any commercial or financial relationships that could be construed as a potential conflict of interest.
