# Supplementary material for: Microbial Transformation of neo-Clerodane Diterpenoid, Scutebarbatine F, by Streptomyces sp. CPCC 205437
Source: Front Microbiol. 2021 Apr 14;12:662321. doi: 10.3389/fmicb.2021.662321 (PMC8079804; doi:10.3389/fmicb.2021.662321)

Supporting Information

Microbial Transformation of *neo*-Clerodane Diterpenoid, Scutebarbatine F by *Streptomyces* sp. CPCC 205437

Dewu Zhang[1], Xiaoyu Tao[1], Guowei Gu[1], Yujia Wang[1], Wenxia Zhao[1], Wuli Zhao[1], Yan Ren[2,*], Shengjun Dai[3,*] and Liyan Yu[1,*]

[1] Institute of Medicinal Biotechnology, Chinese Academy of Medical Sciences and Peking Union Medical College, Beijing, China, [2] School of Pharmacy, Binzhou Medical University, Yantai, China, [3] School of Pharmacy, Yantai University, Yantai, China

***Correspondence:**

Yan Ren

renyan198251@163.com

Shengjun Dai

daishengjun_9@hotmail.com

Liyan Yu

yly@cpcc.ac.cn

Table of Contents

Supplementary Figure S1. Basic skeletal classifications of clerodane diterpenes...S5

Supplementary Table S1. The cytotoxic activities against H460 cancer cell line of **1–10**……………………………………...…..S5

Supplementary Table S2. The cytotoxic activities against HT15 cancer cell line of **1–10**………………………………………..S6

Supplementary Table S3. The cytotoxic activities against HT15, H1975, and MIA-PaCa-2 cancer cell line of **1–10**…………………..S6

Supplementary Figure S2. ^{1}H NMR spectrum of **2**……………………………....S7

Supplementary Figure S3. ^{13}C NMR spectrum of **2**………………………….......S8

Supplementary Figure S4. DEPT spectrum of **2**………………………………….S9

Supplementary Figure S5. ^{1}H–^{1}H COSY spectrum of **2**……………………….S10

Supplementary Figure S6. HSQC spectrum of **2**……………………….........S11

Supplementary Figure S7. HMBC spectrum of **2**……………………………….S12

Supplementary Figure S8. NOESY spectrum of **2**…………………….………..S13

Supplementary Figure S9. HRESIMS spectrum of **2**…………..…..………..S14

Supplementary Figure S10. IR spectrum of **2**………………………………...S15

Supplementary Figure S11. UV spectrum of **2**………………………………….S15

Supplementary Figure S12. CD spectrum of **2**……………………..……...S16

Supplementary Figure S13. ^{1}H NMR spectrum of **3**…………………….…..S17

Supplementary Figure S14. ^{13}C NMR spectrum of **3**……………………...…S18

Supplementary Figure S15. DEPT spectrum of **3**………………………………S19

Supplementary Figure S16. ^{1}H–^{1}H COSY spectrum of **3**…………………...…S20

Supplementary Figure S17. HSQC spectrum of **3**……………………......…S21

Supplementary Figure S18. HMBC spectrum of **3**…………………………….…S22

Supplementary Figure S19. NOESY spectrum of **3**…………………….……...…S23

Supplementary Figure S20. HRESIMS spectrum of **3**…………….………..….S24

Supplementary Figure S21. IR spectrum of **3**…………………………….…S25

Supplementary Figure S22. UV spectrum of **3**…………………….....…..….S25

Supplementary Figure S23. CD spectrum of **3**…………………….....…..….S26

Supplementary Figure S24. ^{1}H NMR spectrum of **4**…………………….…..S27

Supplementary Figure S25. ^{13}C NMR spectrum of **4**……………………...…S28

Supplementary Figure S26. DEPT spectrum of **4**………………………………S29

Supplementary Figure S27. ^{1}H–^{1}H COSY spectrum of **4**…………………...…S30

Supplementary Figure S28. HSQC spectrum of **4**……………………......…S31

Supplementary Figure S29. HMBC spectrum of **4**…………………………….…S32

Supplementary Figure S30. 1D NOE spectrum of **4**……………………..….S33
Supplementary Figure S31. HRESIMS spectrum of **4**……………….……..….S34
Supplementary Figure S32. IR spectrum of **4**…………………….……..…..S35
Supplementary Figure S33. UV spectrum of **4**……………………...…..…..S35
Supplementary Figure S34. CD spectrum of **4**……………………...…..…..S36
Supplementary Figure S35. ^{1}H NMR spectrum of **5**……………………..…..S37
Supplementary Figure S36. ^{13}C NMR spectrum of **5**……………………...…S38
Supplementary Figure S37. DEPT spectrum of **5**……………………………S39
Supplementary Figure S38. ^{1}H–^{1}H COSY spectrum of **5**……………………...…S40
Supplementary Figure S39. HSQC spectrum of **5**…………………….......…S41
Supplementary Figure S40. HMBC spectrum of **5**……………………….…….S42
Supplementary Figure S41. 1D NOE spectrum of **5**……………………....…S43
Supplementary Figure S42. HRESIMS spectrum of **5**……………….……..….S44
Supplementary Figure S43. IR spectrum of **5**…………………….……..….S45
Supplementary Figure S44. UV spectrum of **5**……………………...…..…..S45
Supplementary Figure S45. CD spectrum of **5**……………………..…..…S46
Supplementary Figure S46. ^{1}H NMR spectrum of **6**…………………….….S47
Supplementary Figure S47. ^{13}C NMR spectrum of **6**……………………….S48
Supplementary Figure S48. DEPT spectrum of **6**…………………………..….S49
Supplementary Figure S49. ^{1}H–^{1}H COSY spectrum of **6**……………………..….S50
Supplementary Figure S50. HSQC spectrum of **6**…………………….........S51
Supplementary Figure S51. HMBC spectrum of **6**………………………….S52
Supplementary Figure S52. 1D NOE spectrum of **6**……………………….......S53
Supplementary Figure S53. HRESIMS spectrum of **6**……………….….……S54
Supplementary Figure S54. IR spectrum of **6**…………………….………..….S55
Supplementary Figure S55. UV spectrum of **6**……………………...…...….S55
Supplementary Figure S56. CD spectrum of **6**……………………..…….S56
Supplementary Figure S57. ^{1}H NMR spectrum of **7**…………………...…...S57
Supplementary Figure S58. ^{13}C NMR spectrum of **7**…………………….....S58
Supplementary Figure S59. DEPT spectrum of **7**…………………………..….S59
Supplementary Figure S60. ^{1}H–^{1}H COSY spectrum of **7**…………………......S60
Supplementary Figure S61. HSQC spectrum of **7**…………………….......….S61
Supplementary Figure S62. HMBC spectrum of **7**………………………….S62
Supplementary Figure S63. NOESY spectrum of **7**……………………...…S63
Supplementary Figure S64. HRESIMS spectrum of **7**……………….……..….S64
Supplementary Figure S65. IR spectrum of **7**…………………….……..….S65

Supplementary Figure S66. UV spectrum of **7**……………………...…...….S65
Supplementary Figure S67. CD spectrum of **7**………………….….…....….S66
Supplementary Figure S68. ^{1}H NMR spectrum of **8**……………………..…...S67
Supplementary Figure S69. ^{13}C NMR spectrum of **8**……………………...….S68
Supplementary Figure S70. DEPT spectrum of **8**…………………………….S69
Supplementary Figure S71. ^{1}H–^{1}H COSY spectrum of **8**………………....….S70
Supplementary Figure S72. HSQC spectrum of **8**……………………......….S71
Supplementary Figure S73. HMBC spectrum of **8**……………………….…….S72
Supplementary Figure S74. 1D NOE spectrum of **8**…………….……...….S73
Supplementary Figure S75. HRESIMS spectrum of **8**…………….……...….S74
Supplementary Figure S76. IR spectrum of **8**……………………..……....….S75
Supplementary Figure S77. UV spectrum of **8**……………………...…...….S75
Supplementary Figure S78. CD spectrum of **8**…………………….….…....….S76
Supplementary Figure S79. ^{1}H NMR spectrum of **9**……………………..…...S77
Supplementary Figure S80. ^{13}C NMR spectrum of **9**……………………...….S78
Supplementary Figure S81. DEPT spectrum of **9**…………………………….S79
Supplementary Figure S82. ^{1}H–^{1}H COSY spectrum of **9**………………....….S80
Supplementary Figure S83. HSQC spectrum of **9**……………………......….S81
Supplementary Figure S84. HMBC spectrum of **9**……………………….…….S82
Supplementary Figure S85. NOESY spectrum of **9**…………….……...….S83
Supplementary Figure S86. HRESIMS spectrum of **9**…………….………….S84
Supplementary Figure S87. IR spectrum of **9**…………………………….…….S85
Supplementary Figure S88. UV spectrum of **9**……………………...…...….S85
Supplementary Figure S89. CD spectrum of **9**……………………...…..….S86
Supplementary Figure S90. ^{1}H NMR spectrum of **10**……………….….......S87
Supplementary Figure S91. ^{13}C NMR spectrum of **10**…………….……...….S88
Supplementary Figure S92. DEPT spectrum of **10**………………………..….S89
Supplementary Figure S93. ^{1}H–^{1}H COSY spectrum of **10**…………..…....….S90
Supplementary Figure S94. HSQC spectrum of **10**……………………….…….S91
Supplementary Figure S95. HMBC spectrum of **10**………………….…...….S92
Supplementary Figure S96. NOESY spectrum of **10**…………………......….S93
Supplementary Figure S97. HRESIMS spectrum of **10**……………..……….S94
Supplementary Figure S98. IR spectrum of **10**…………………….…....….S95
Supplementary Figure S99. UV spectrum of **10**……….………….….…..….S95
Supplementary Figure S100. CD spectrum of **10**…………………....…...….S96

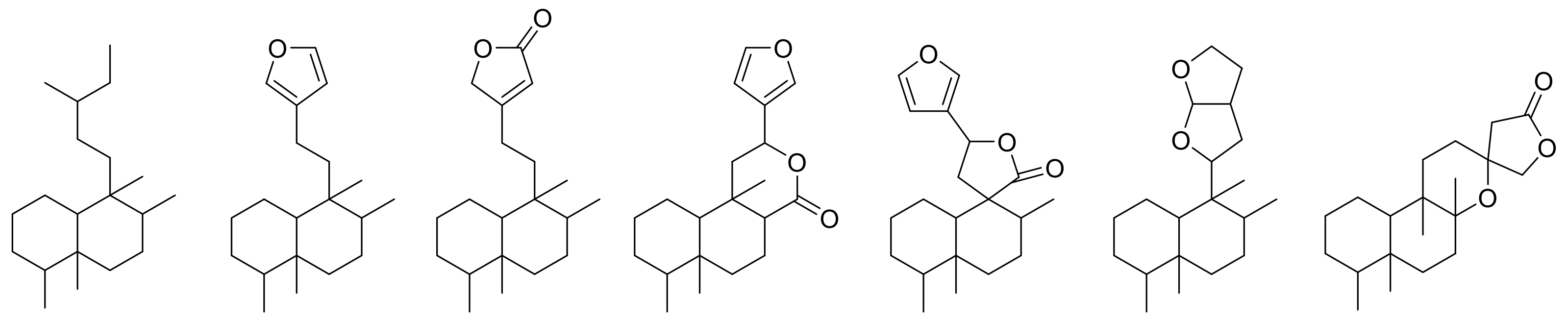

I II III IV V VI VII

Supplementary Figure S1. Basic skeletal classifications of clerodane diterpenes.

Supplementary Table S1. The cytotoxic activities against H460 cancer cell line of **1–10**.

	inhibition rate (%)					
no.	10 μM	5 μM	2.5 μM	1.25 μM	0.625 μM	0.3125 μM
**1**	<30	<30	<30	<30	<30	<30
**2**	<30	<30	<30	<30	<30	<30
**3**	<30	<30	<30	<30	<30	<30
**4**	<30	<30	<30	<30	<30	<30
**5**	33.17±2.23	38.74±1.60	37.16±2.67	38.10±1.98	46.19±3.76	46.04±2.06
**6**	<30	<30	<30	<30	<30	<30
**7**	43.40±2.87	39.46±1.22	35.58±2.10	35.04±2.83	39.81±1.53	42.20±2.17
**8**	<30	<30	<30	<30	<30	<30
**9**	33.65±2.55	33.61±3.02	32.19±1.89	34.52±1.31	40.69±2.69	51.09±2.21
**10**	<30	<30	<30	<30	<30	<30

Supplementary Table S2. The cytotoxic activities against HT15 cancer cell line of **1–10**.

no.	inhibition rate (%)					
	10 μM	5 μM	2.5 μM	1.25 μM	0.625 μM	0.3125 μM
**1**	<30	<30	<30	<30	<30	<30
**2**	<30	<30	<30	<30	<30	<30
**3**	<30	<30	<30	<30	<30	<30
**4**	<30	<30	<30	<30	<30	<30
**5**	<30	<30	<30	<30	<30	<30
**6**	<30	<30	<30	<30	<30	<30
**7**	<30	<30	<30	<30	<30	<30
**8**	<30	<30	<30	<30	<30	<30
**9**	<30	<30	<30	<30	<30	<30
**10**	<30	<30	<30	<30	<30	<30

Supplementary Table S3. The cytotoxic activities against HT15, H1975, and MIA-PaCa-2 cancer cell line of **1–10**.

no.	inhibition rate (%)								
	HCT8			H1975			MIA-PaCa-2		
	100 μM	10 μM	1 μM	100 μM	10 μM	1 μM	100 μM	10 μM	1 μM
**1**	<30	<30	<30	<30	<30	<30	<30	<30	<30
**2**	<30	<30	<30	<30	<30	<30	32.46±1.50	<30	<30
**3**	<30	<30	<30	<30	<30	<30	<30	<30	<30
**4**	<30	<30	<30	<30	<30	<30	<30	<30	<30
**5**	42.91±1.33	<30	<30	<30	<30	<30	<30	<30	<30
**6**	<30	<30	<30	<30	<30	<30	<30	<30	<30
**7**	<30	<30	<30	<30	<30	<30	<30	<30	<30
**8**	<30	<30	<30	<30	<30	<30	<30	<30	<30
**9**	52.95±2.10	32.77±1.46	<30	<30	<30	<30	<30	<30	<30
**10**	<30	<30	<30	<30	<30	<30	<30	<30	<30

Supplementary Figure S2. ^{1}H NMR spectrum of **2**

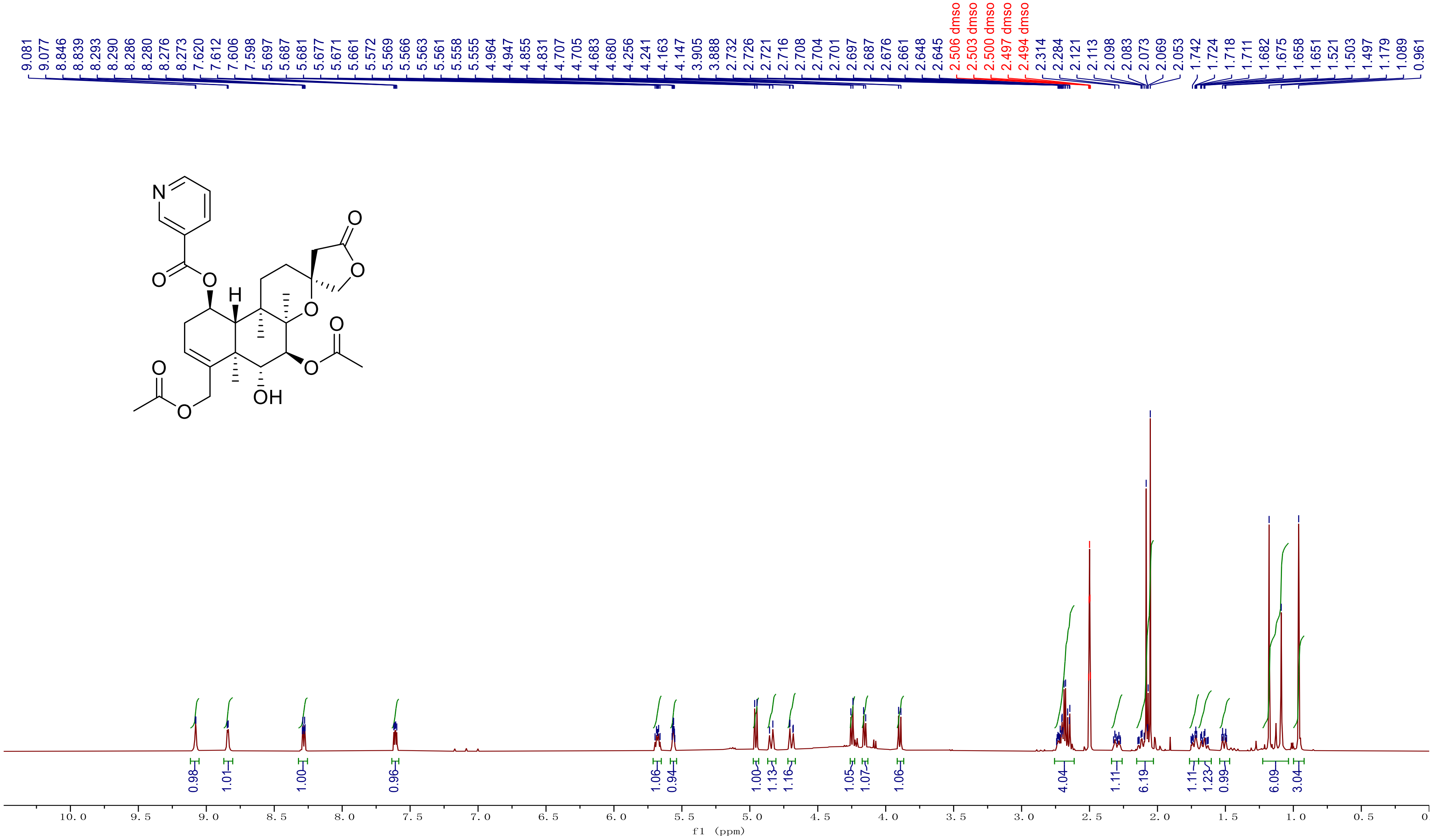

Supplementary Figure S3. ^{13}C NMR spectrum of **2**

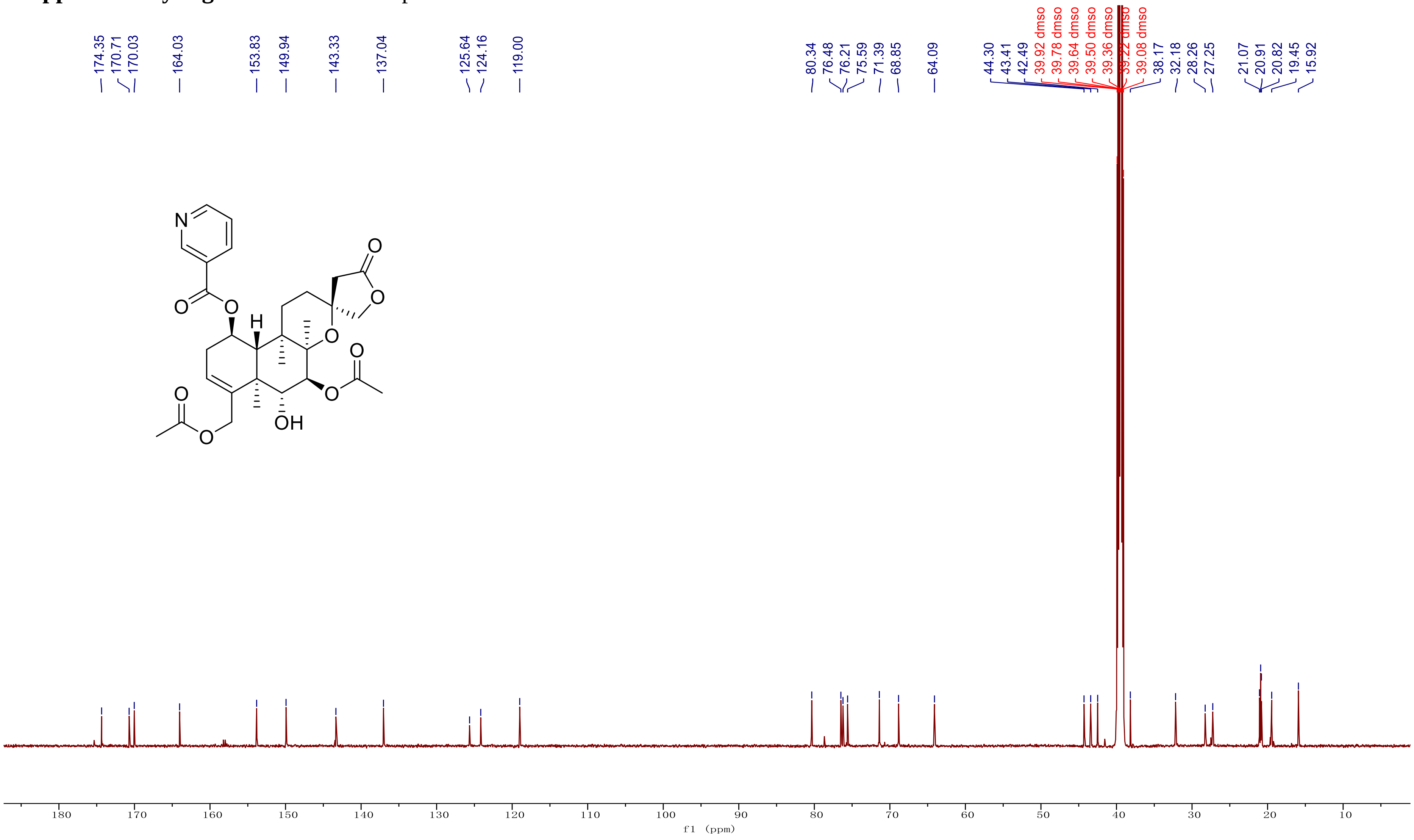

Supplementary Figure S4. DEPT spectrum of **2**

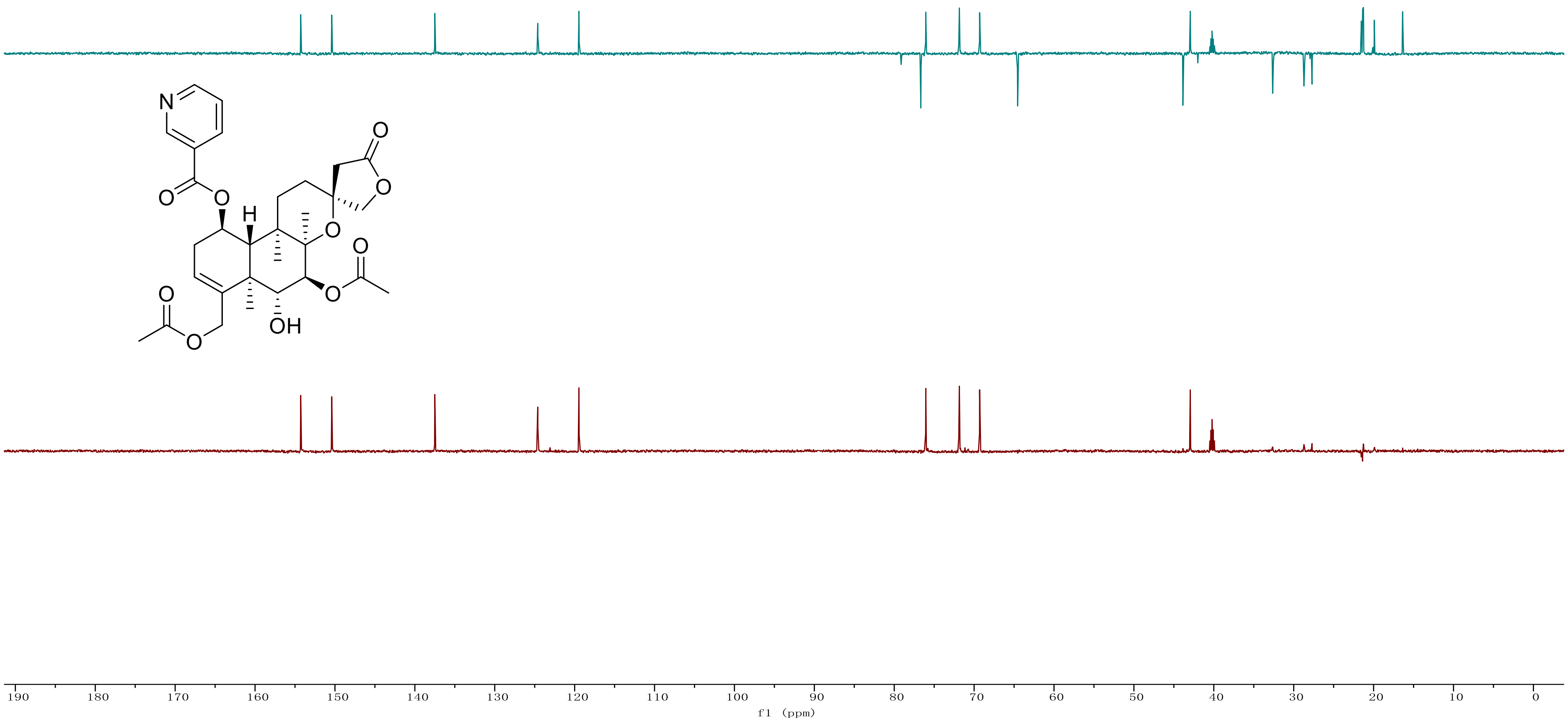

Supplementary Figure S5. ^1H–^1H COSY spectrum of **2**

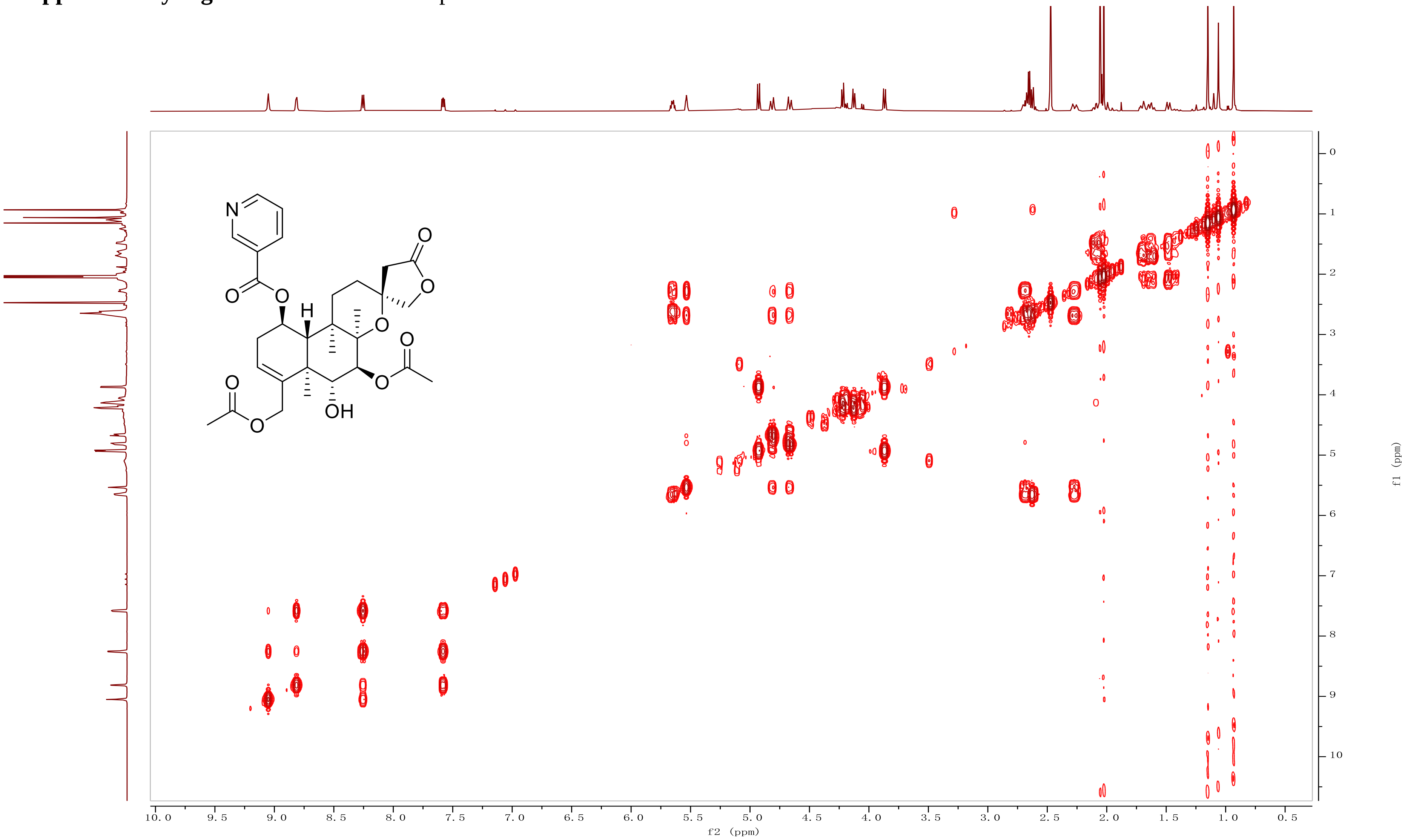

Supplementary Figure S6. HSQC spectrum of **2**

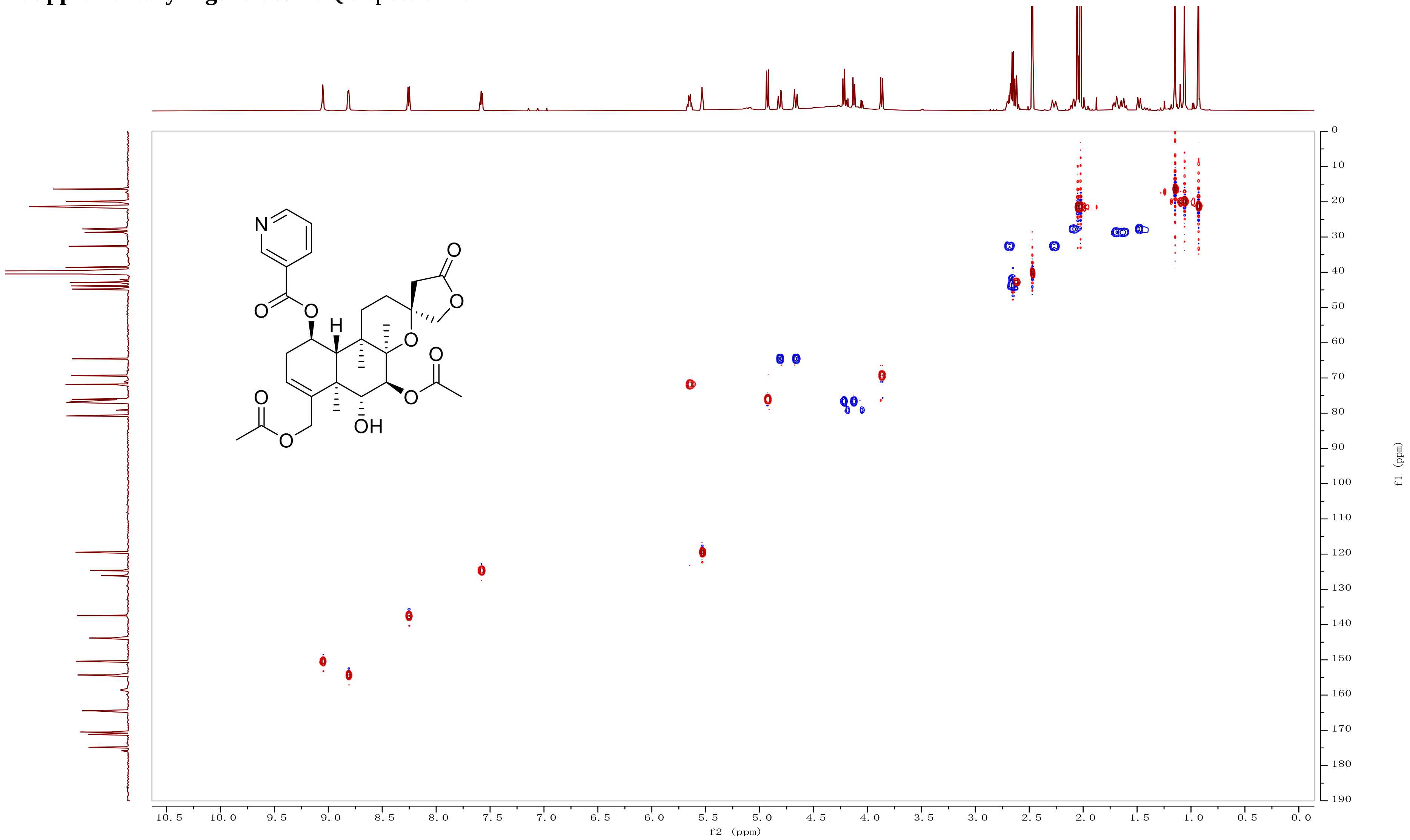

Supplementary Figure S7. HMBC spectrum of **2**

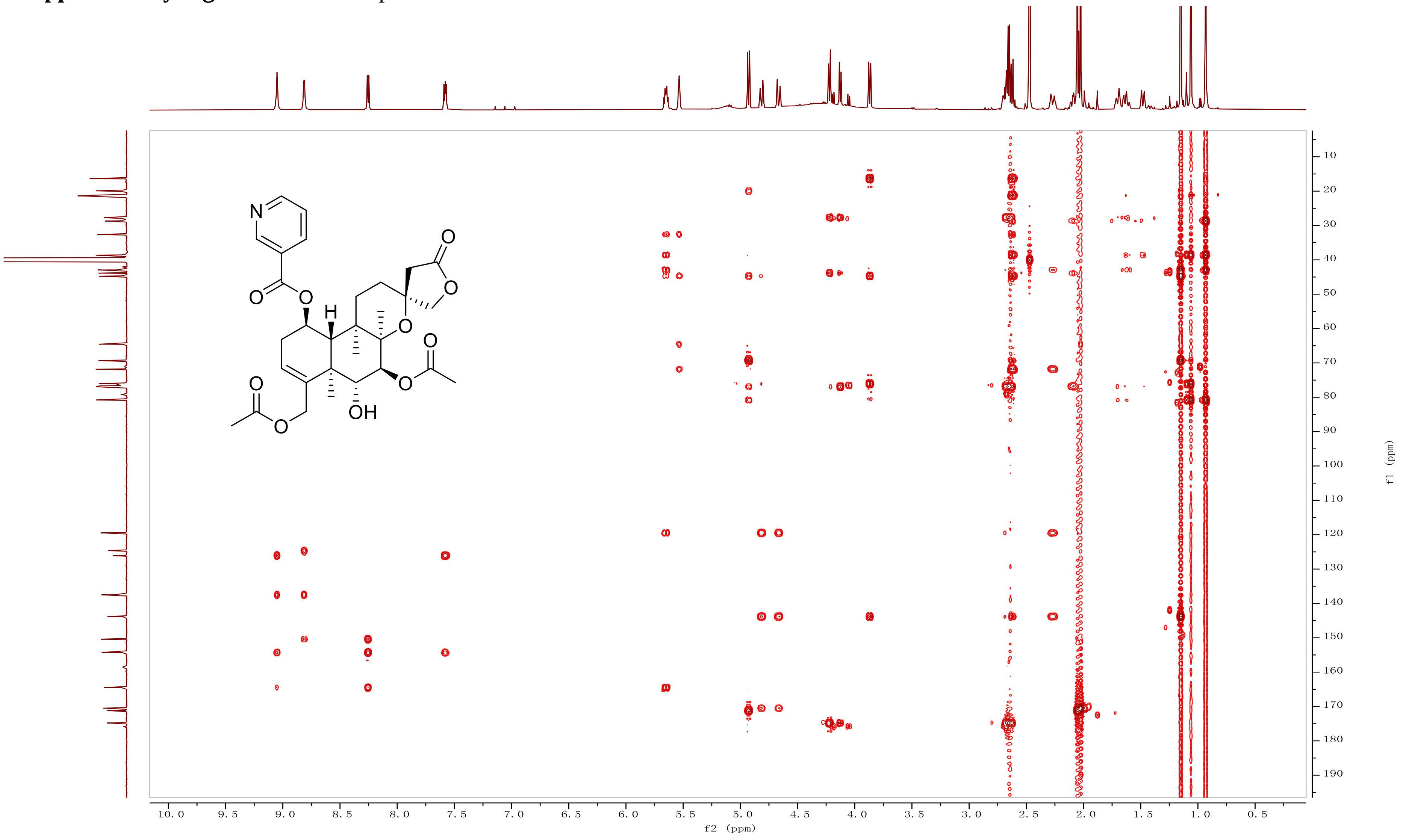

Supplementary Figure S8. NOESY spectrum of **2**

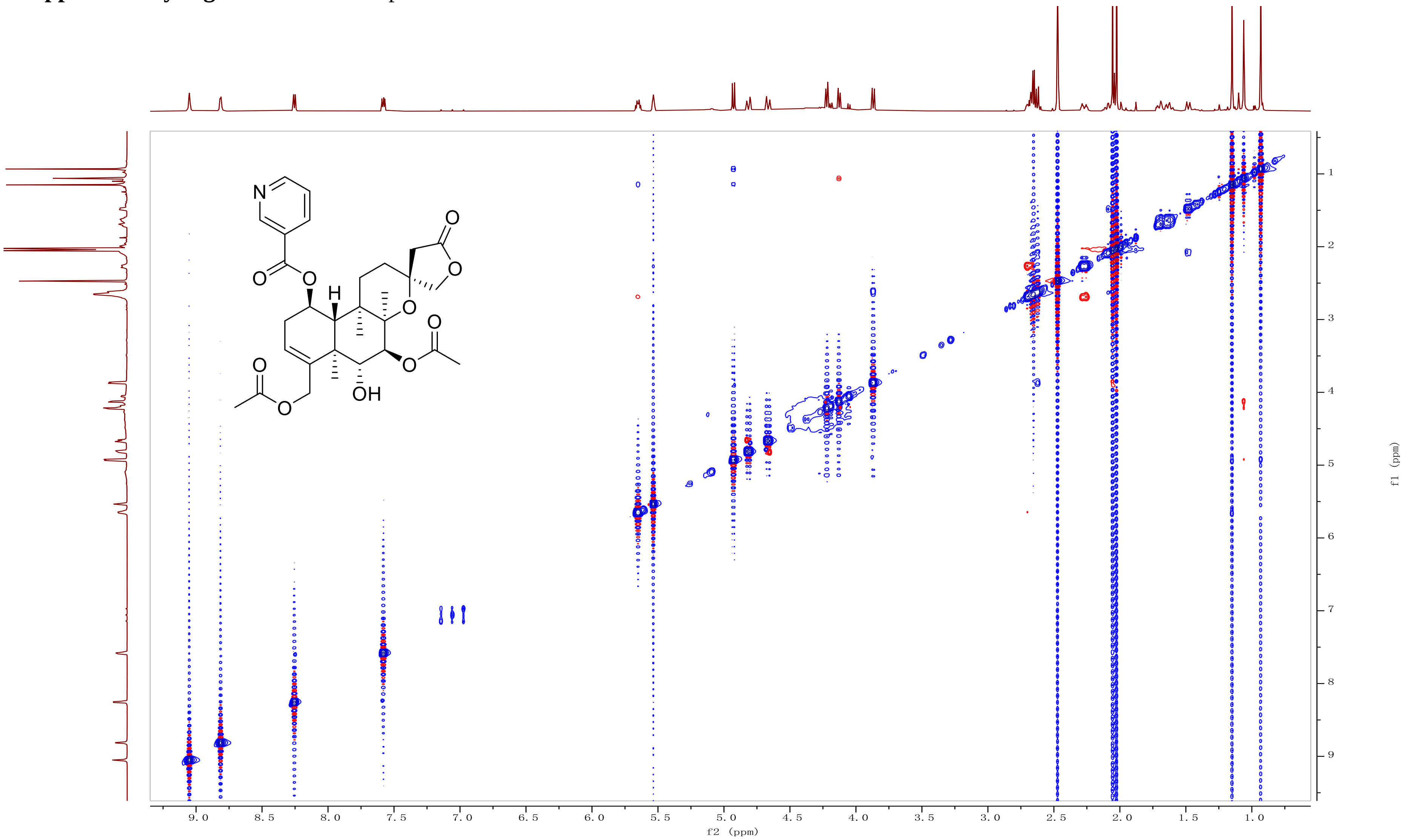

Supplementary Figure S9. HRESIMS spectrum of **2**

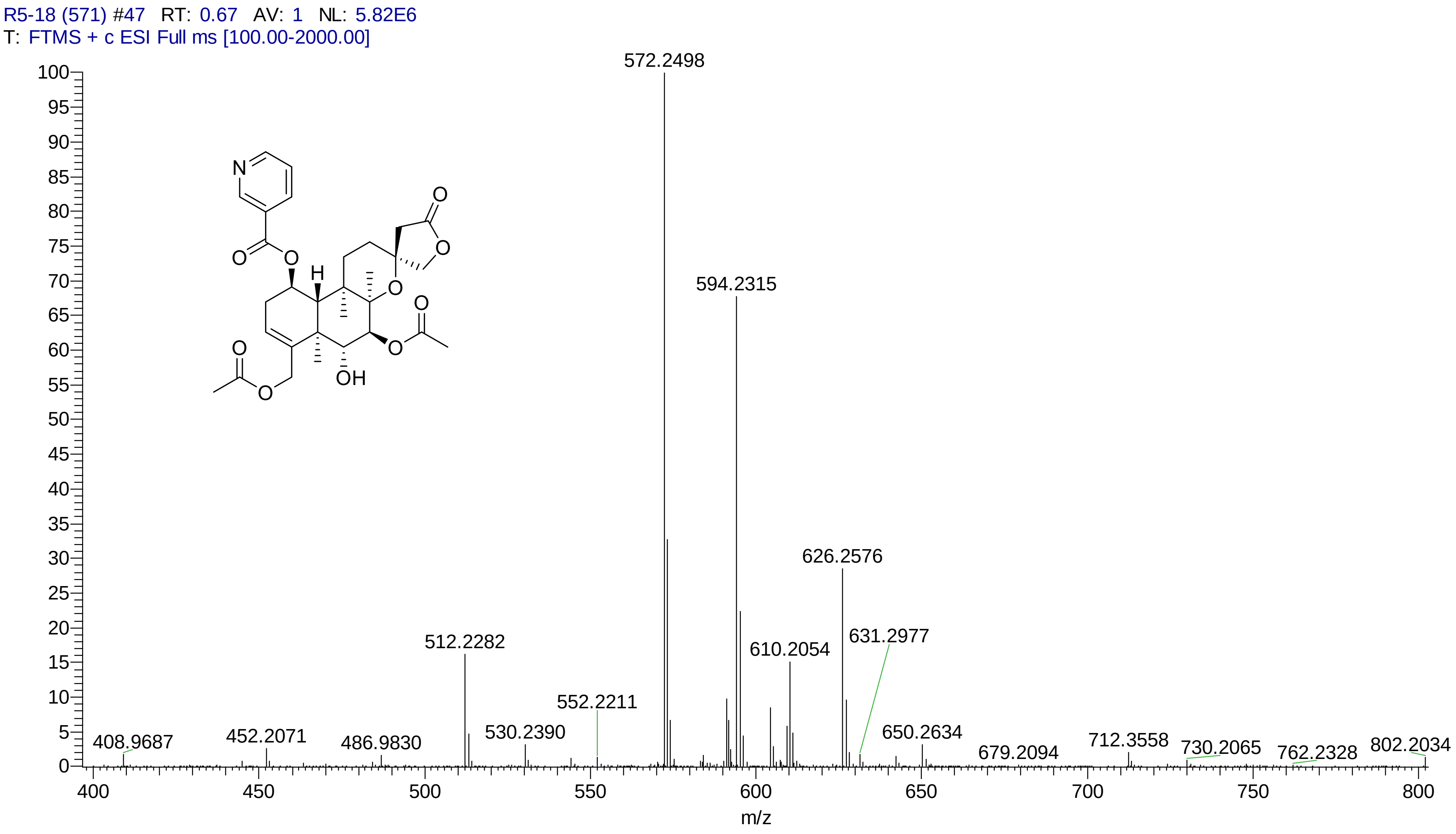

Supplementary Figure S10. IR spectrum of **2**

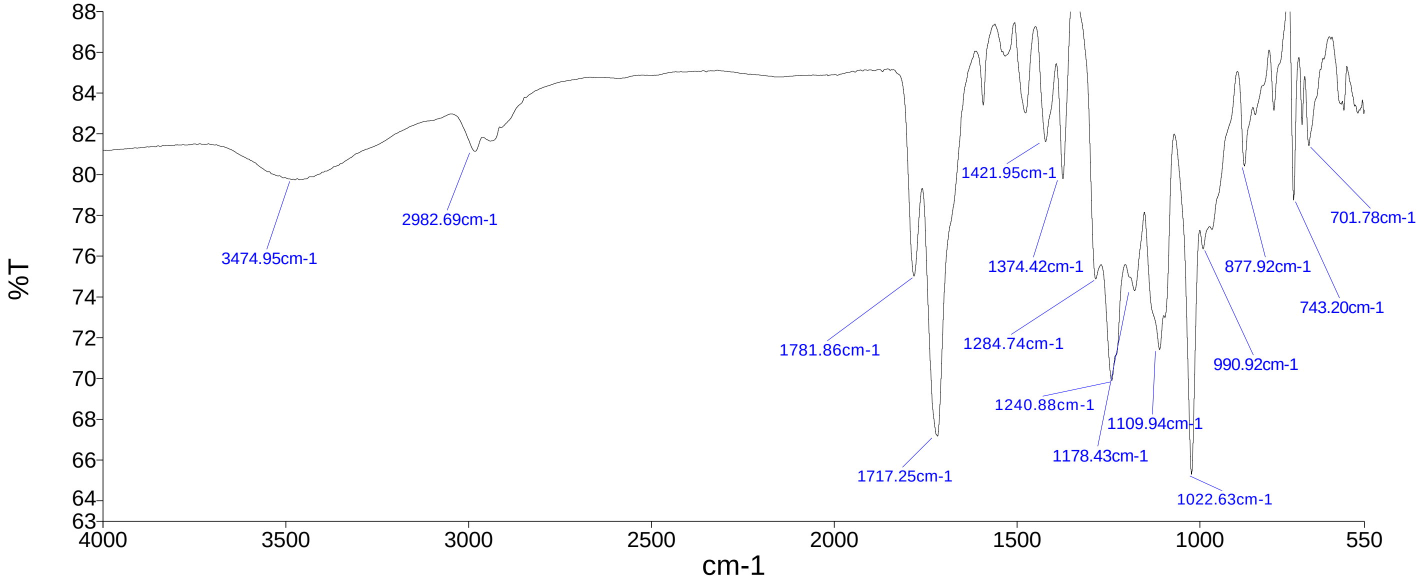

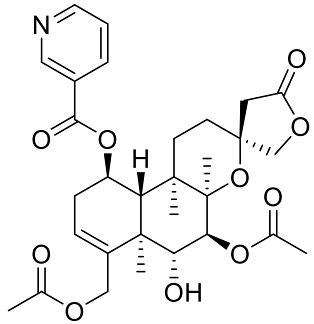

Supplementary Figure S11. UV spectrum of **2**

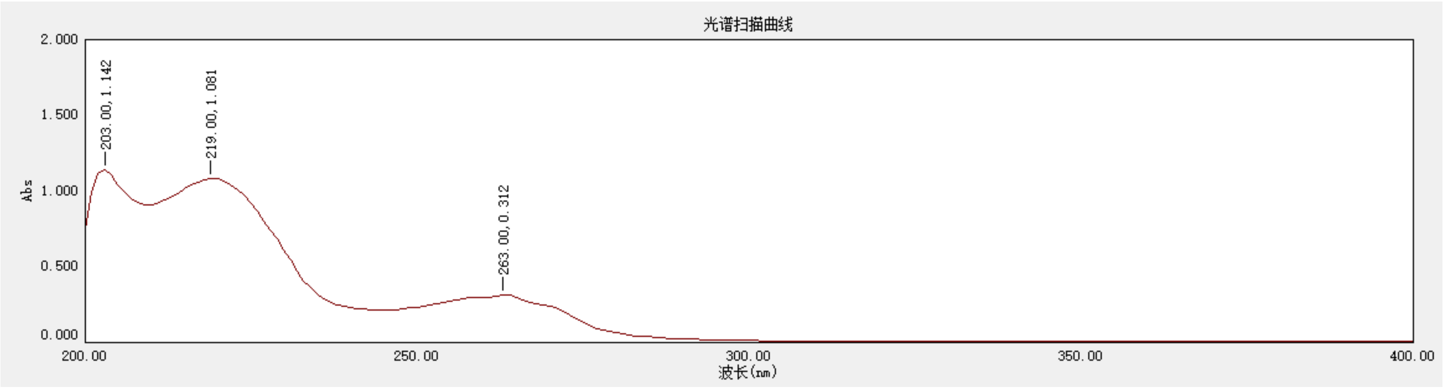

Supplementary Figure S12. CD spectrum of **2**

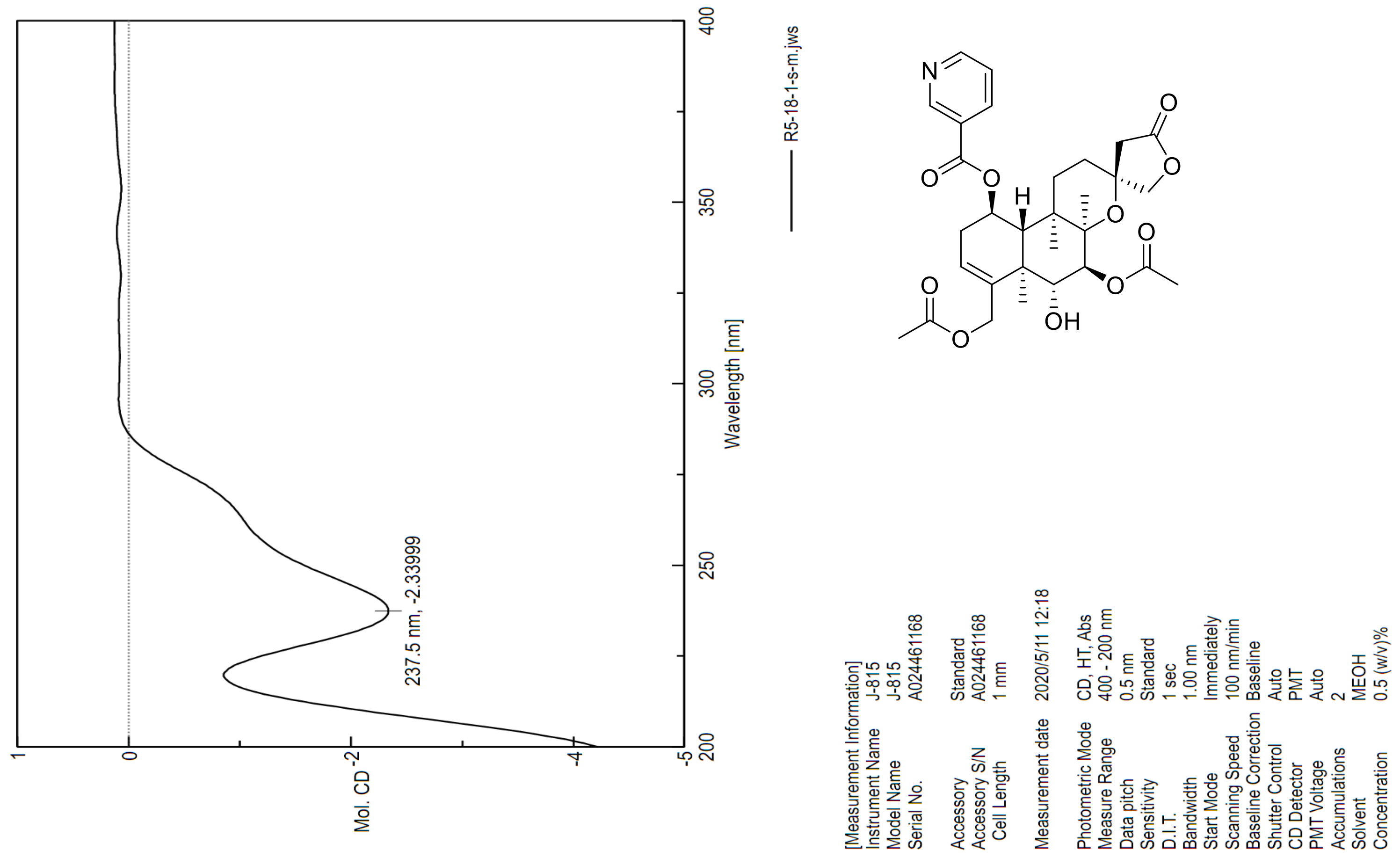

Supplementary Figure S13. ^{1}H NMR spectrum of **3**

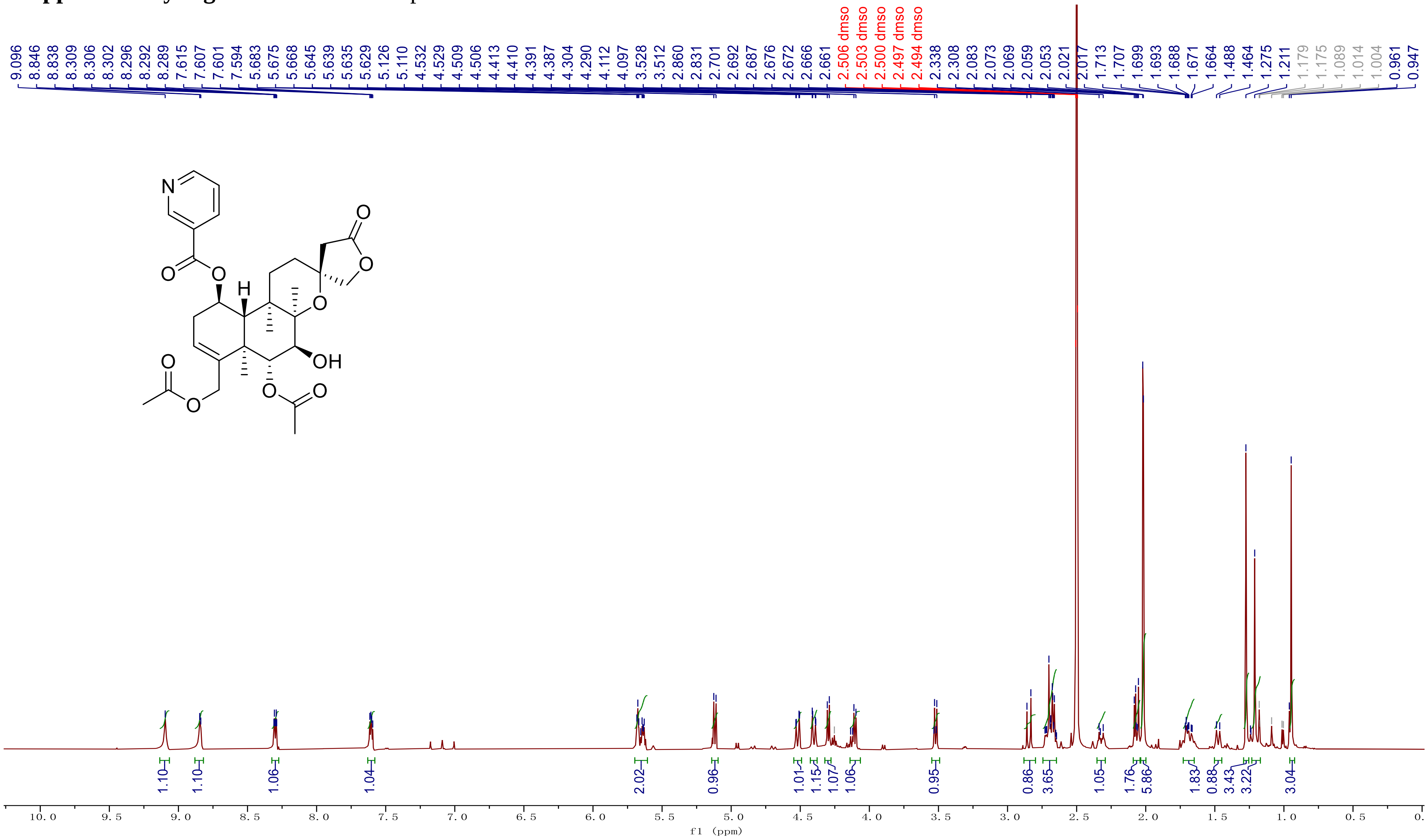

Supplementary Figure S14. ^{13}C NMR spectrum of **3**

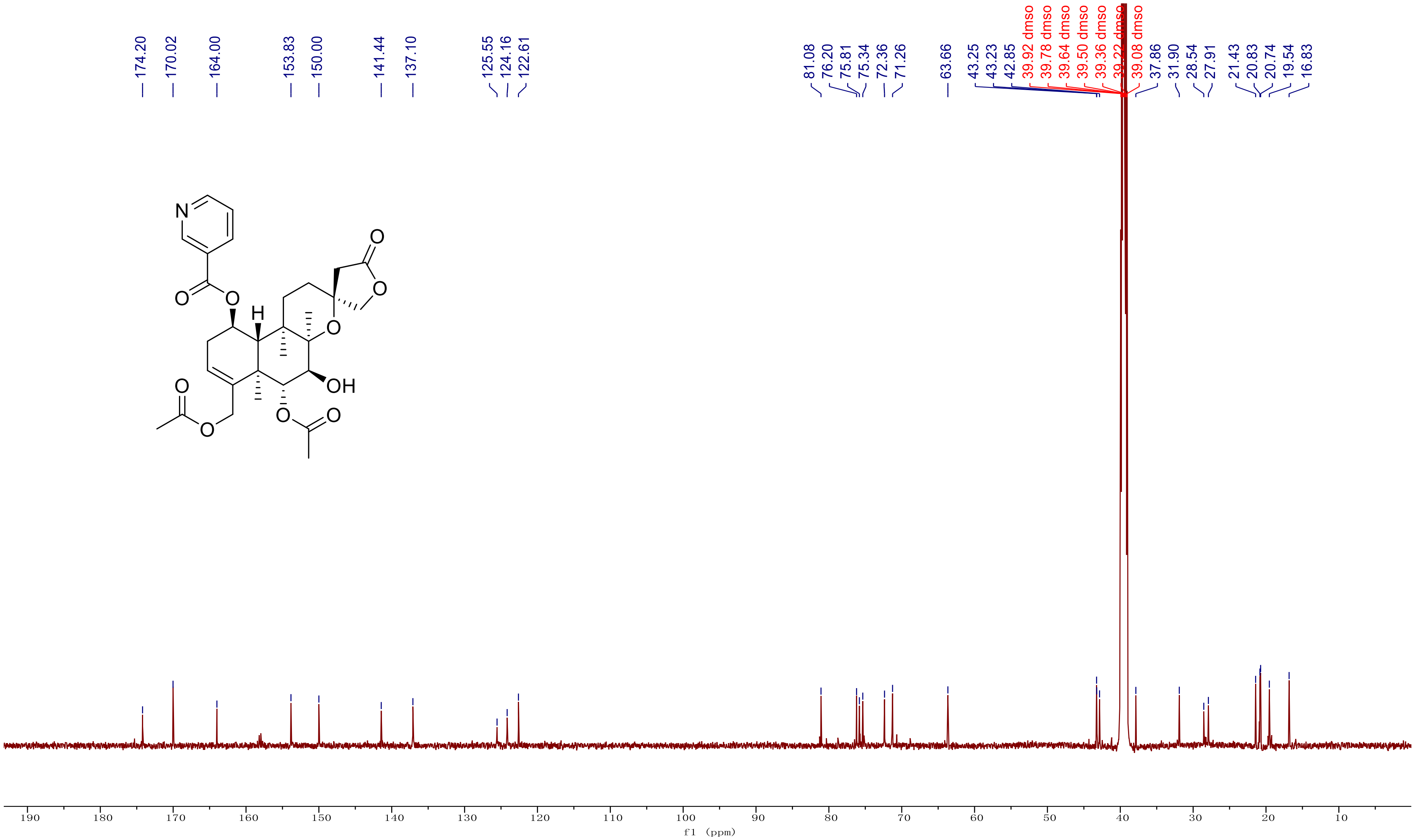

Supplementary Figure S15. DEPT spectrum of **3**

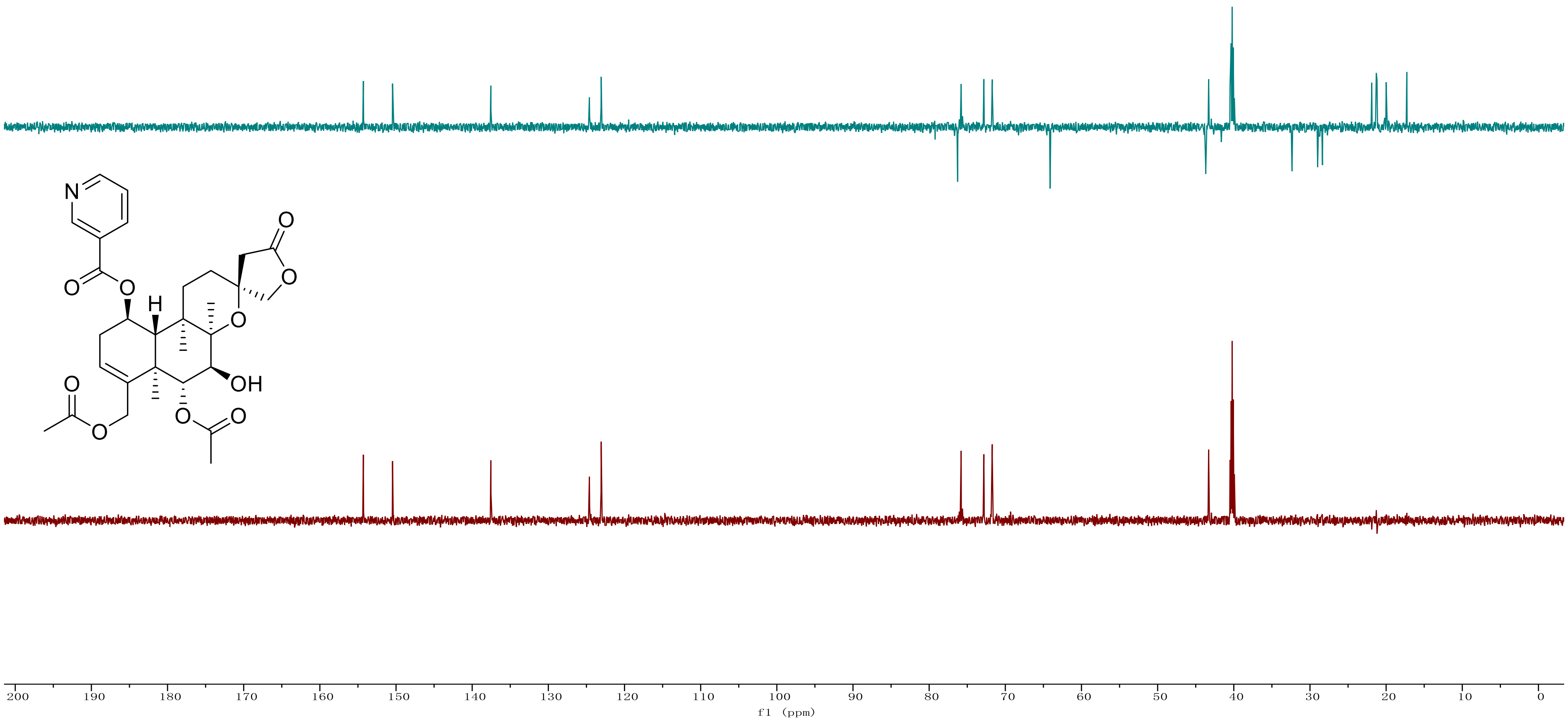

Supplementary Figure S16. ^{1}H–^{1}H COSY spectrum of **3**

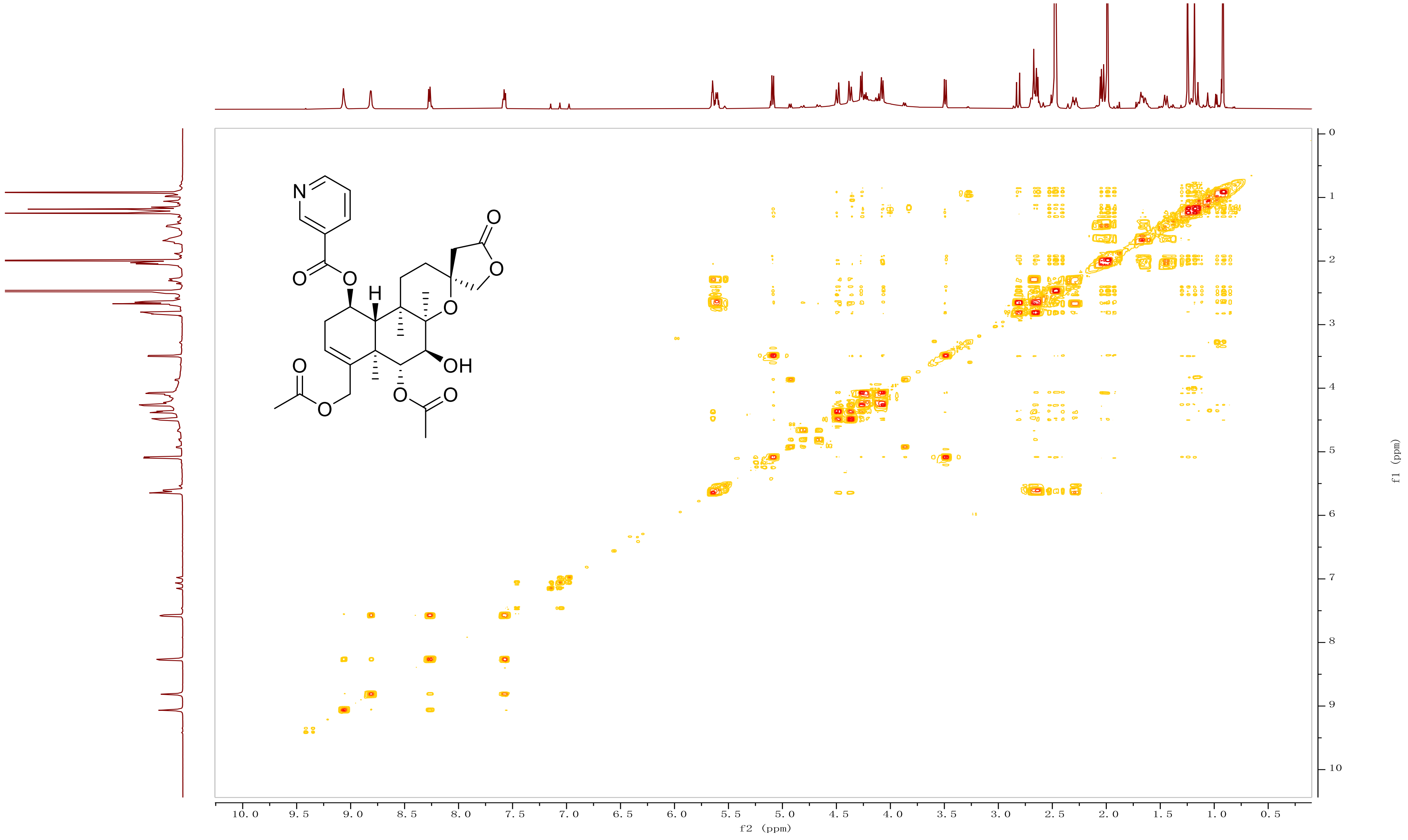

Supplementary Figure S17. HSQC spectrum of **3**

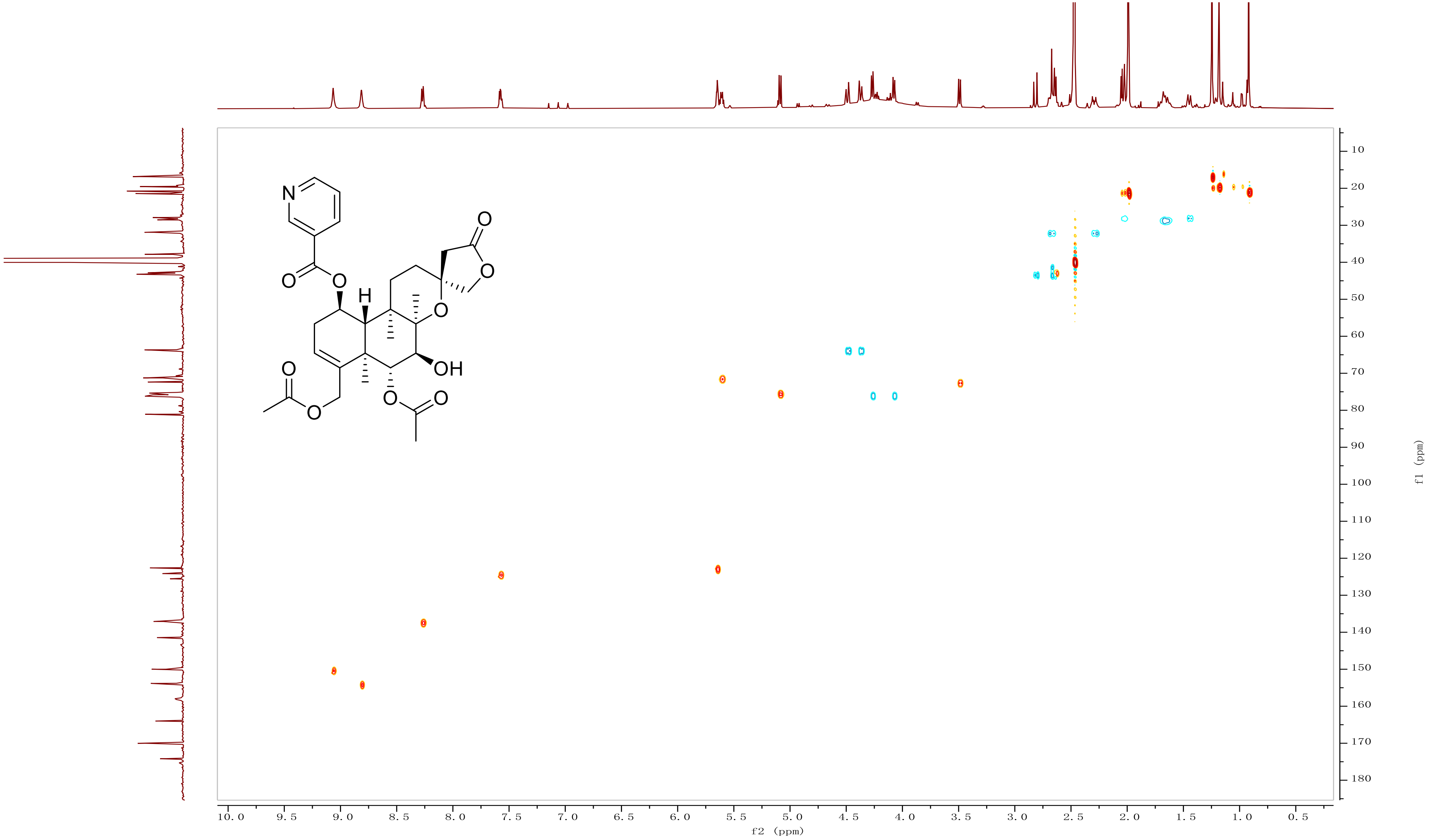

Supplementary Figure S18. HMBC spectrum of **3**

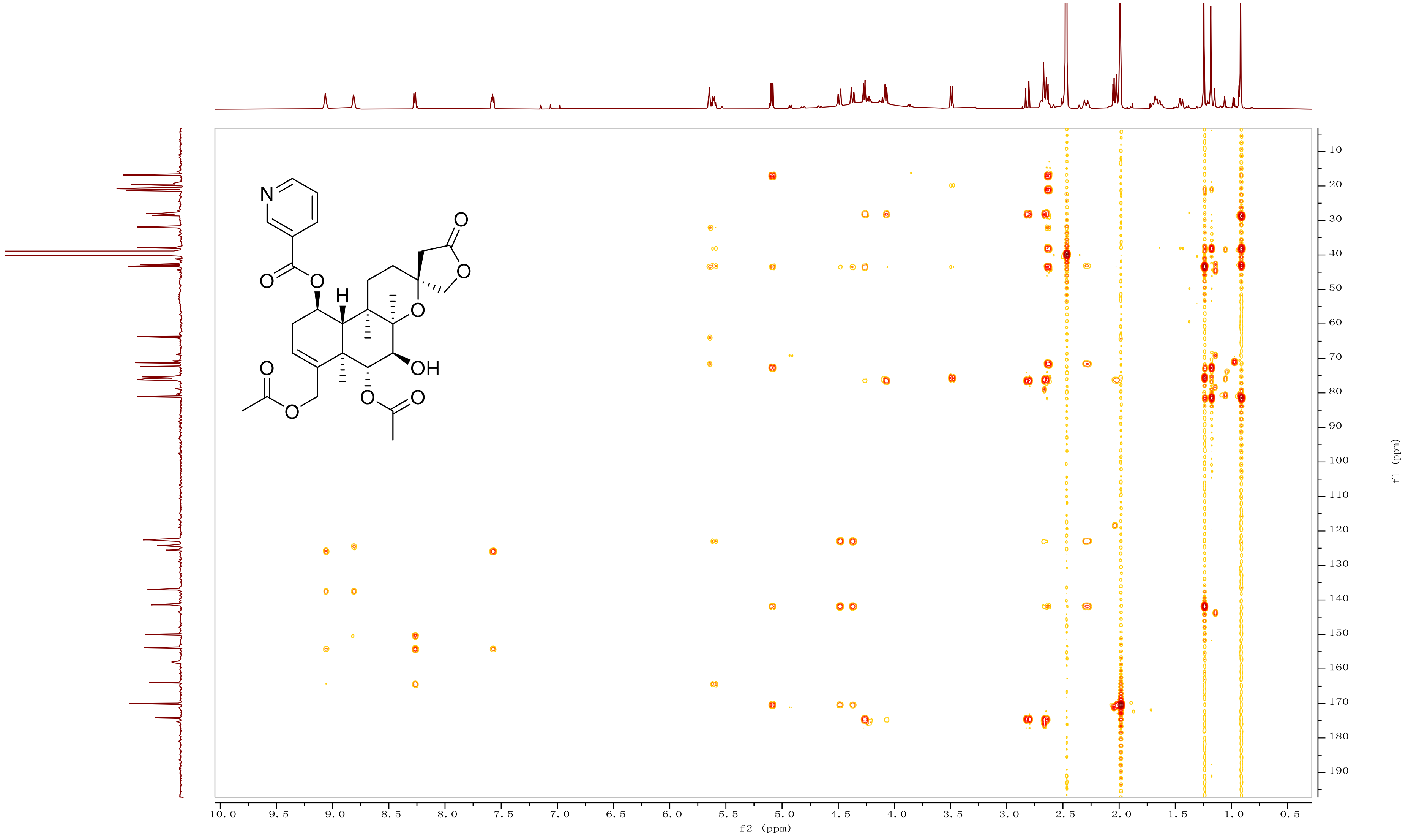

Supplementary Figure S19. NOESY spectrum of **3**

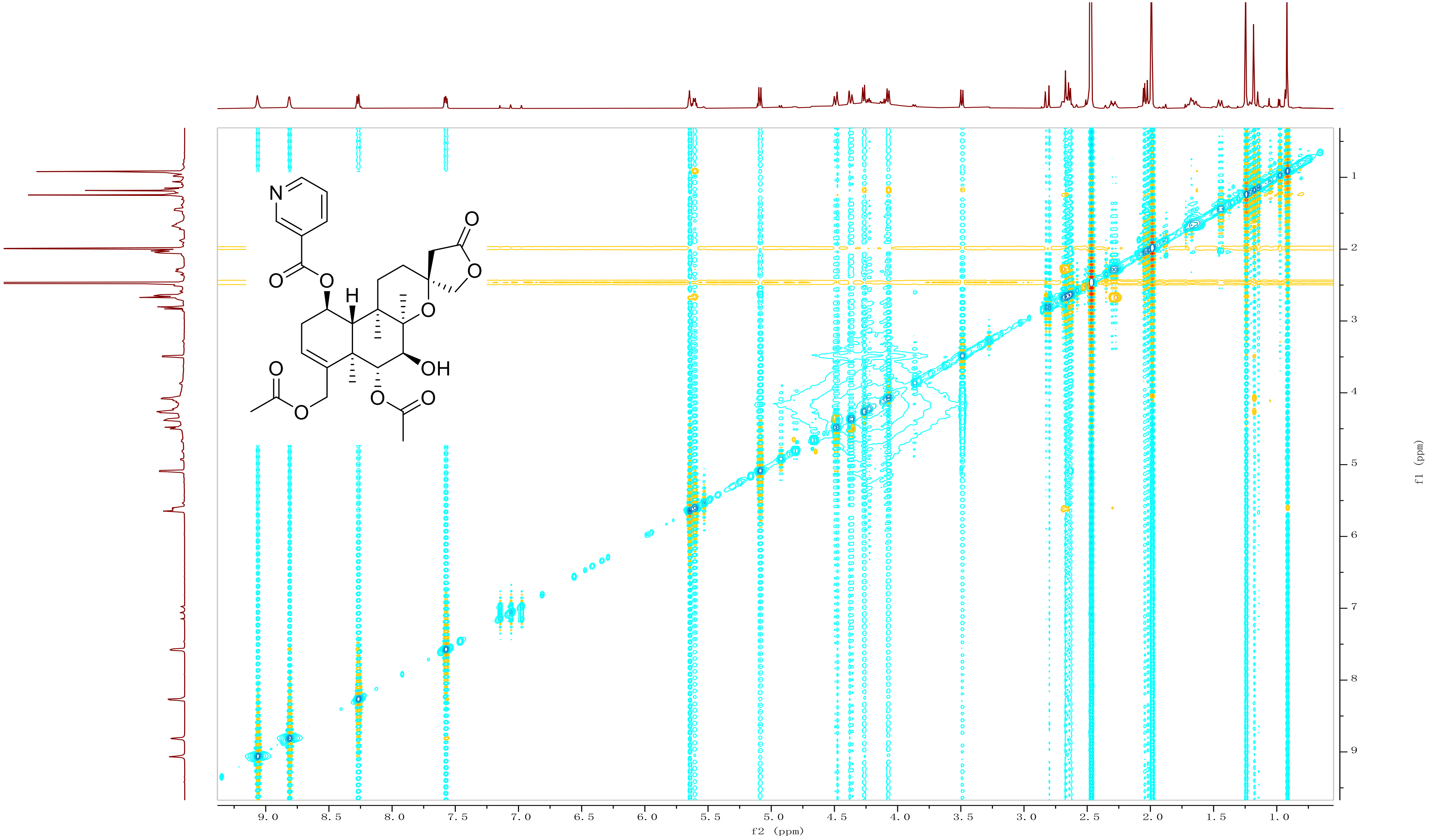

Supplementary Figure S20. HRESIMS spectrum of **3**

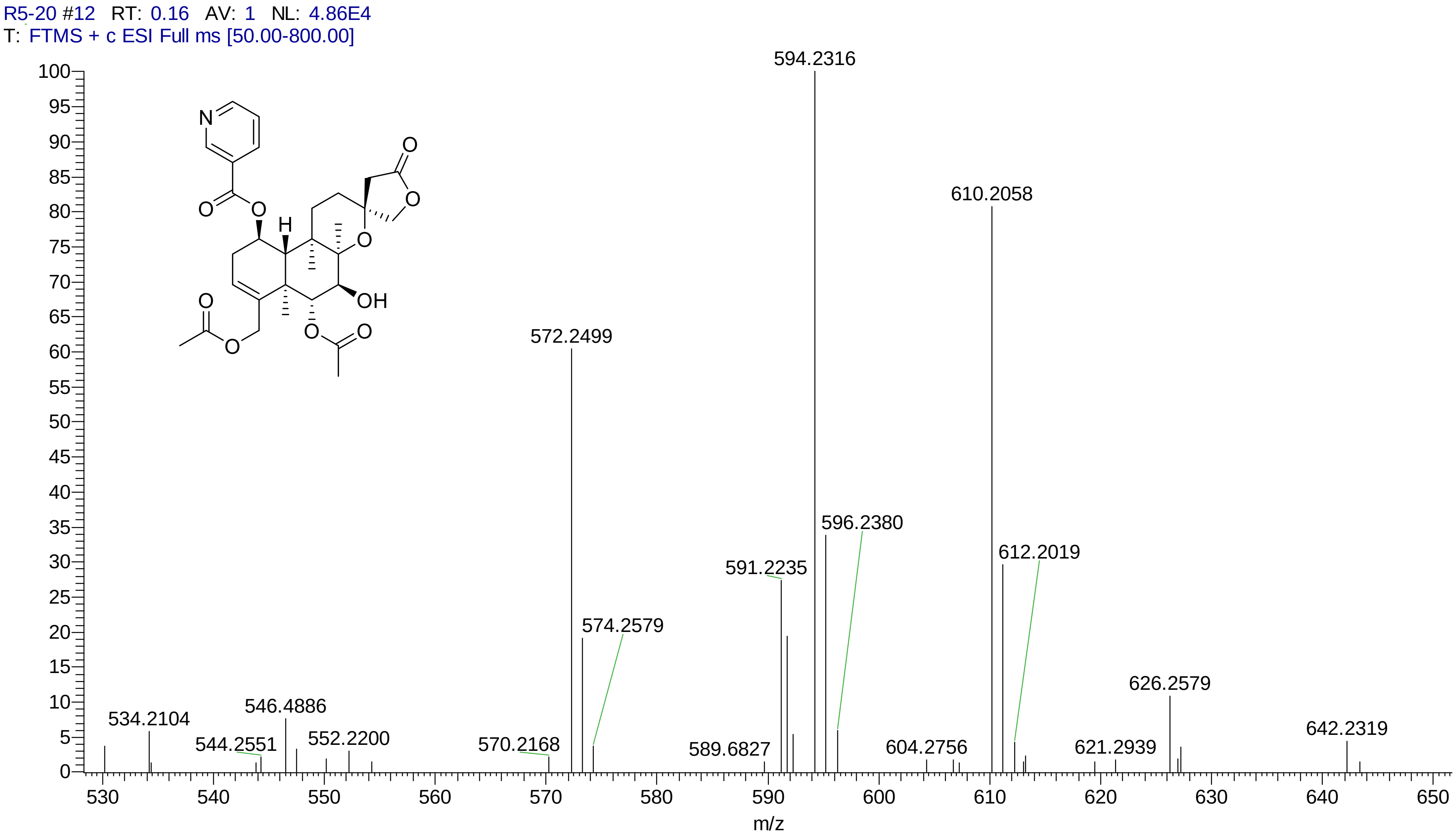

Supplementary Figure S21. IR spectrum of **3**

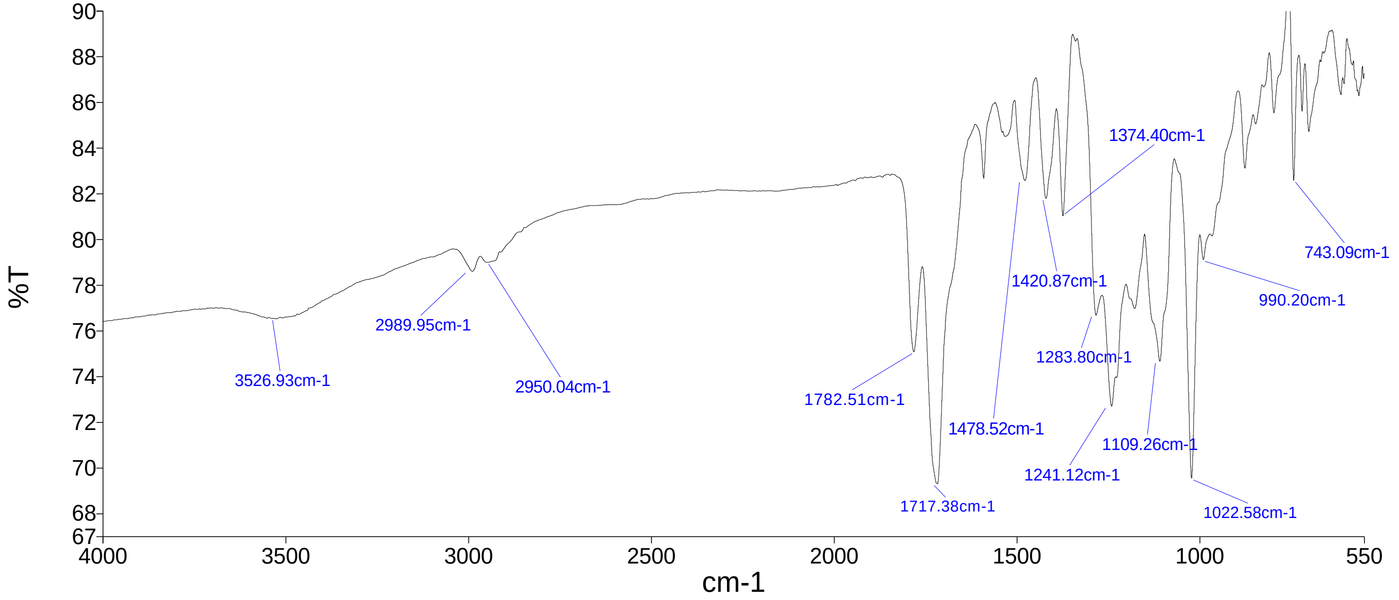

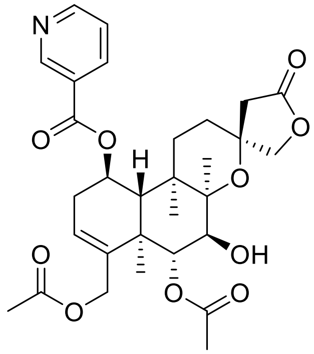

Supplementary Figure S22. UV spectrum of **3**

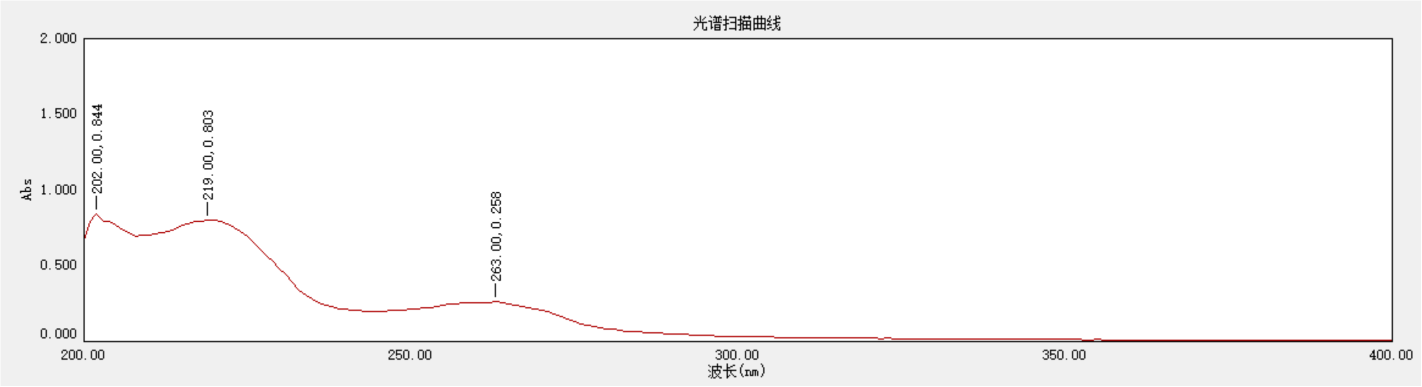

Supplementary Figure S23. CD spectrum of **3**

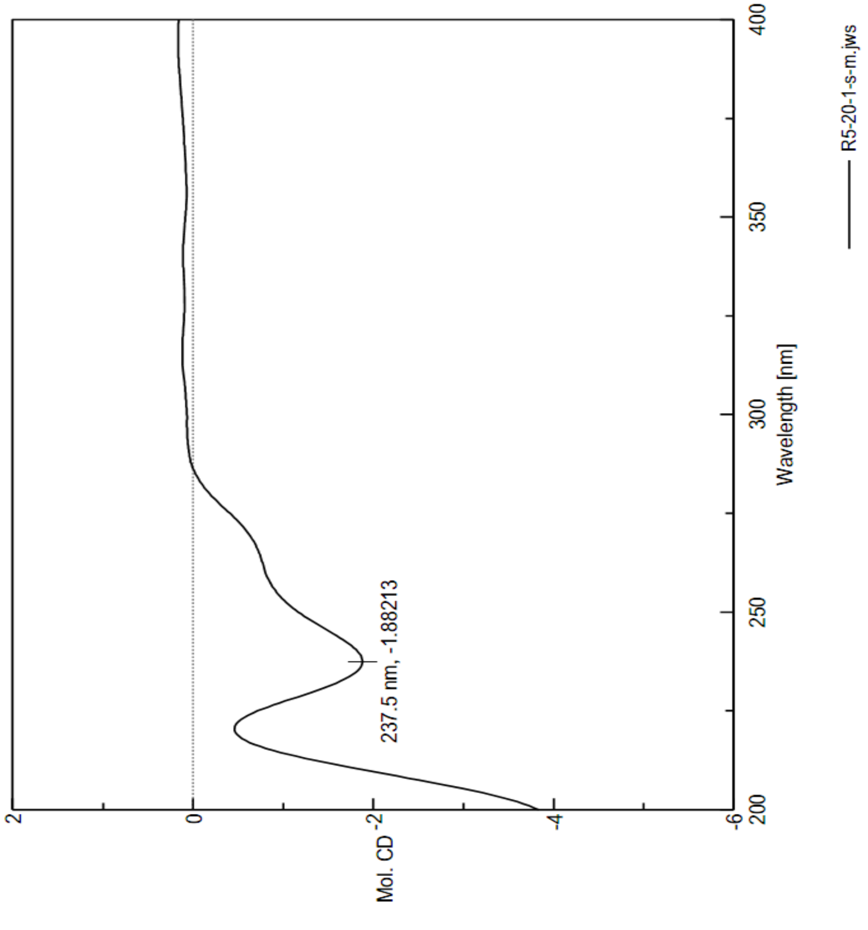

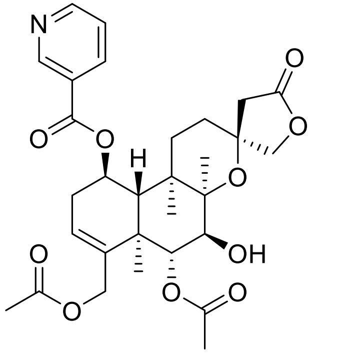

Supplementary Figure S24. ^{1}H NMR spectrum of **4**

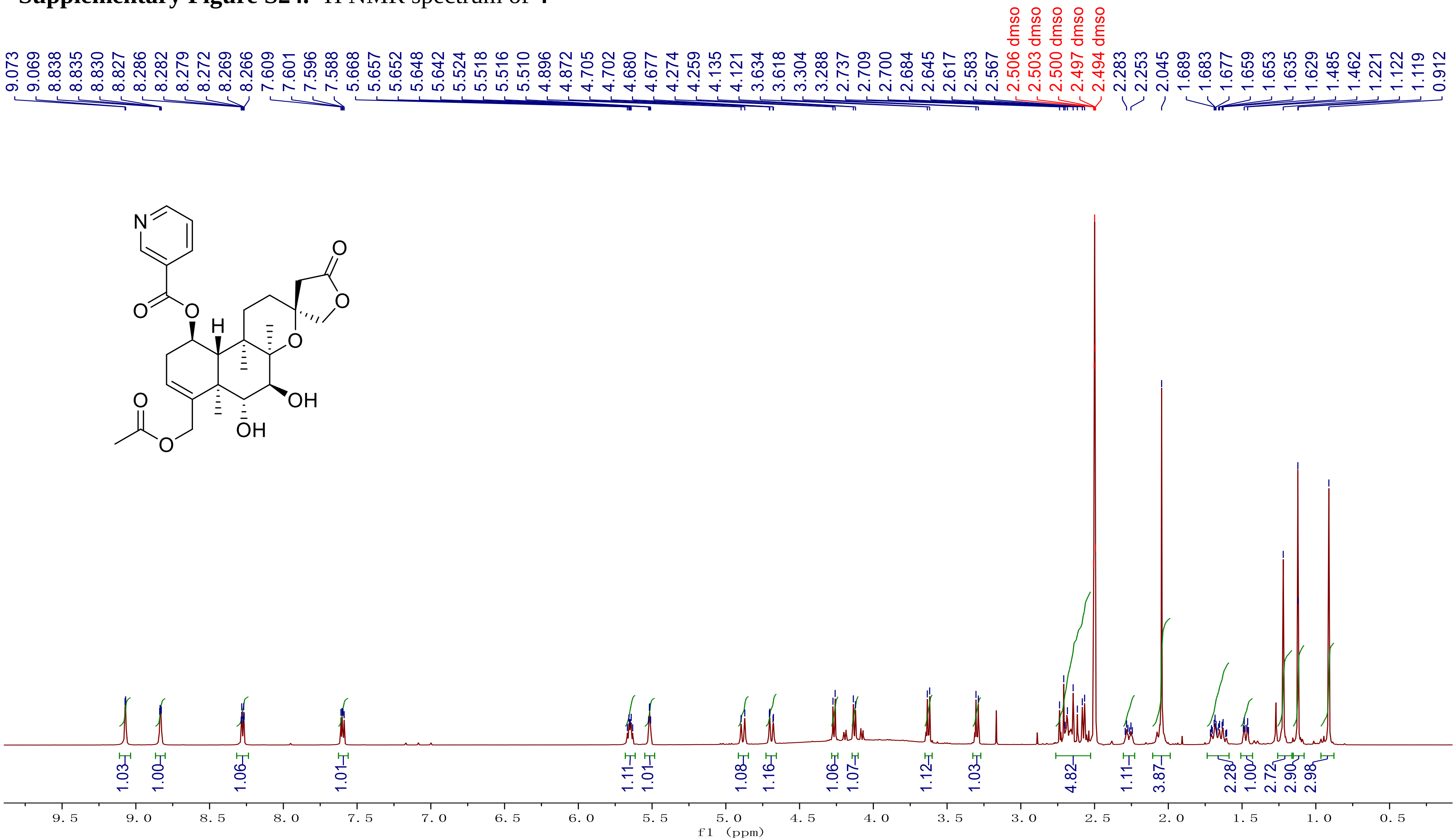

Supplementary Figure S25. ^{13}C NMR spectrum of **4**

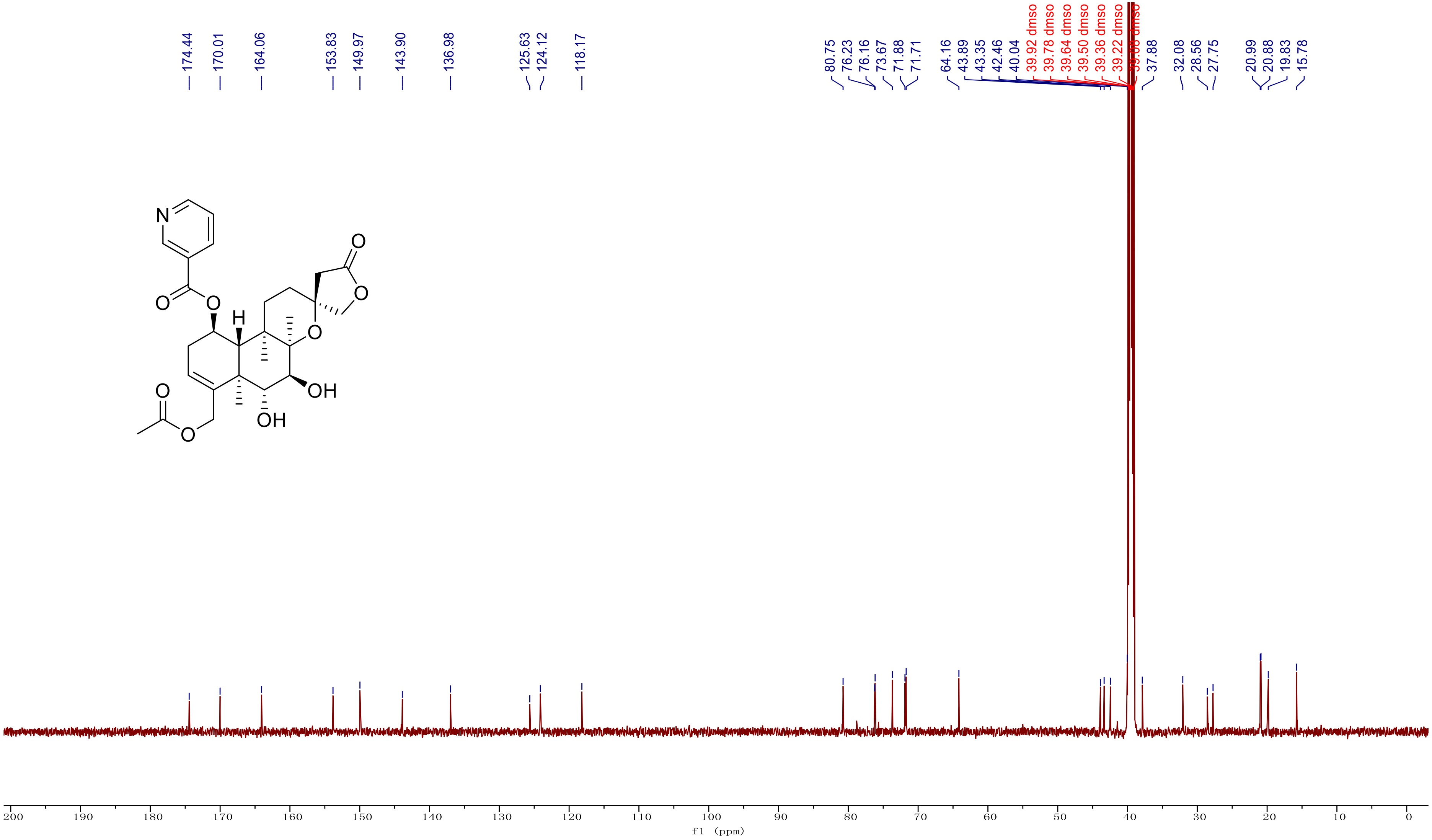

Supplementary Figure S26. DEPT spectrum of **4**

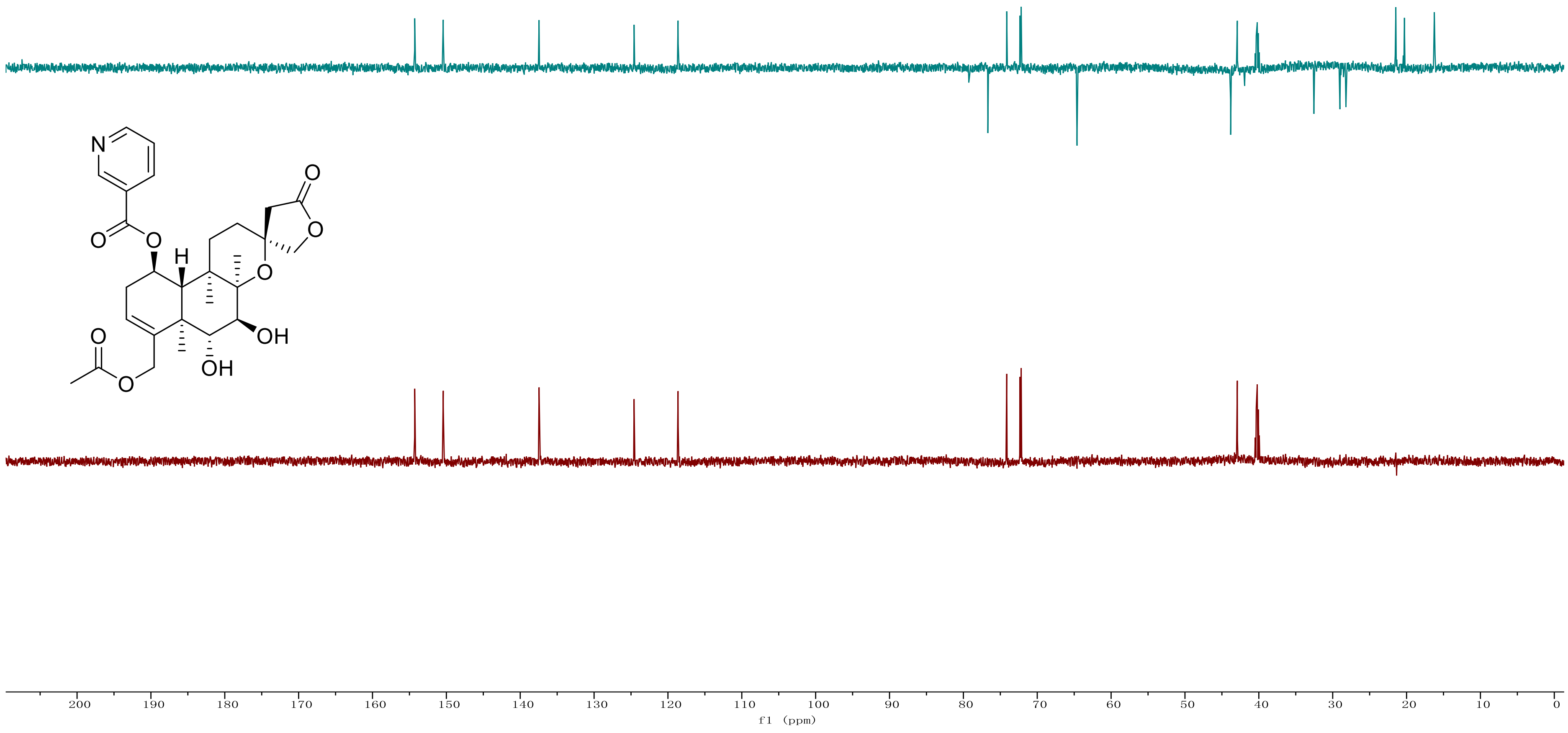

Supplementary Figure S27. ^1H–^1H COSY spectrum of **4**

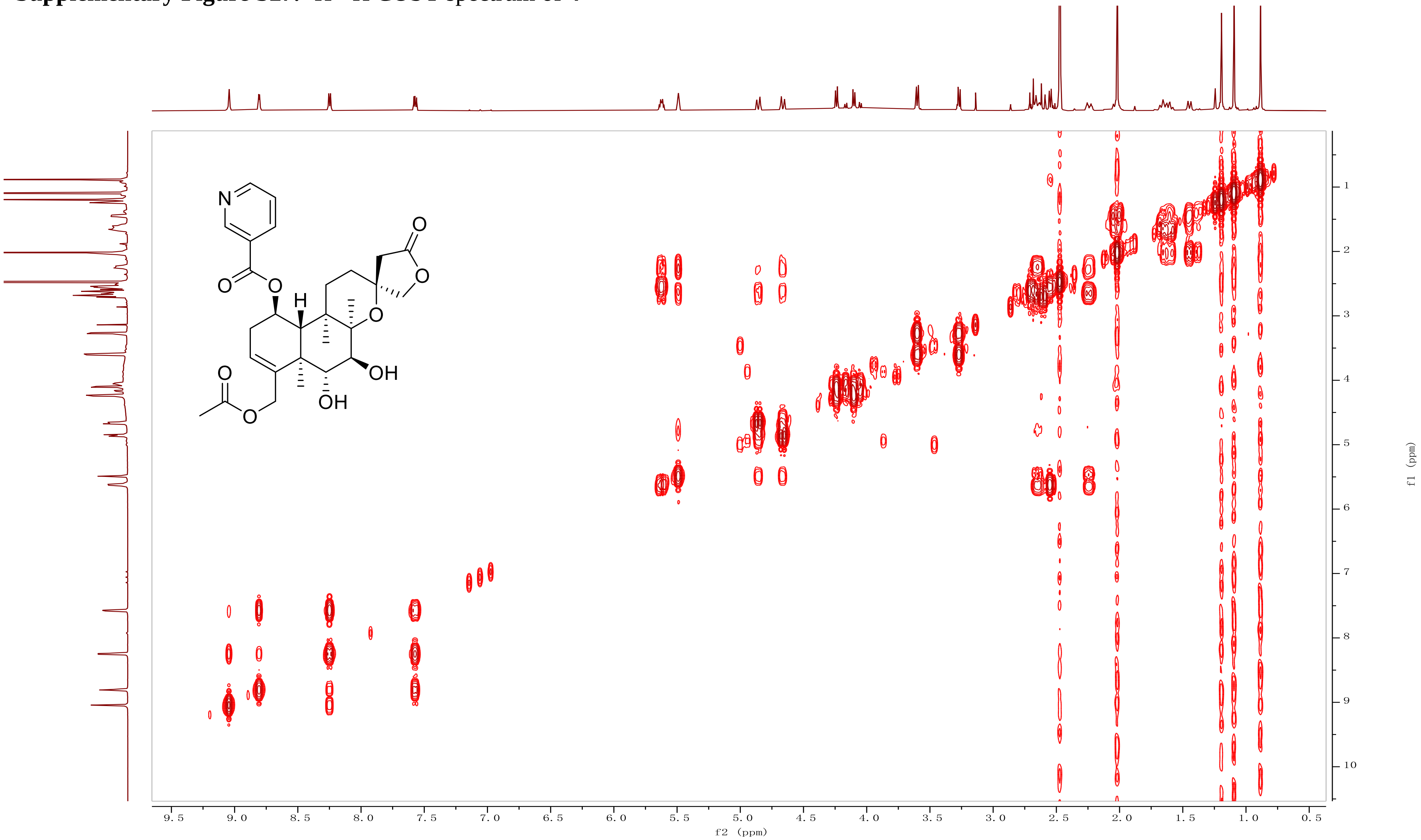

Supplementary Figure S28. HSQC spectrum of **4**

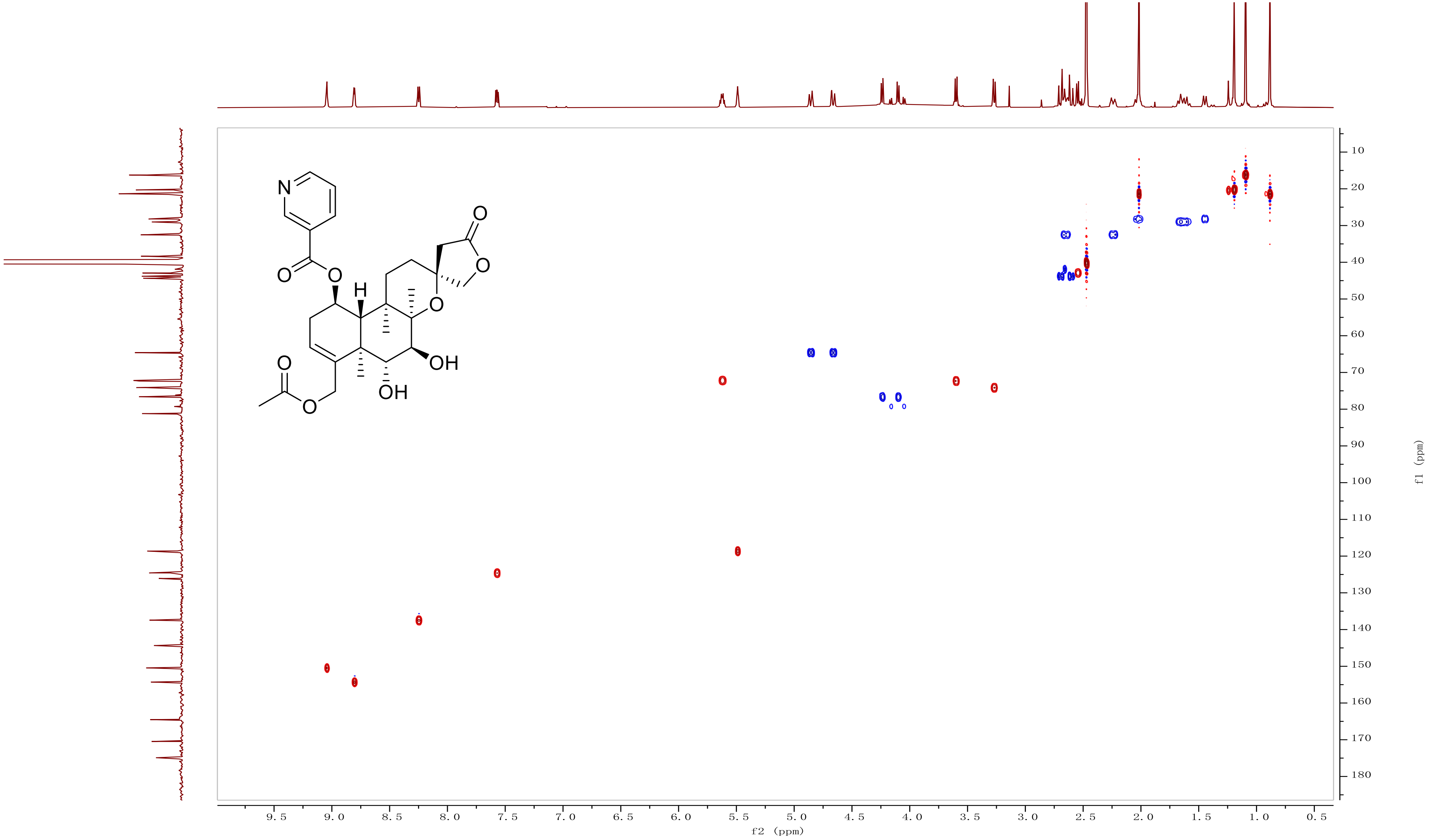

Supplementary Figure S29. HMBC spectrum of **4**

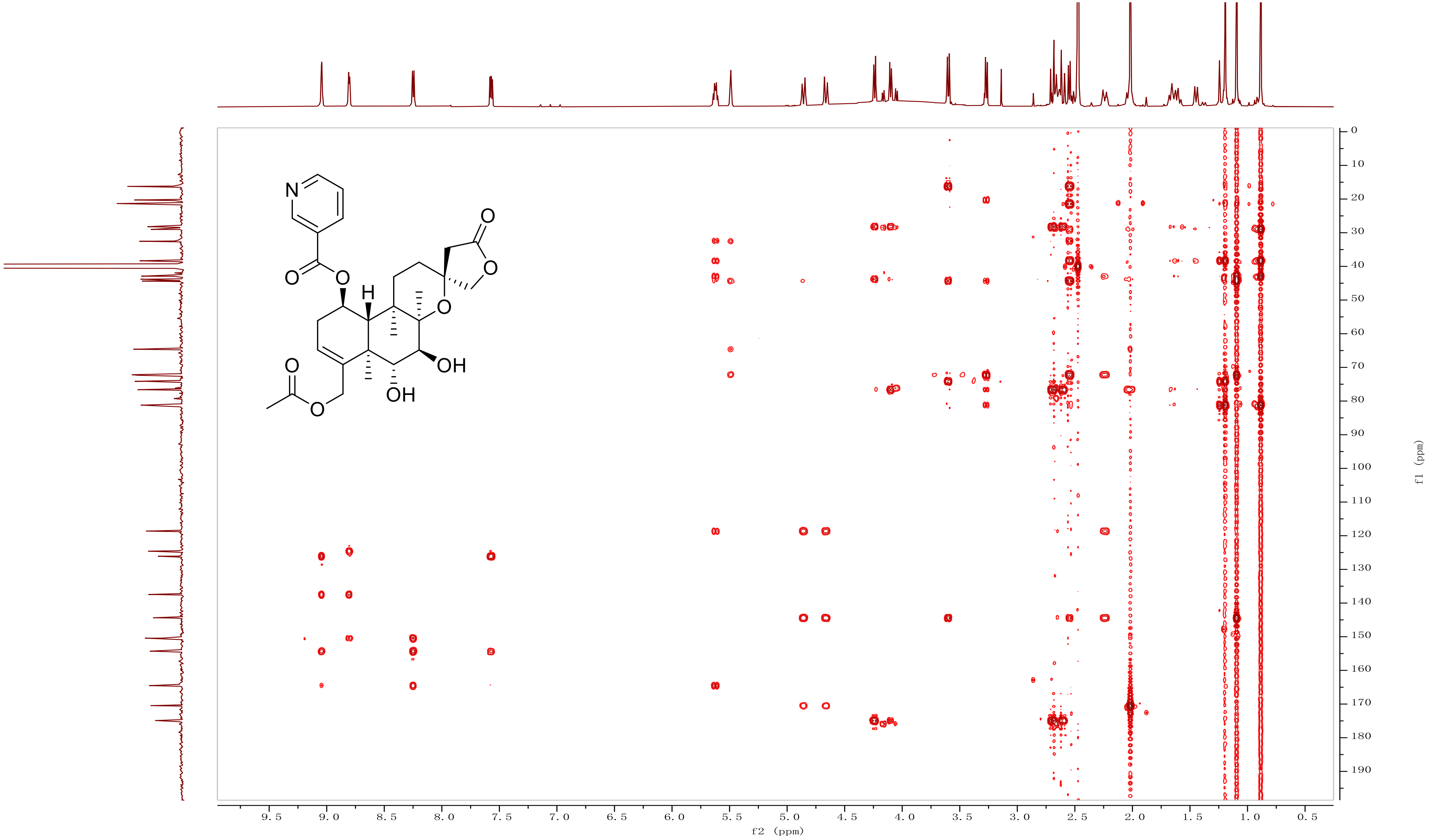

Supplementary Figure S30. 1D NOE spectrum of **4**

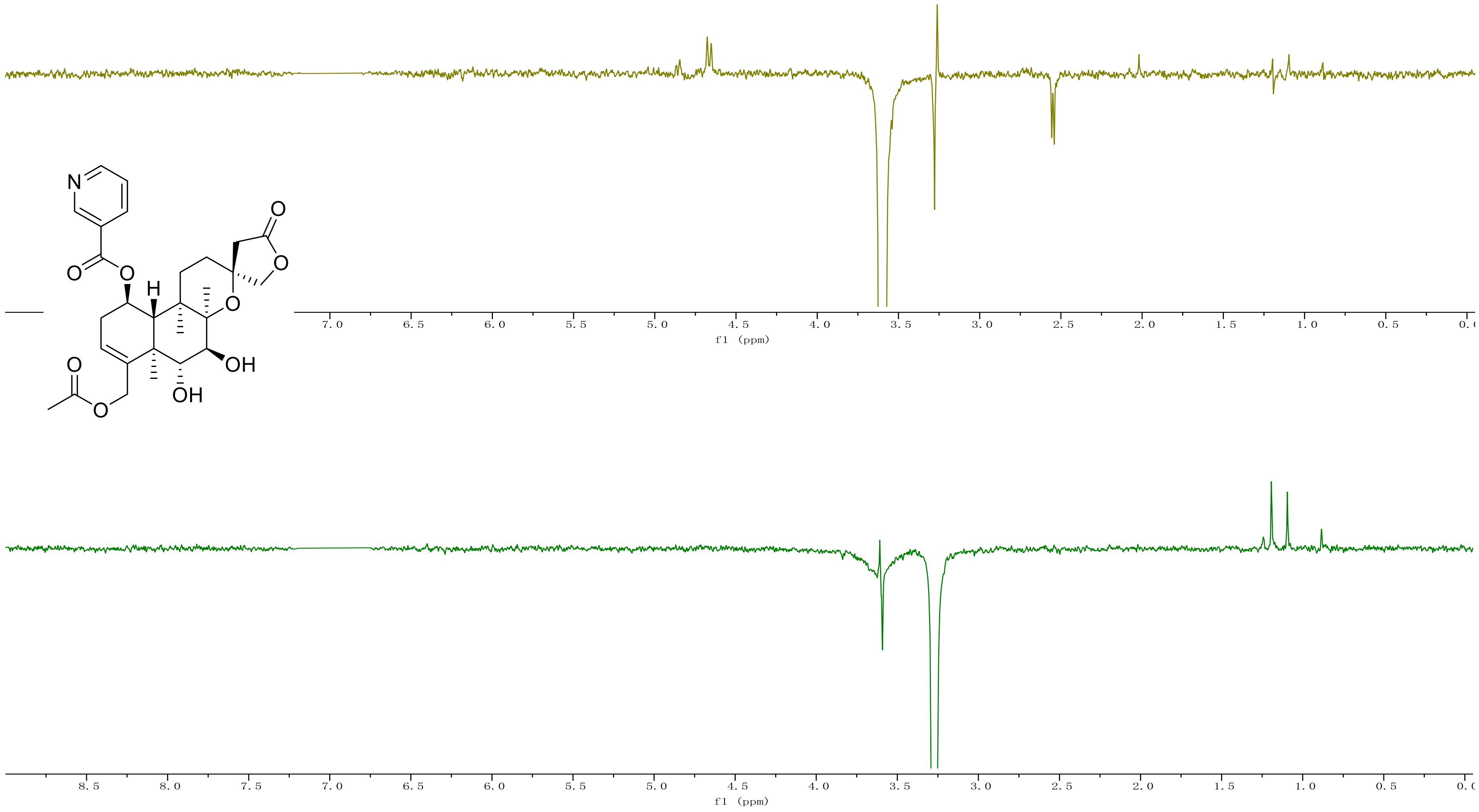

Supplementary Figure S31. HRESIMS spectrum of **4**

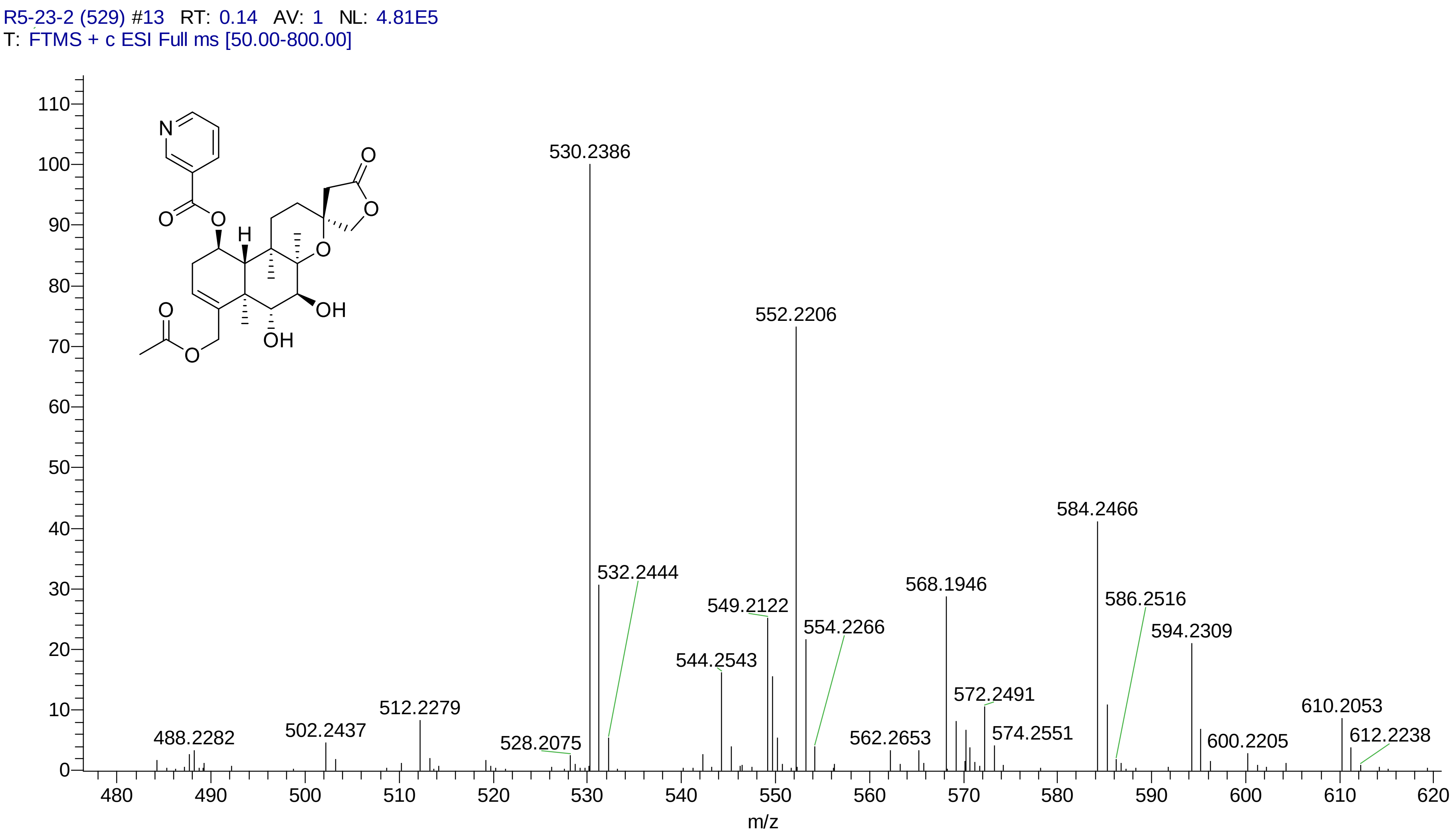

Supplementary Figure S32. IR spectrum of **4**

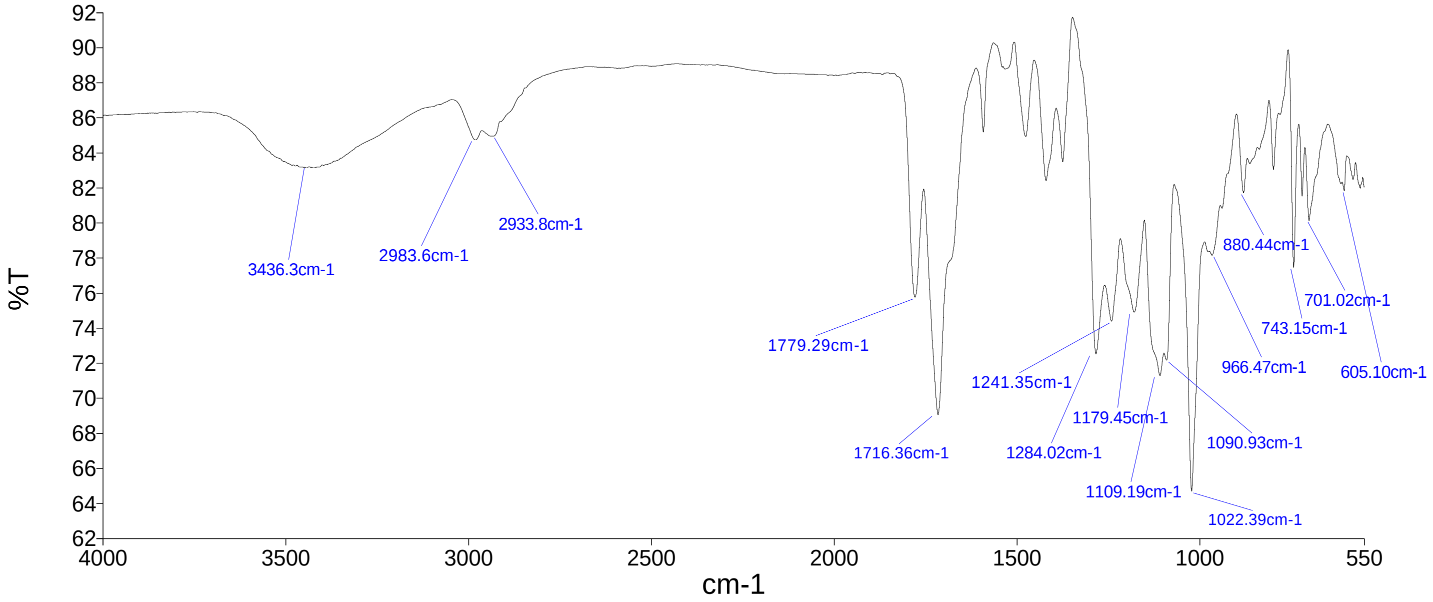

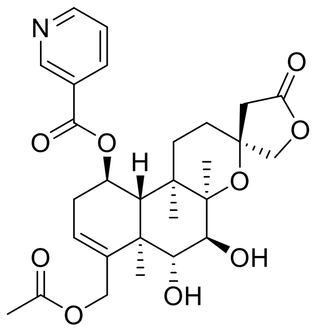

Supplementary Figure S33. UV spectrum of **4**

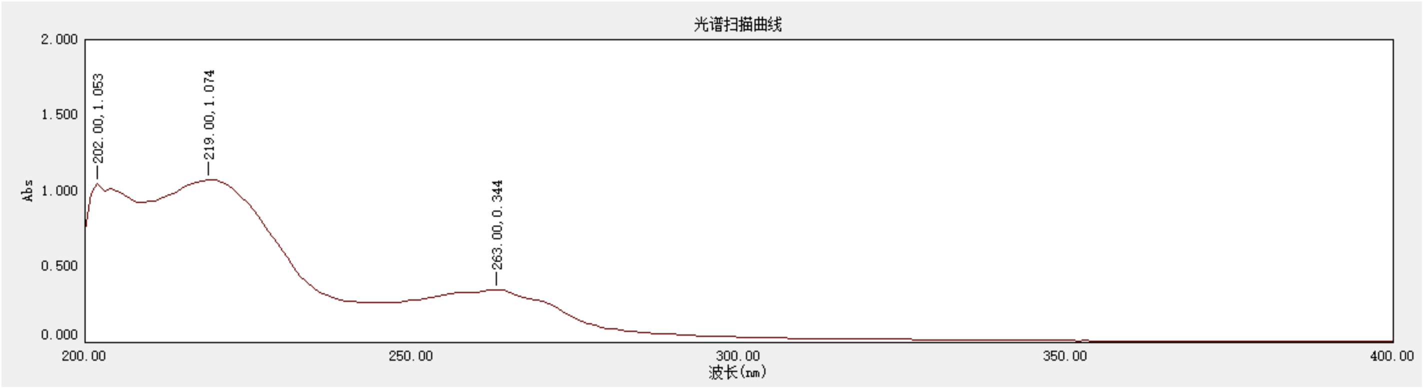

Supplementary Figure S34. CD spectrum of **4**

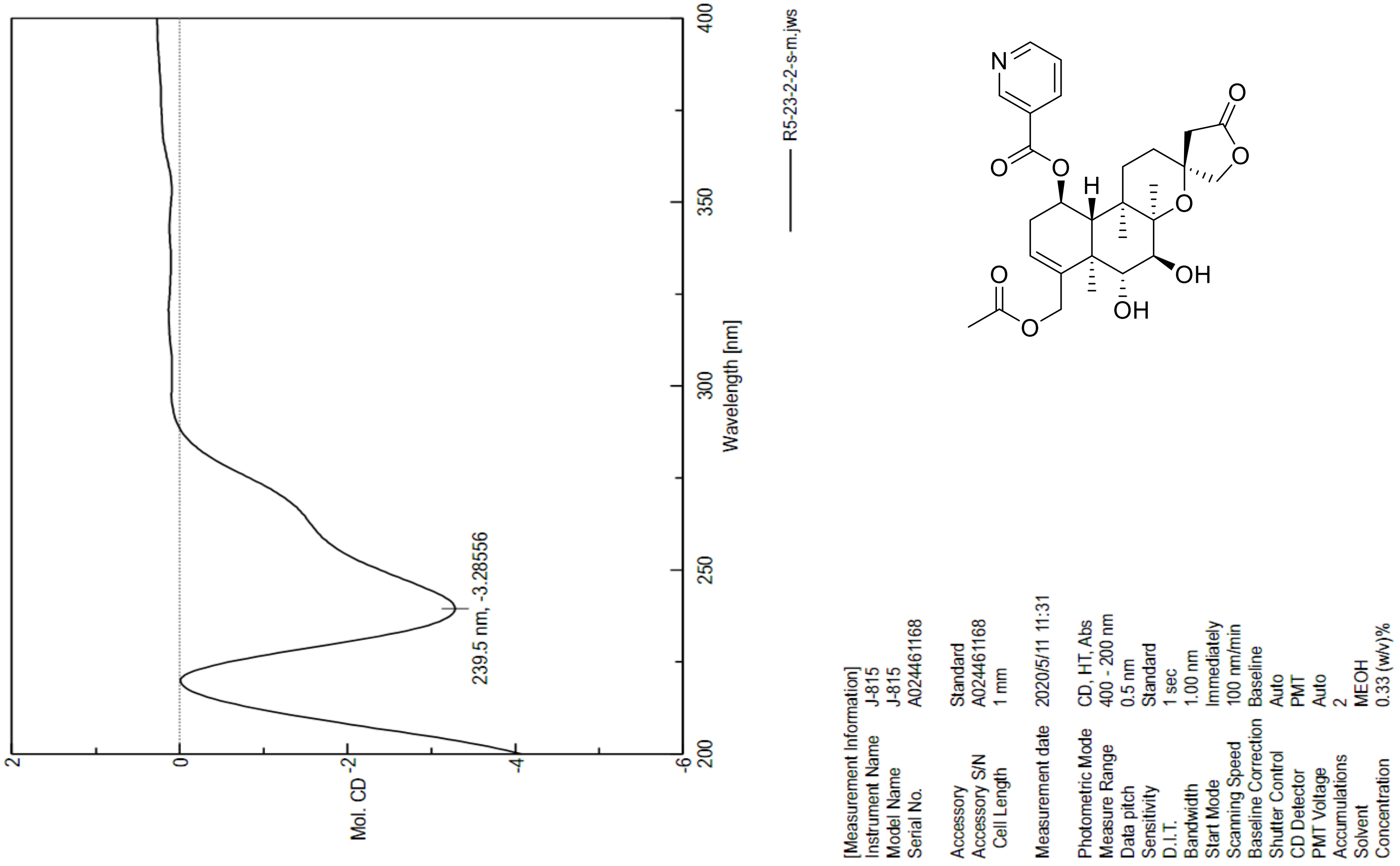

Supplementary Figure S35. ^{1}H NMR spectrum of **5**

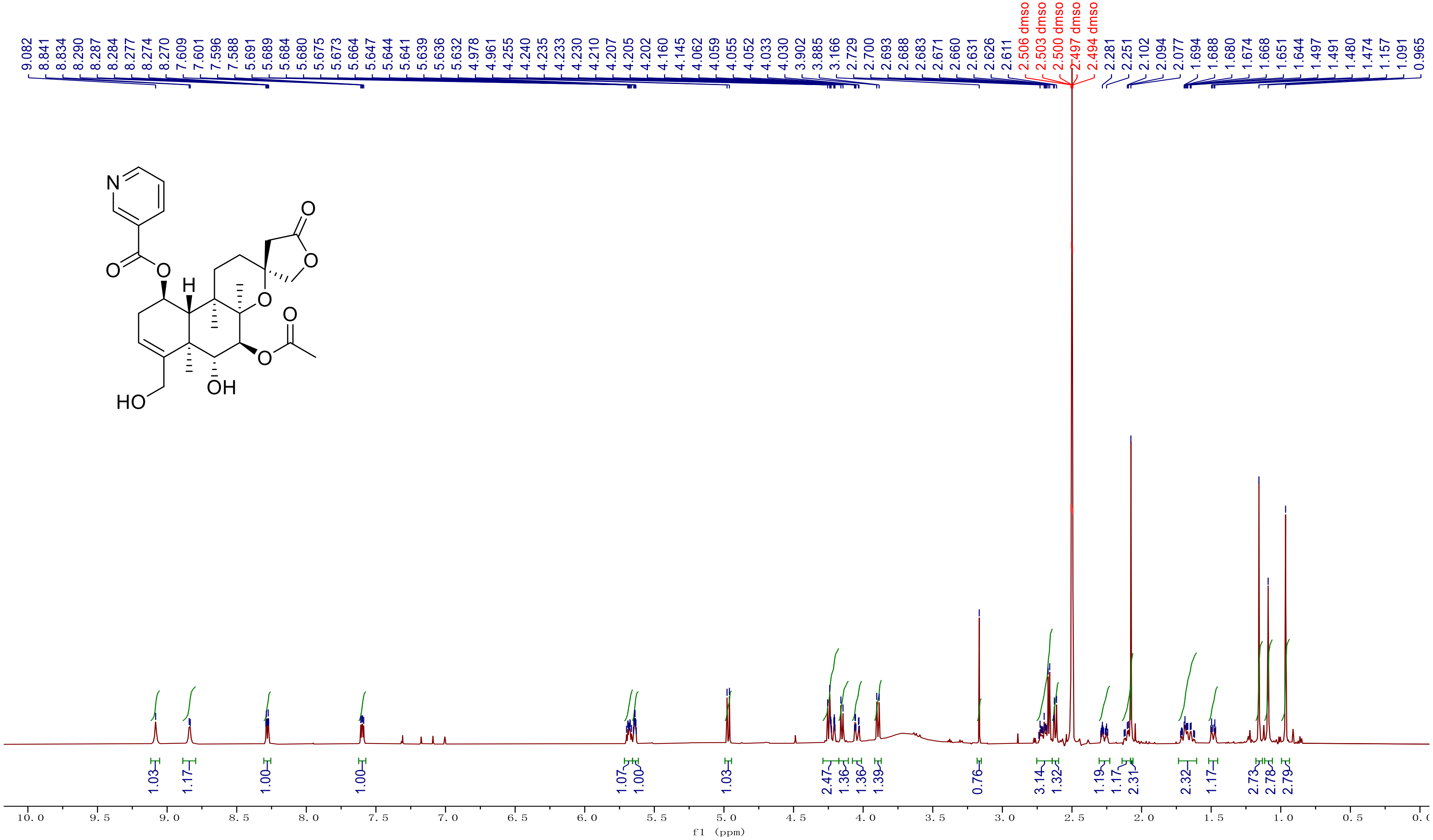

Supplementary Figure S36. ^{13}C NMR spectrum of **5**

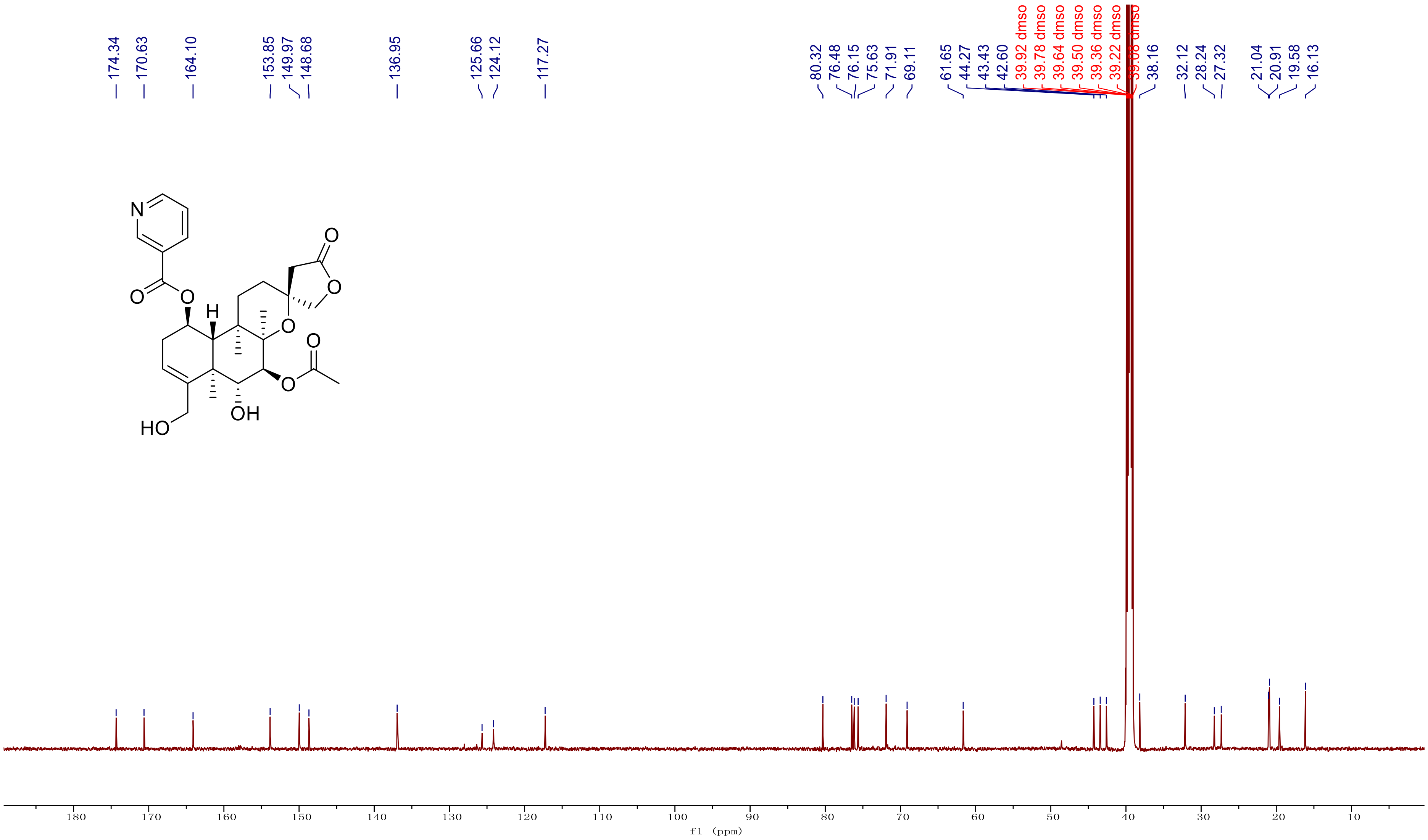

Supplementary Figure S37. DEPT spectrum of **5**

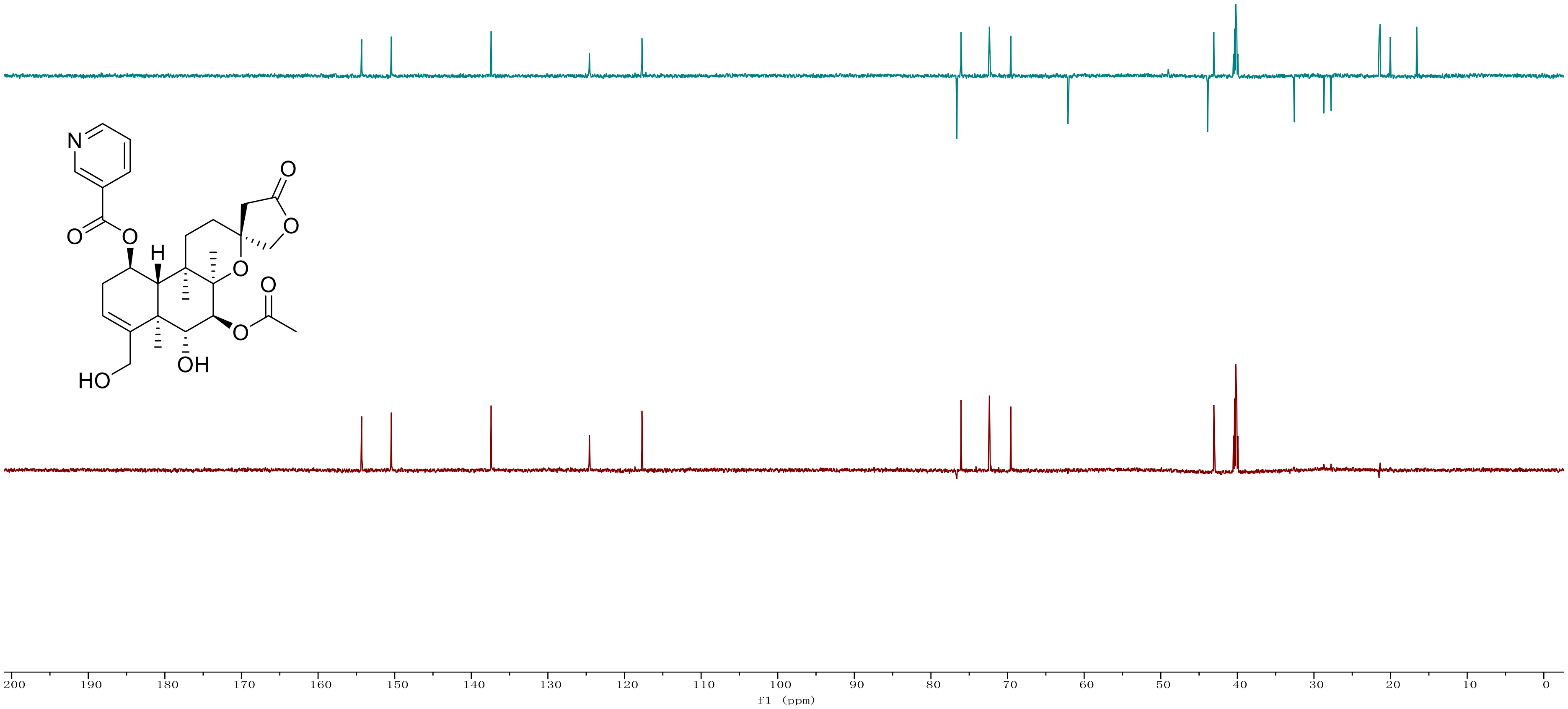

Supplementary Figure S38. ^{1}H–^{1}H COSY spectrum of **5**

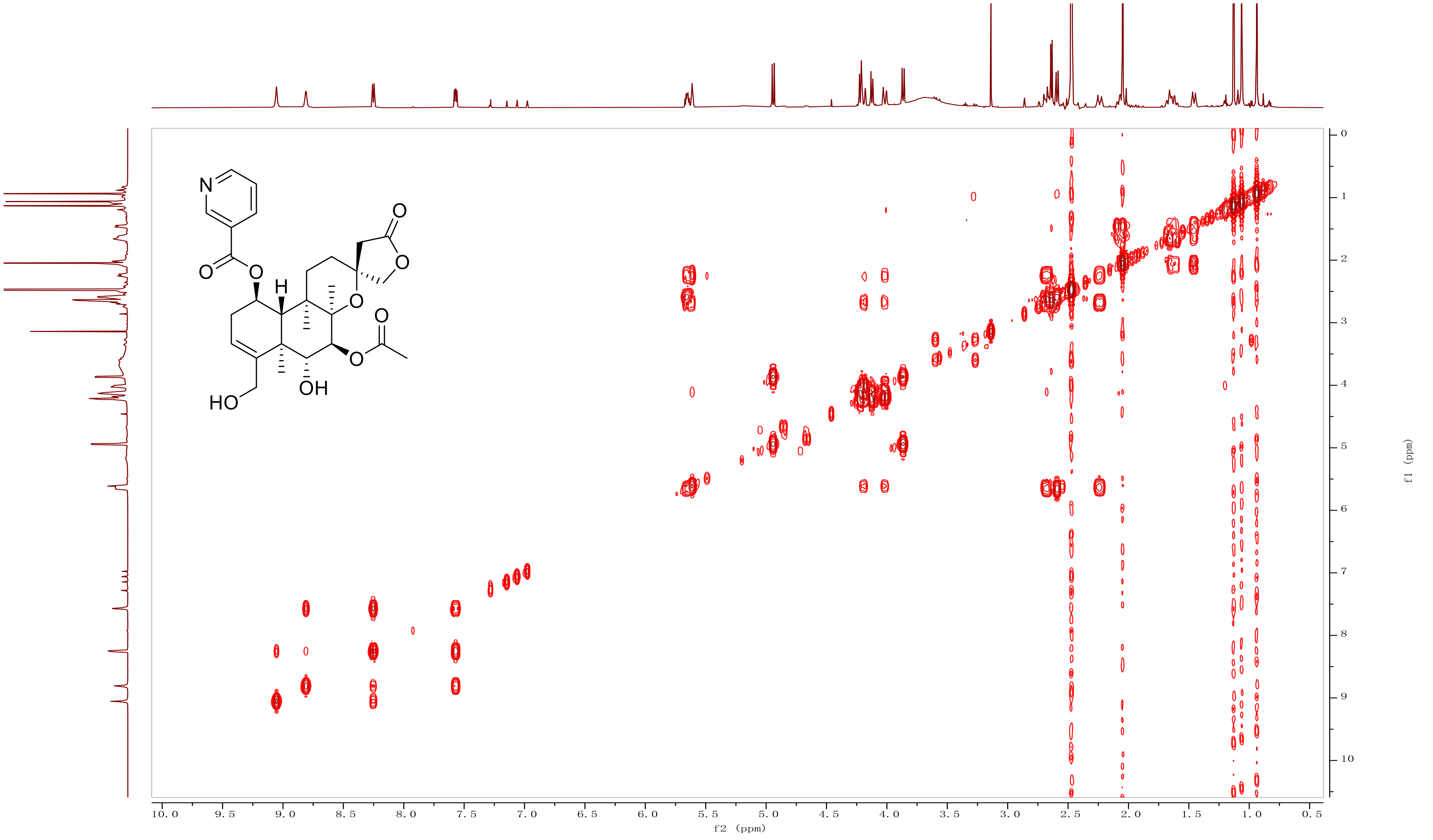

Supplementary Figure S39. HSQC spectrum of **5**

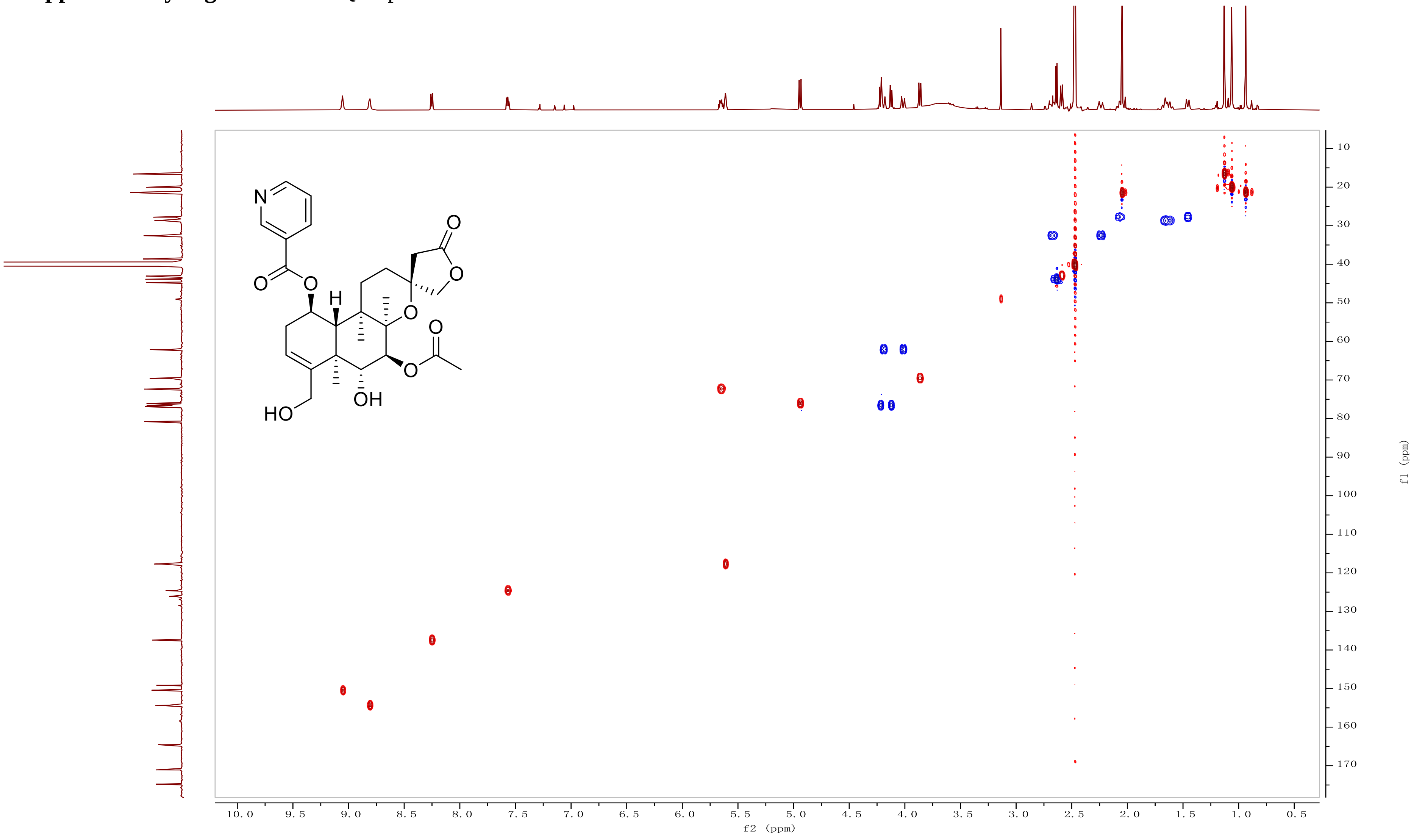

Supplementary Figure S40. HMBC spectrum of **5**

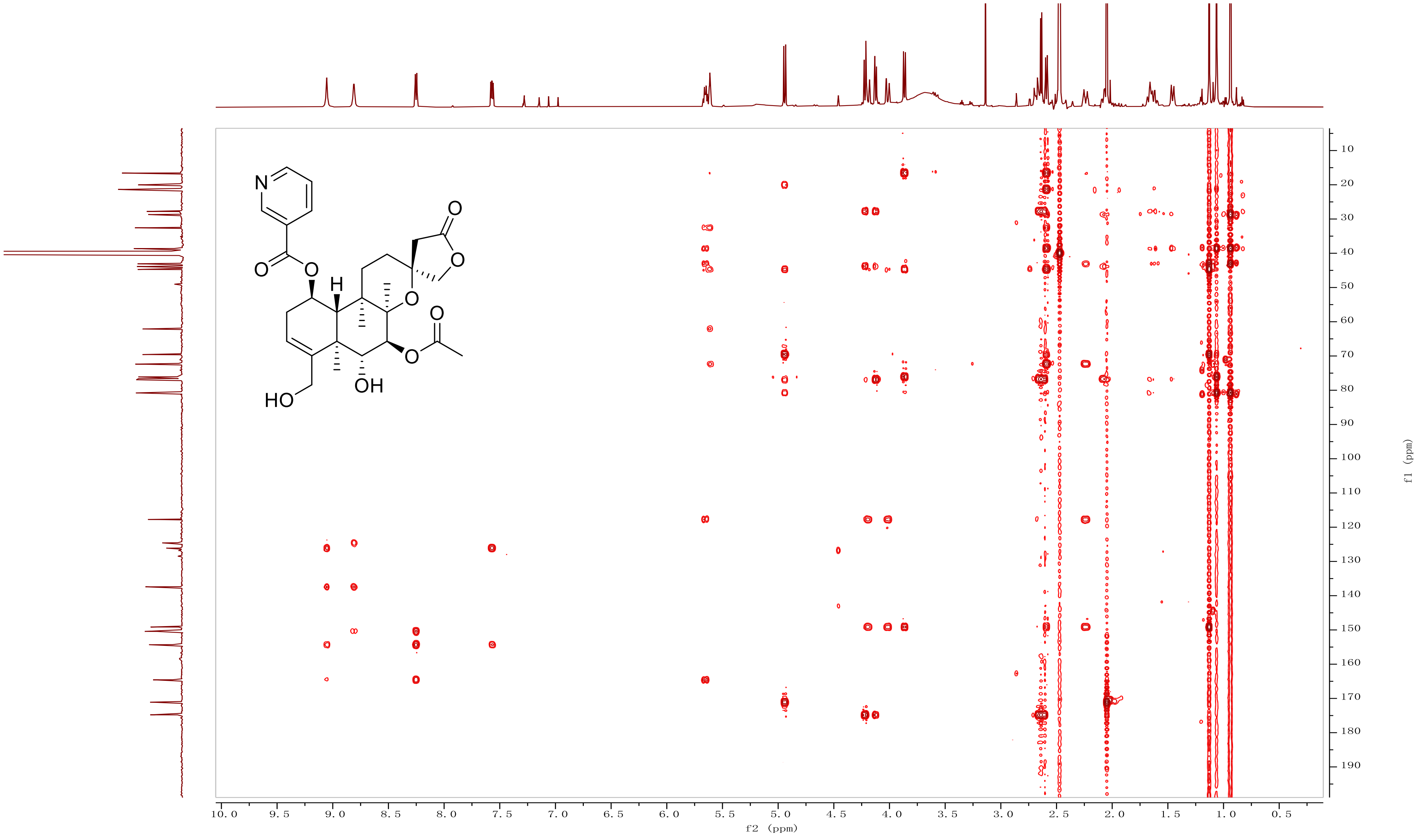

Supplementary Figure S41. 1D NOE spectrum of **5**

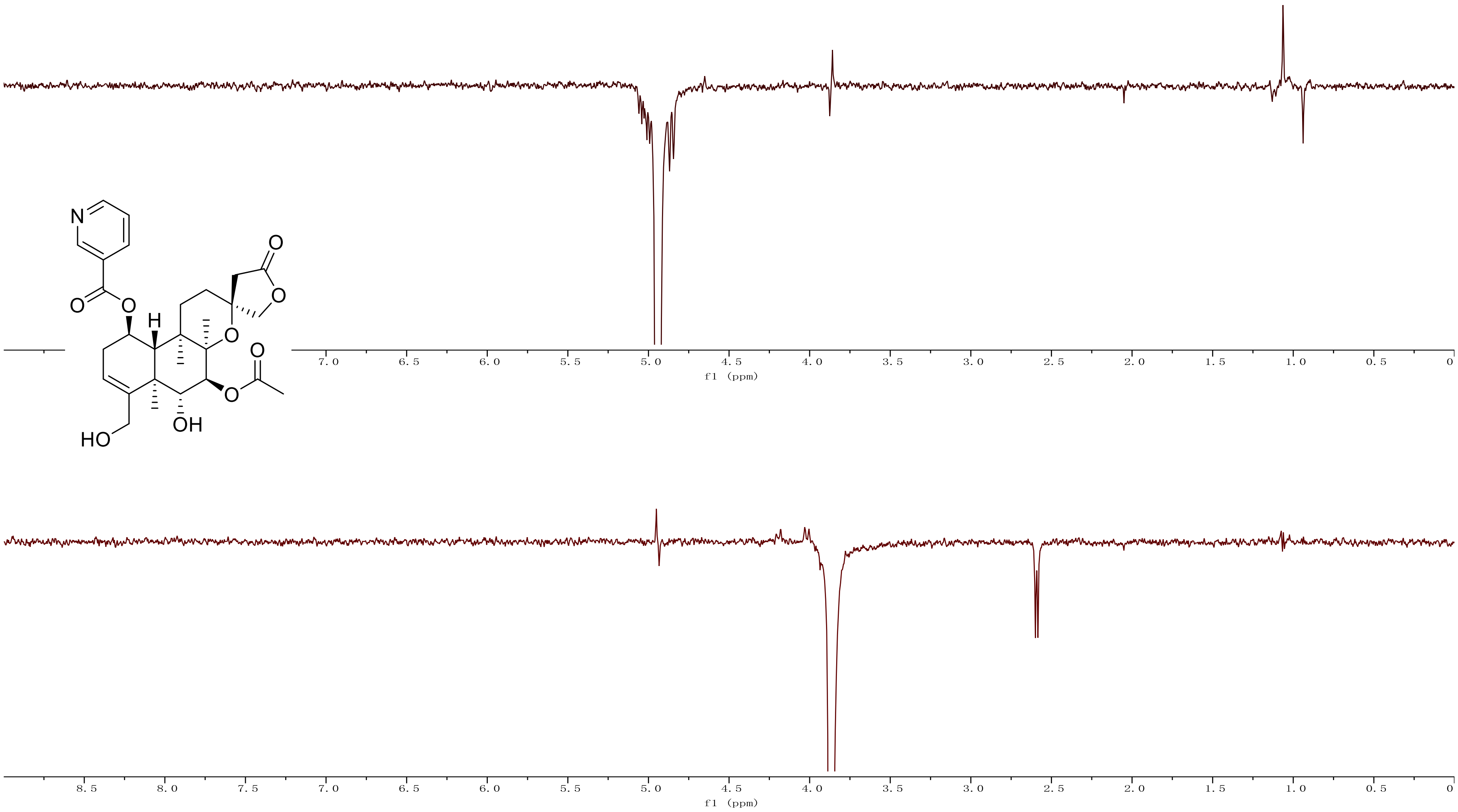

Supplementary Figure S42. HRESIMS spectrum of **5**

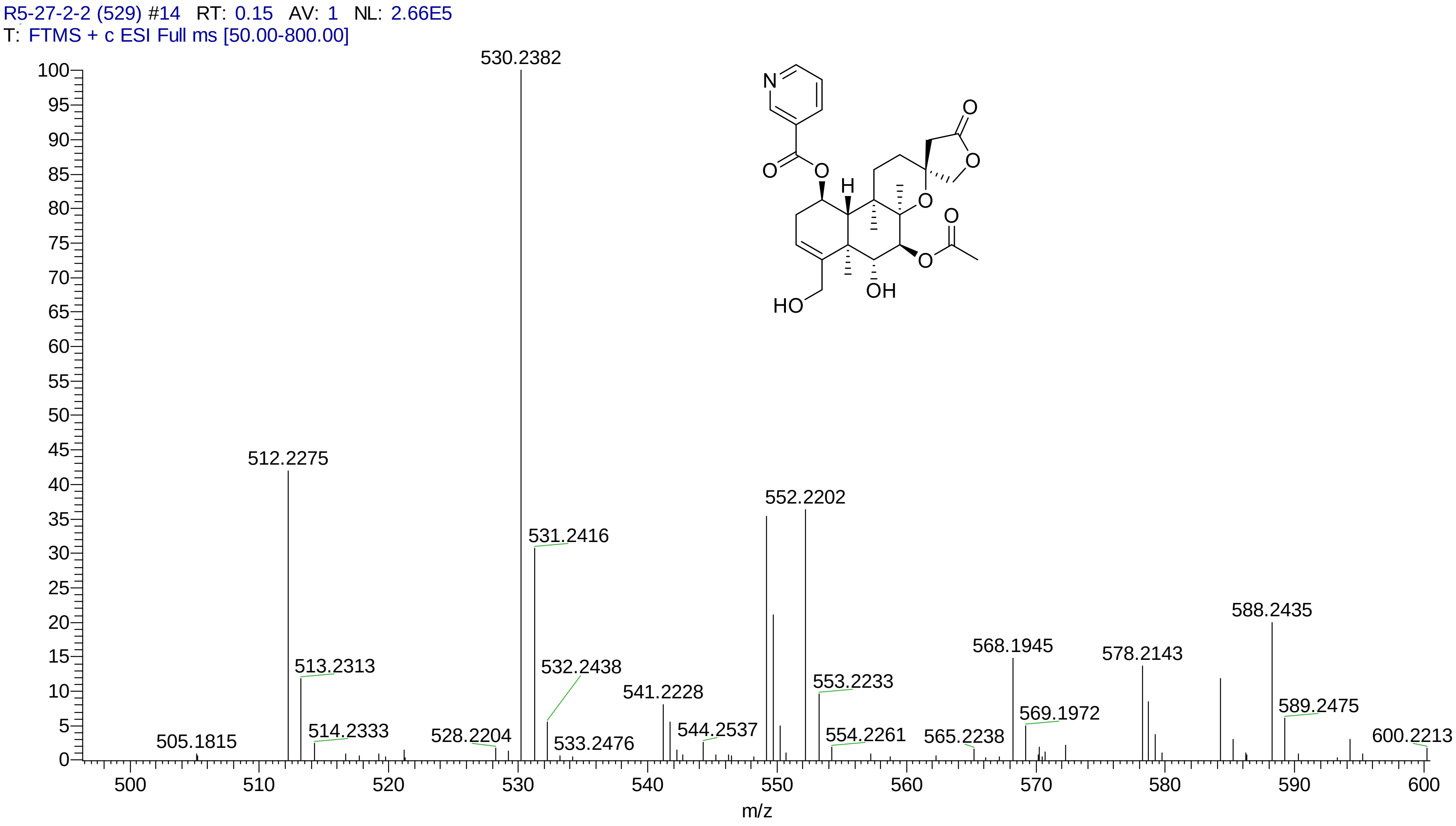

Supplementary Figure S43. IR spectrum of **5**

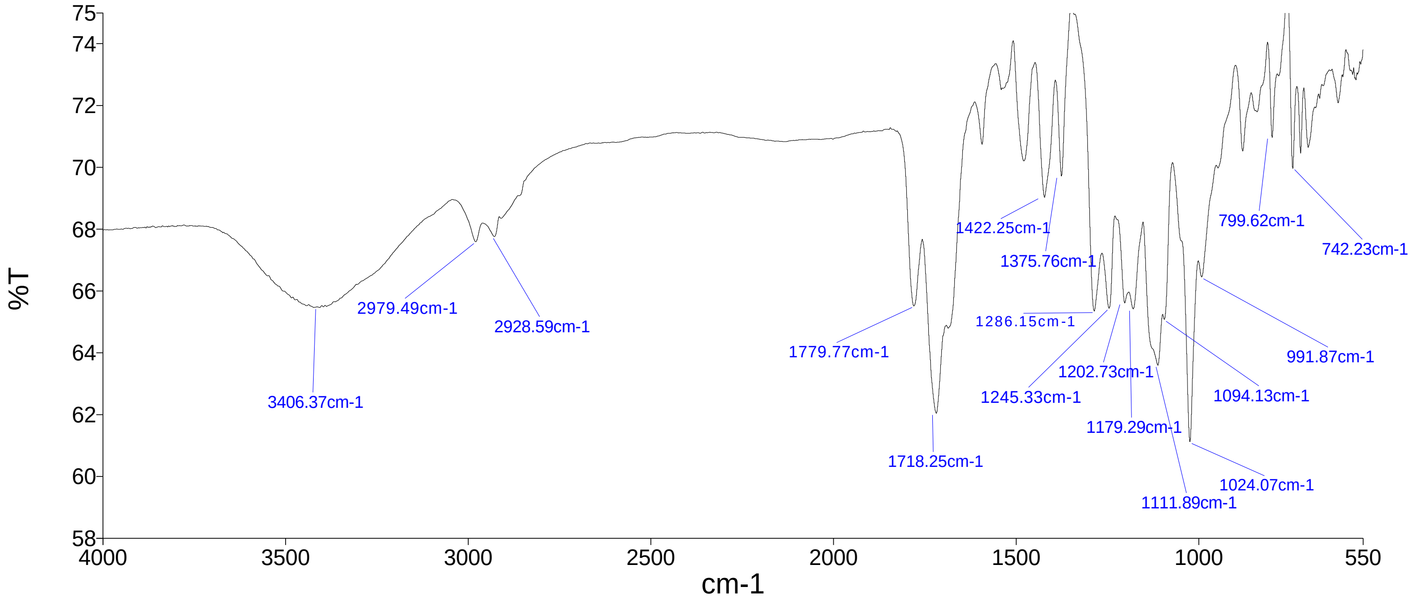

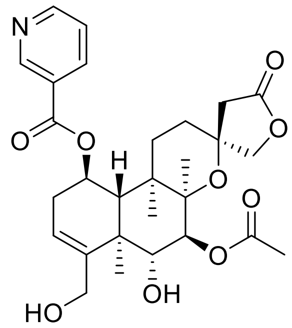

Supplementary Figure S44. UV spectrum of **5**

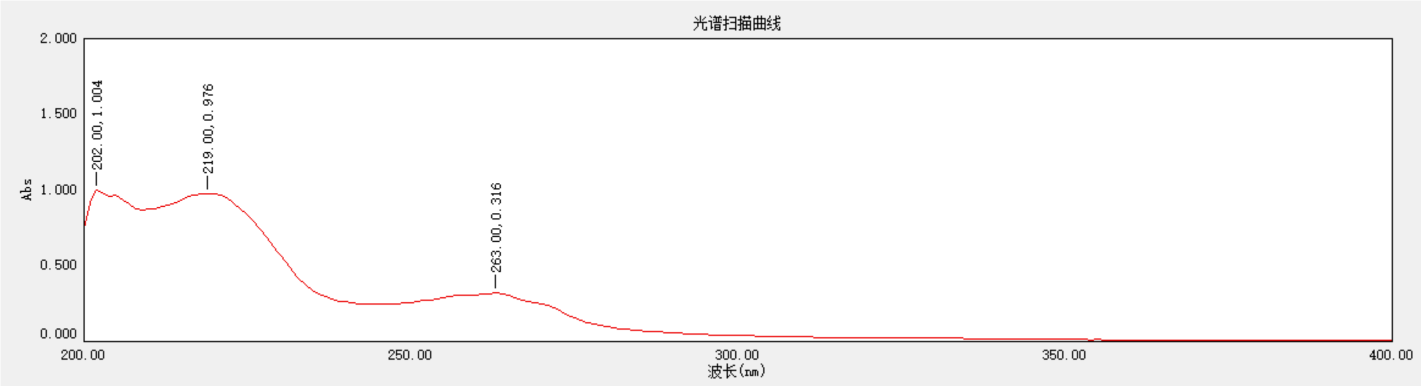

Supplementary Figure S45. CD spectrum of **5**

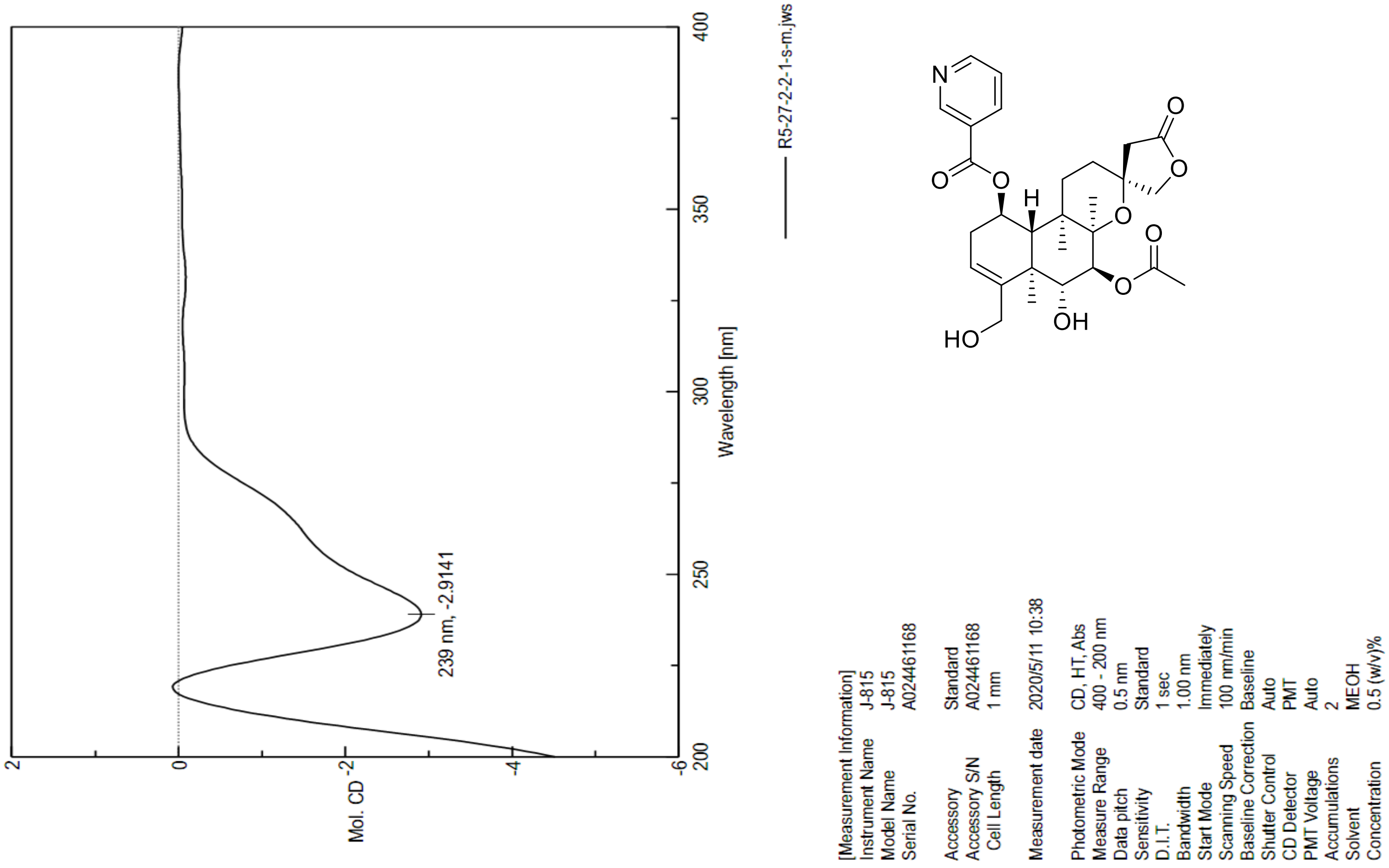

Supplementary Figure S46. ^{1}H NMR spectrum of **6**

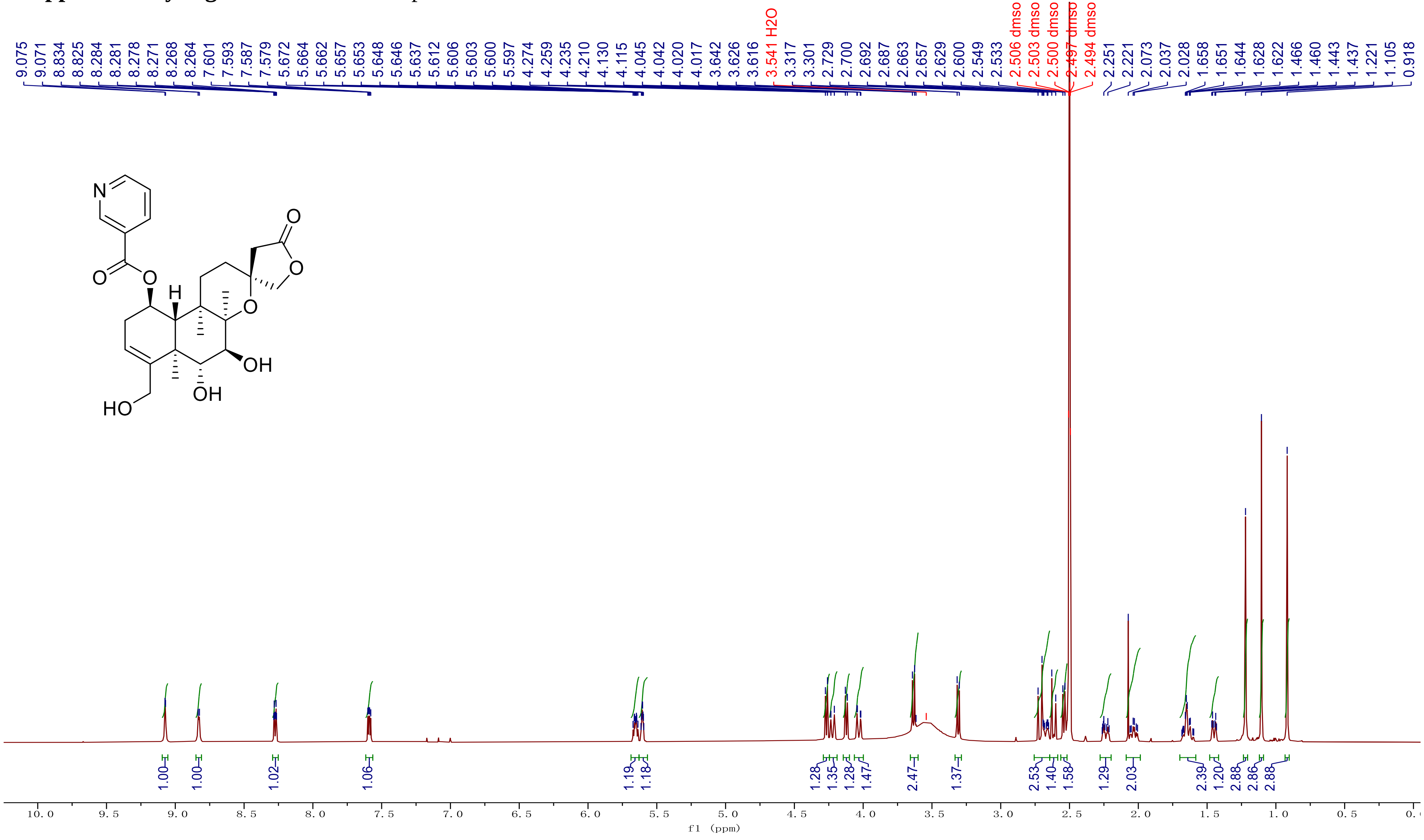

Supplementary Figure S47. ^{13}C NMR spectrum of **6**

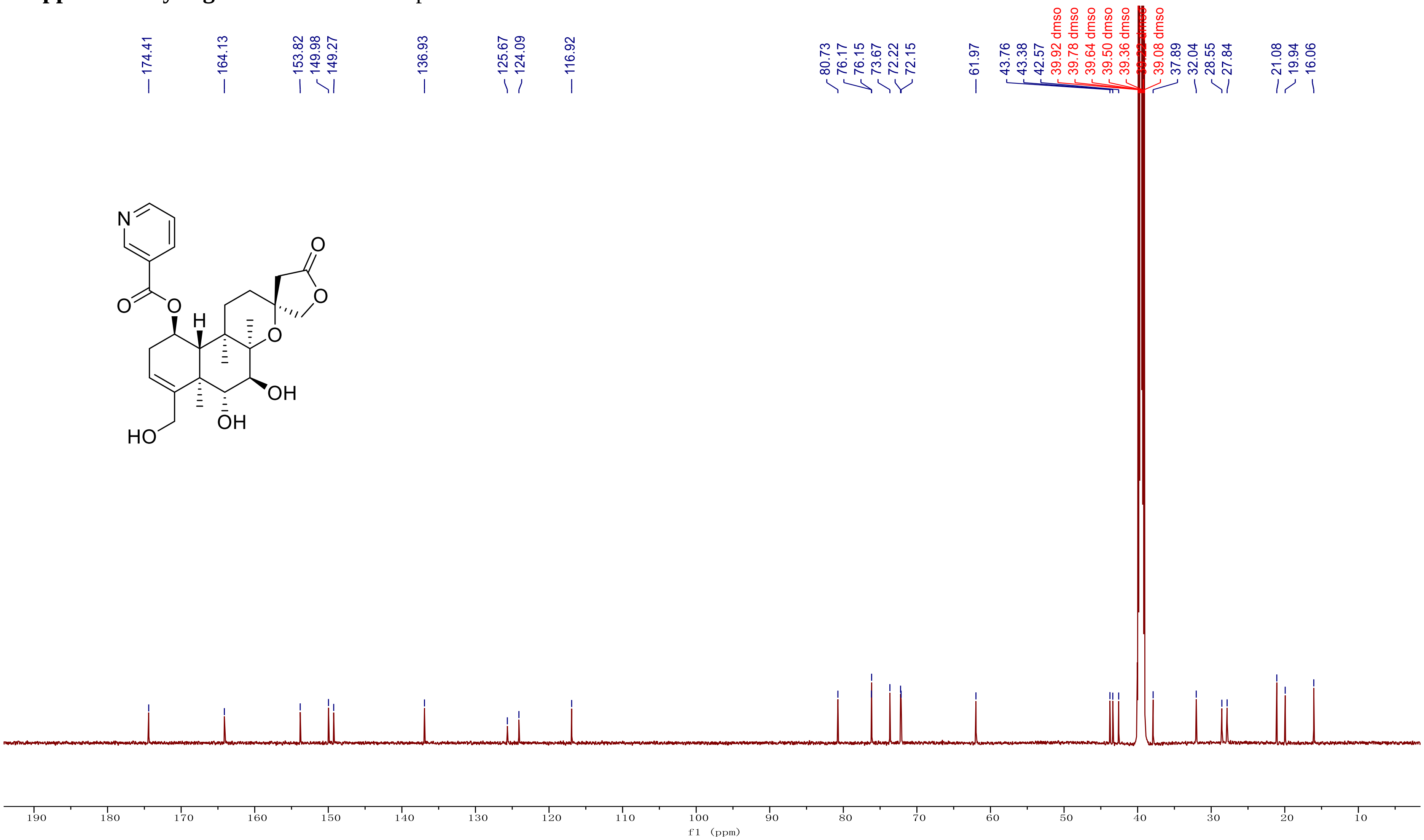

Supplementary Figure S48. DEPT spectrum of **6**

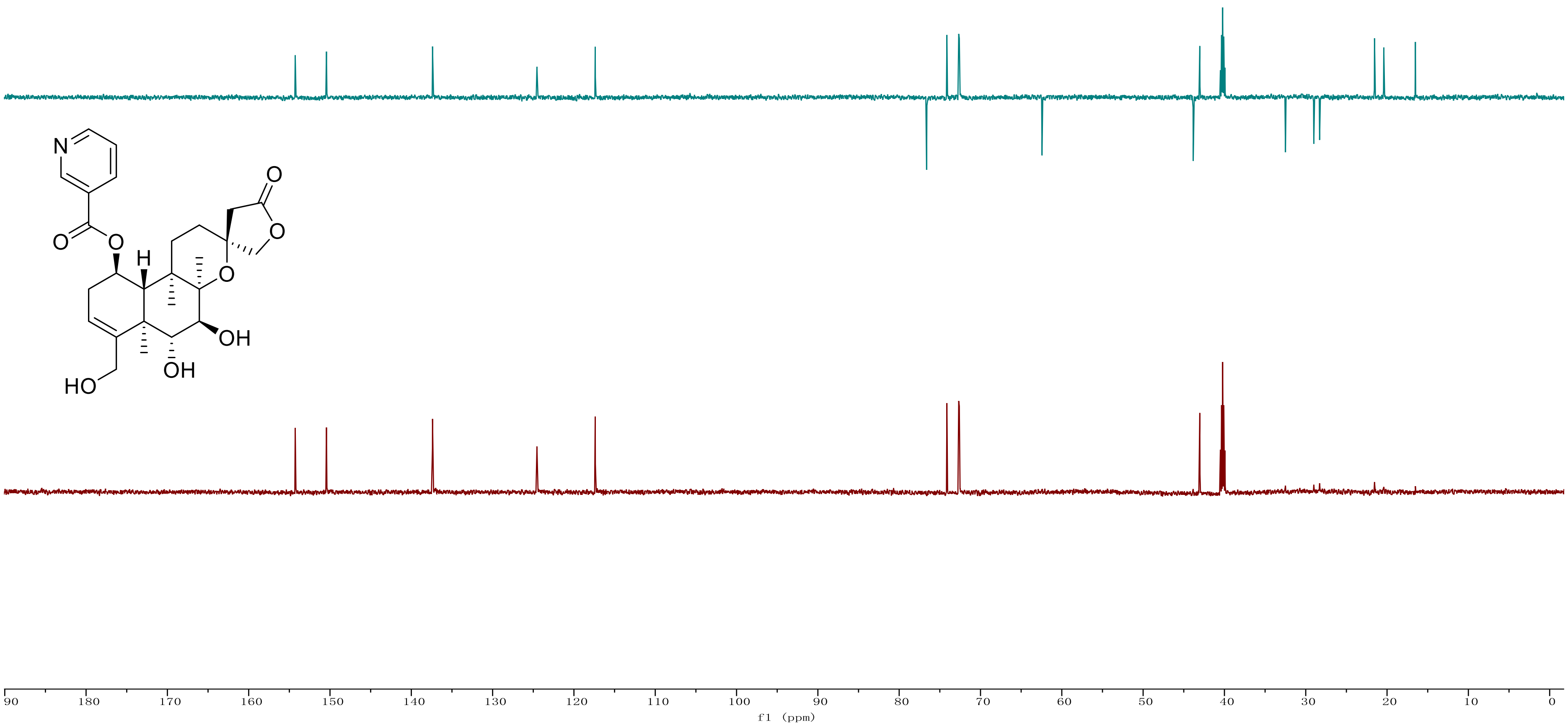

Supplementary Figure S49. ^{1}H–^{1}H COSY spectrum of **6**

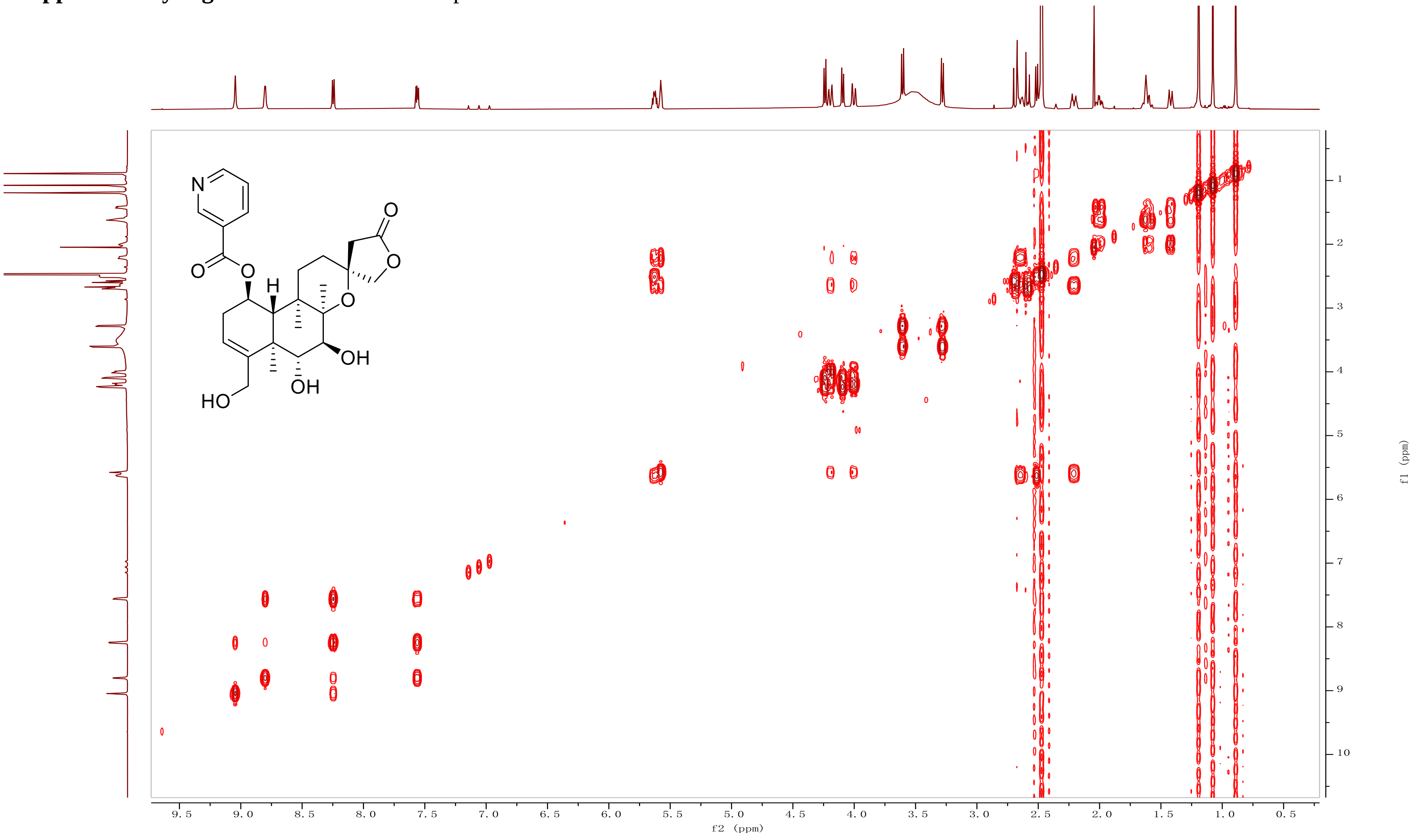

Supplementary Figure S50. HSQC spectrum of **6**

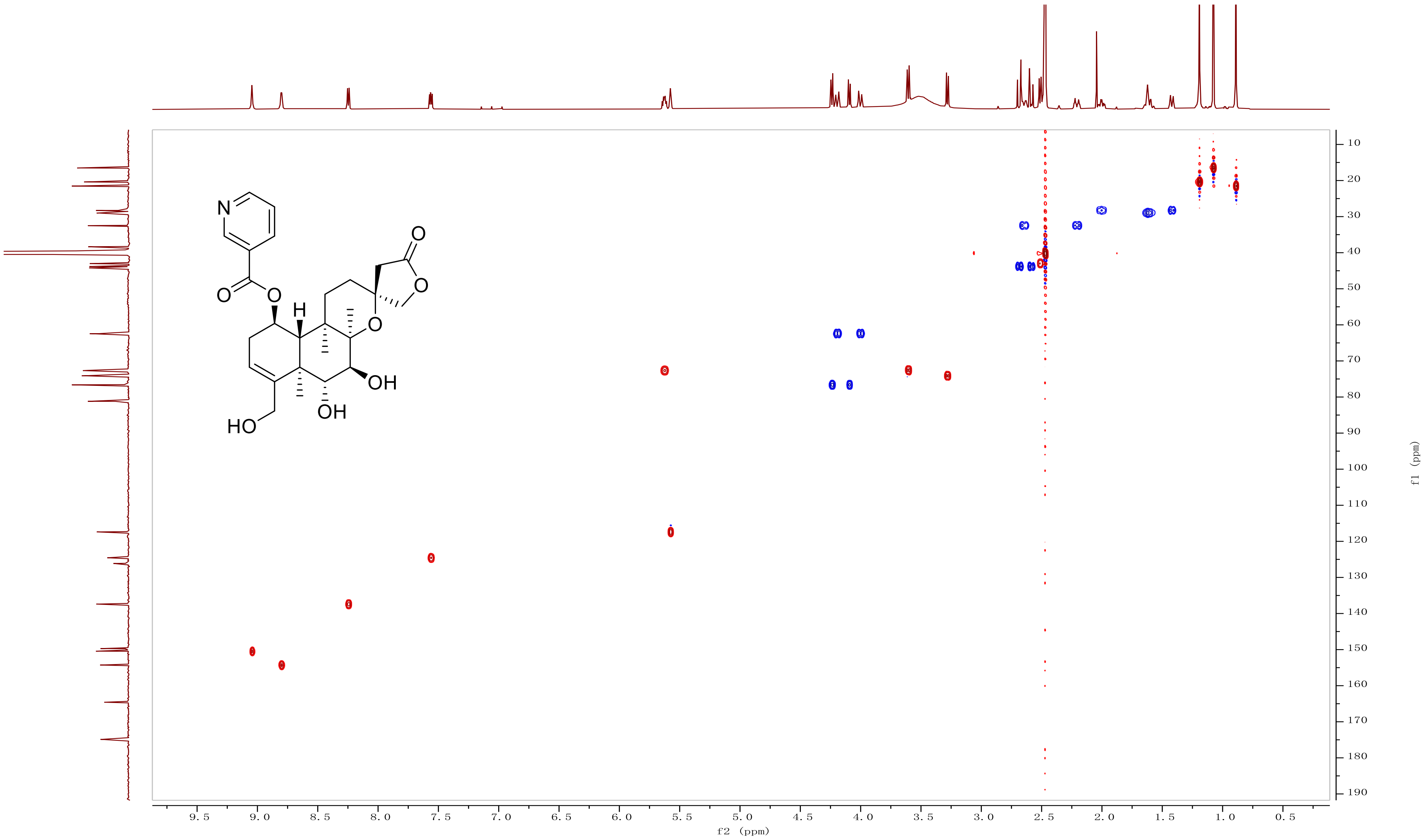

Supplementary Figure S51. HMBC spectrum of **6**

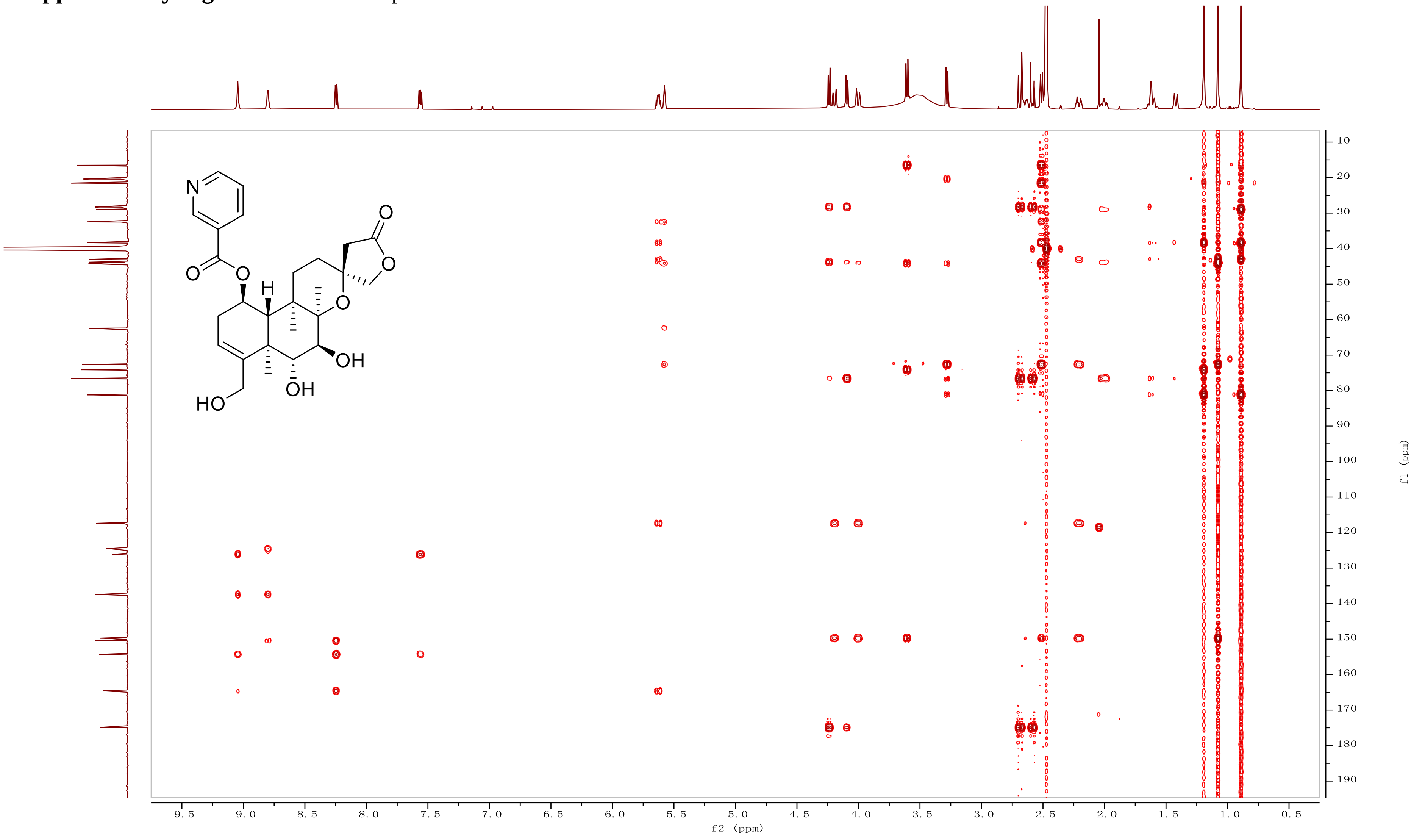

Supplementary Figure S52. 1D NOE spectrum of **6**

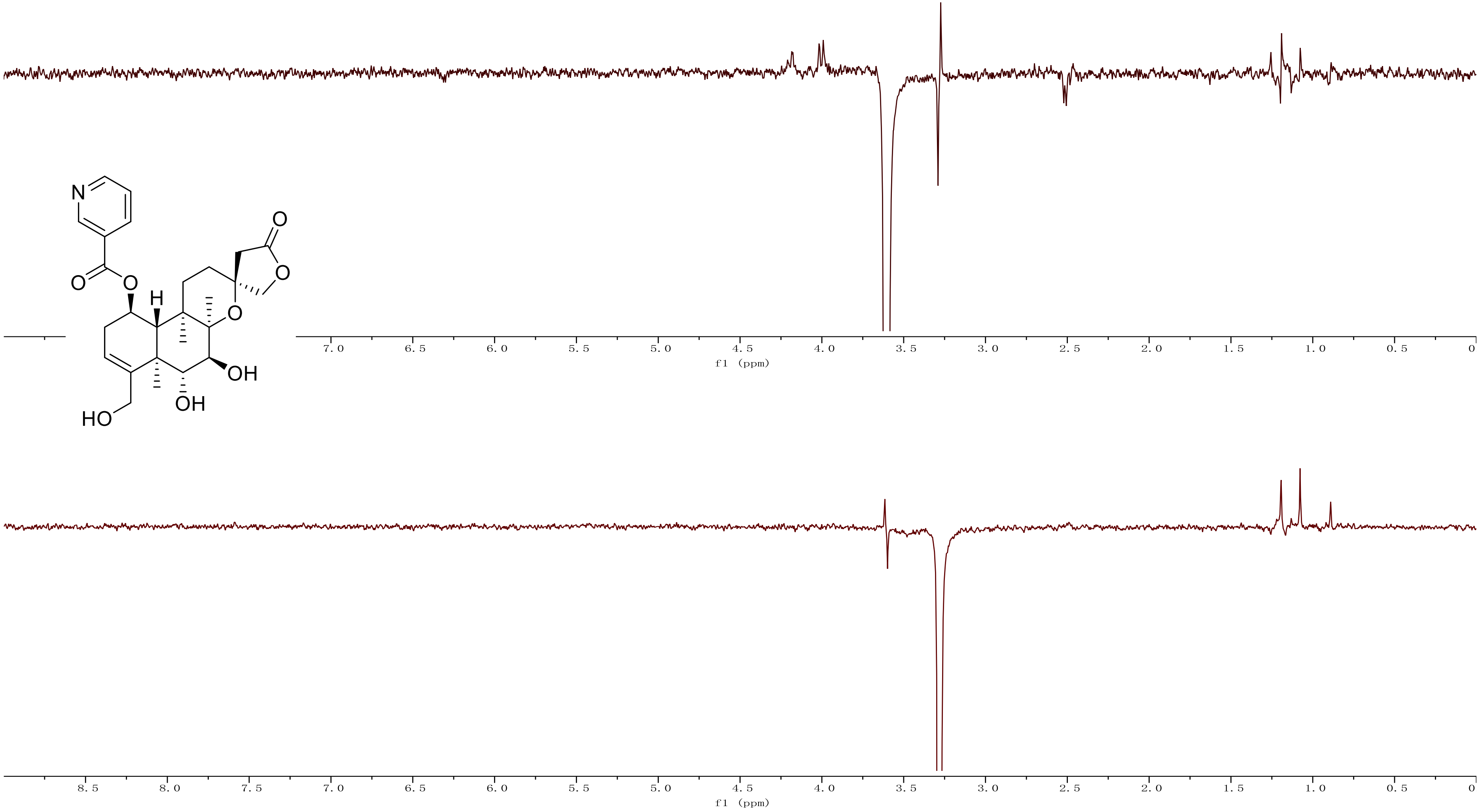

Supplementary Figure S53. HRESIMS spectrum of **6**

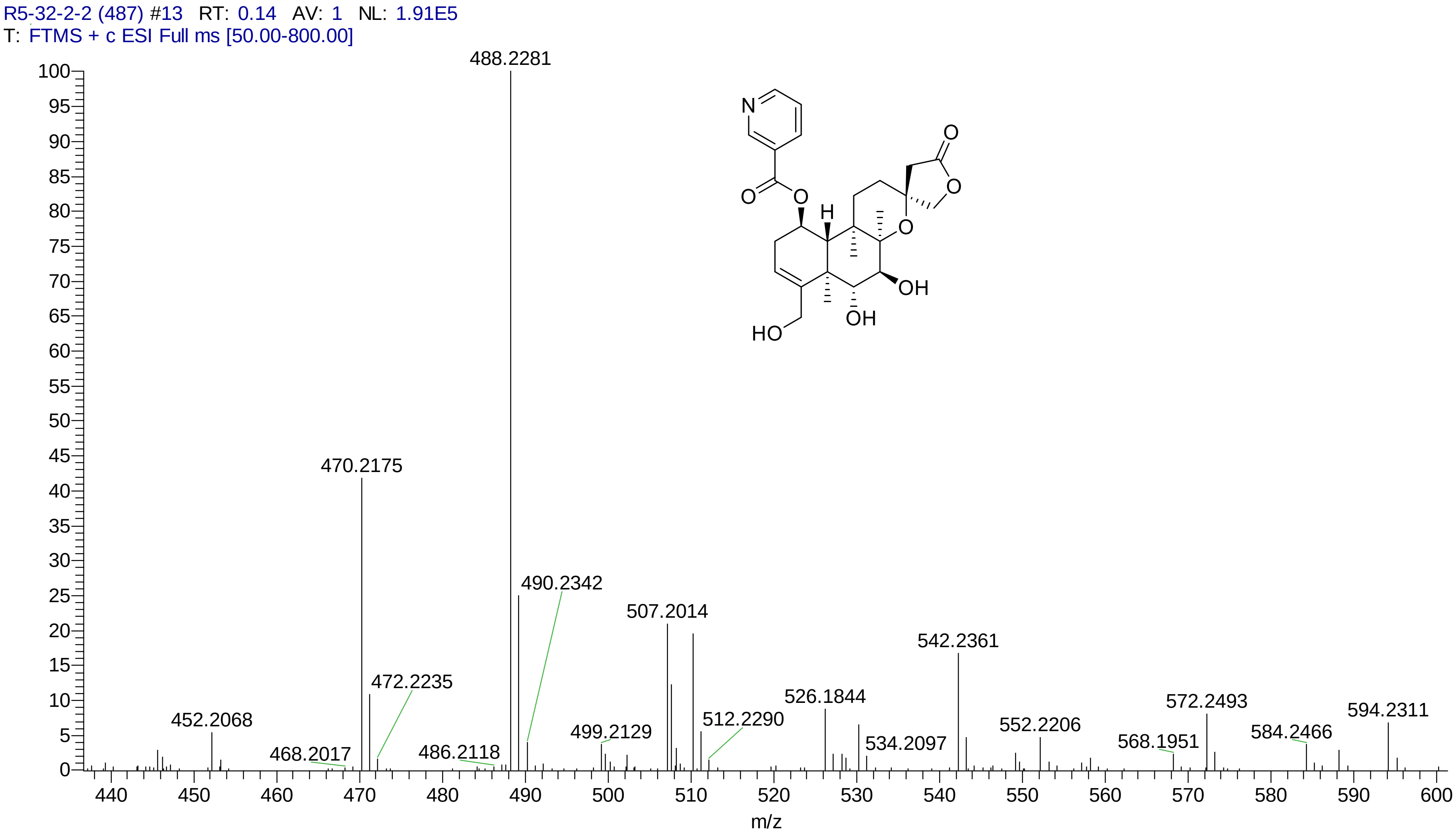

Supplementary Figure S54. IR spectrum of **6**

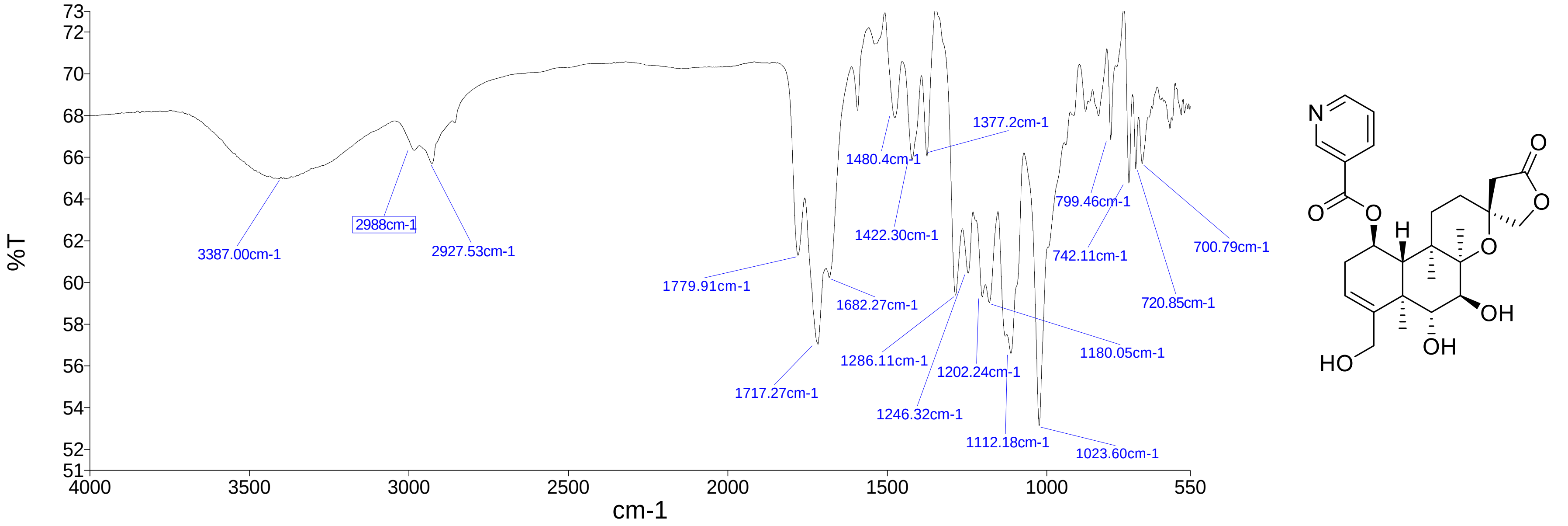

Supplementary Figure S55. UV spectrum of **6**

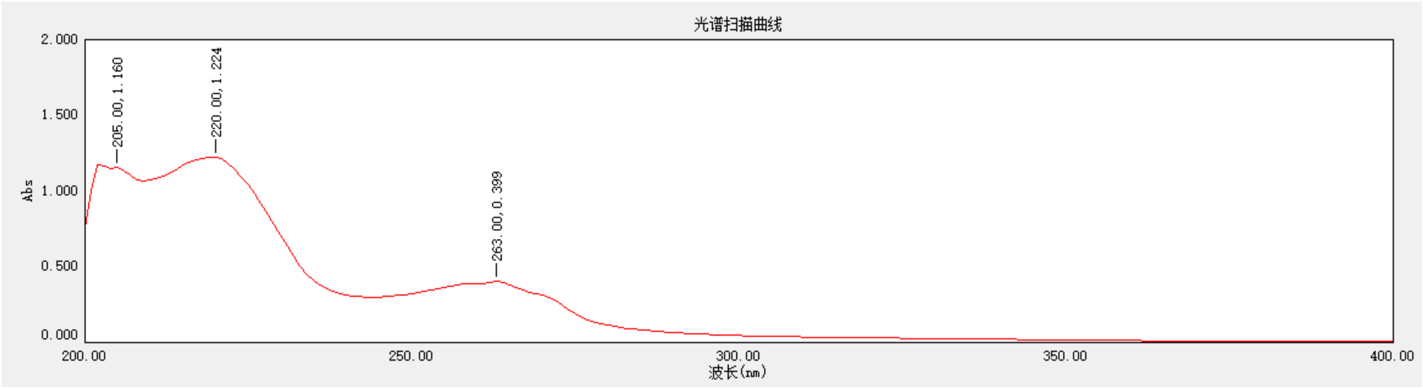

Supplementary Figure S56. CD spectrum of **6**

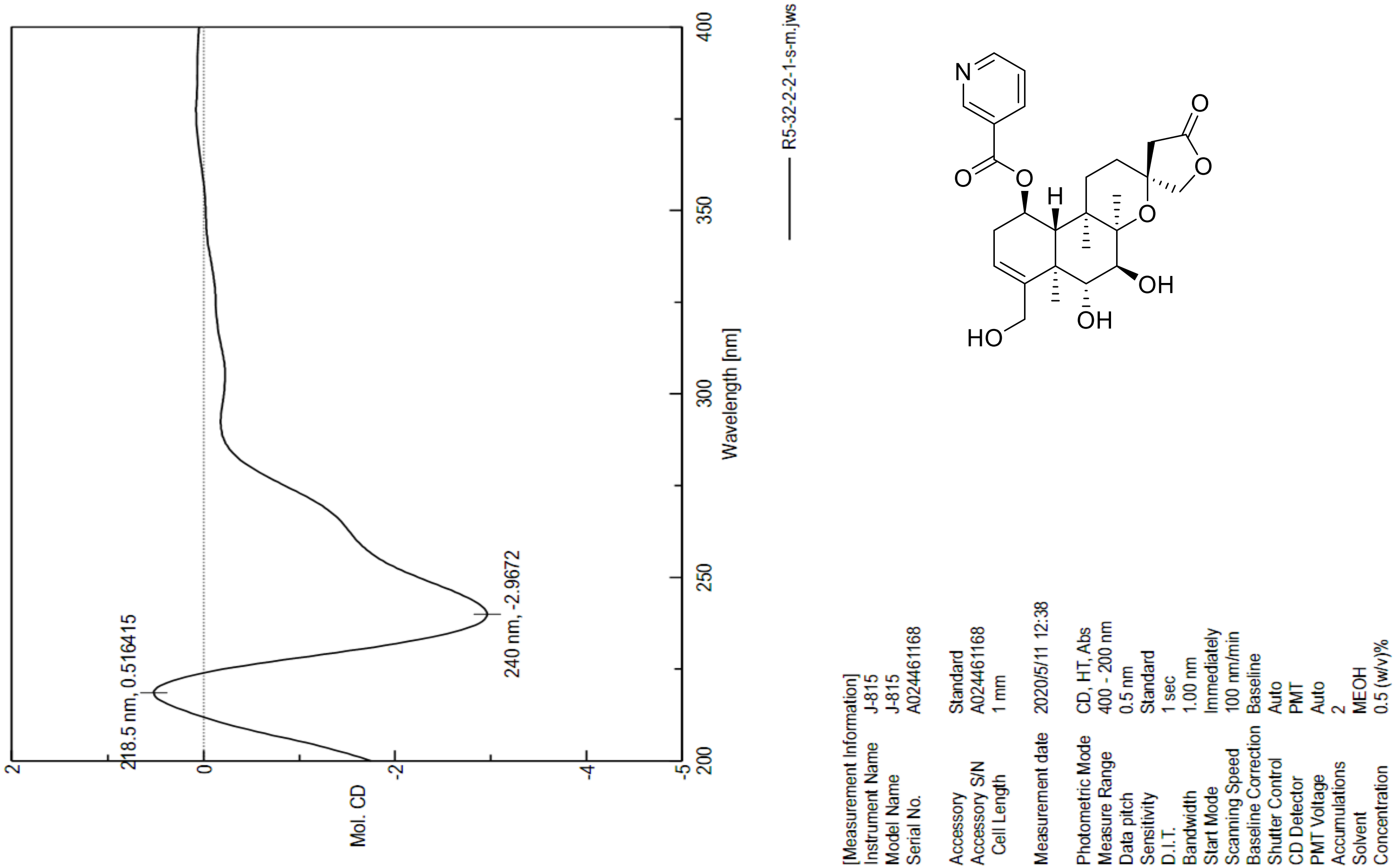

Supplementary Figure S57. ^{1}H NMR spectrum of **7**

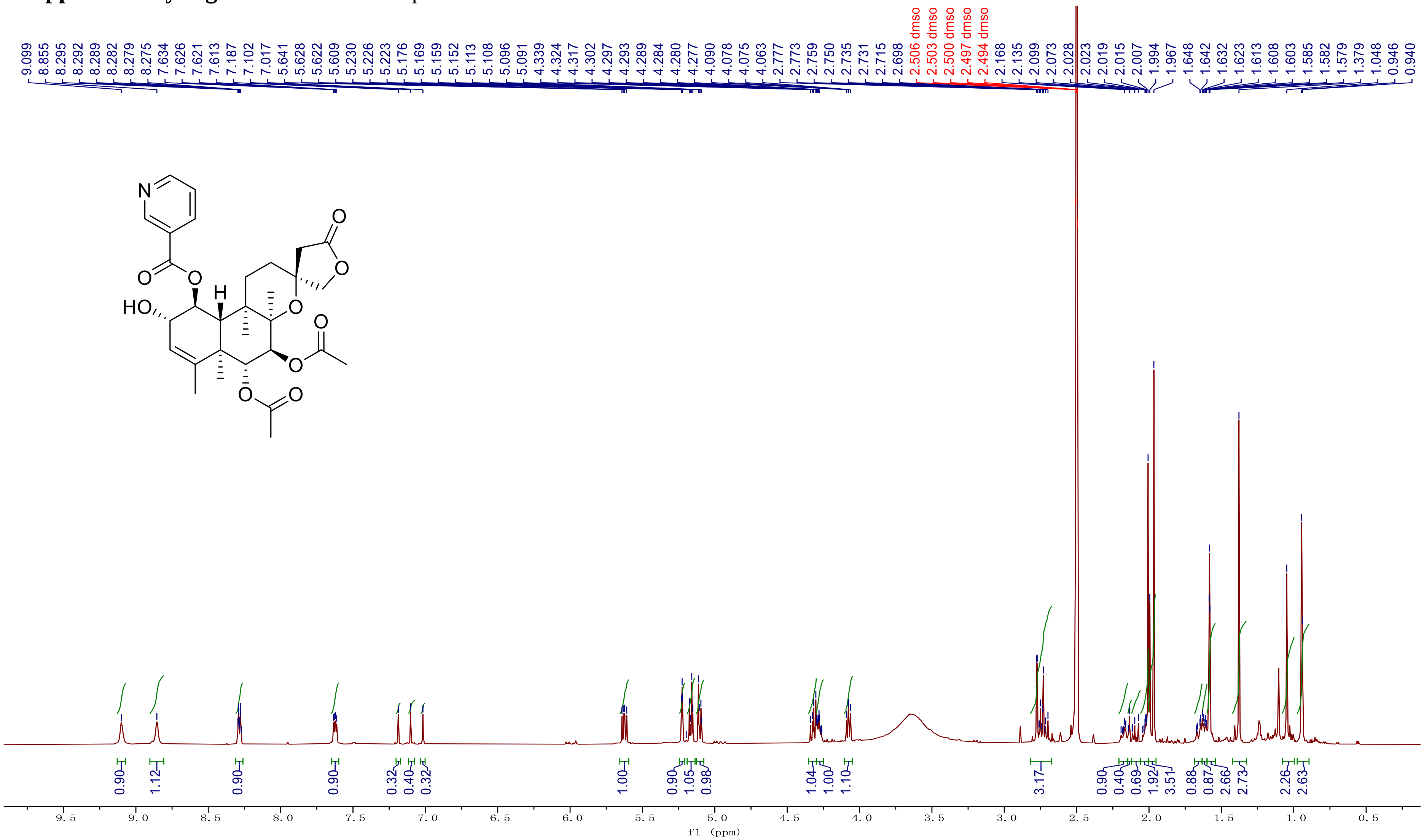

Supplementary Figure S58. ^{13}C NMR spectrum of **7**

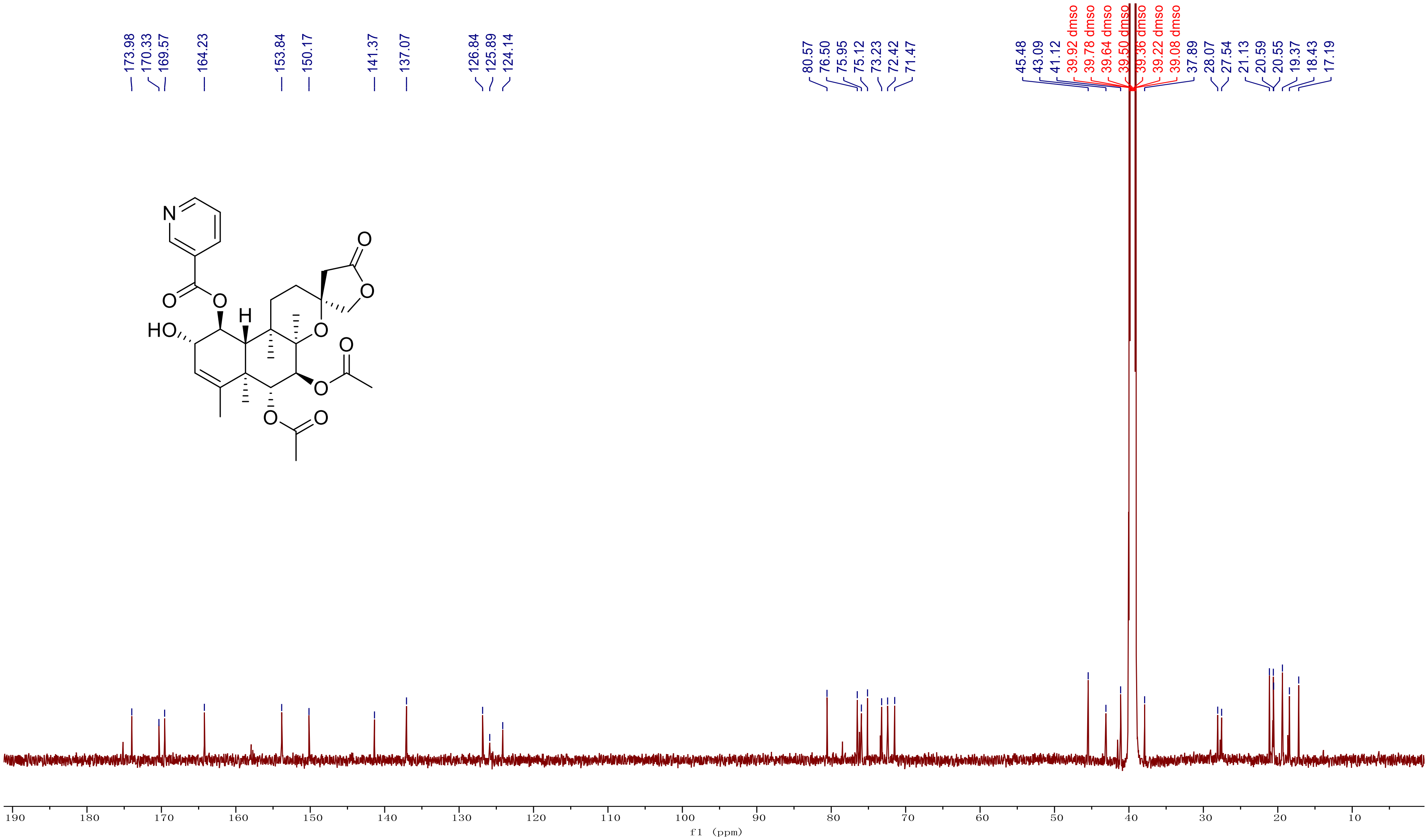

Supplementary Figure S59. DEPT spectrum of **7**

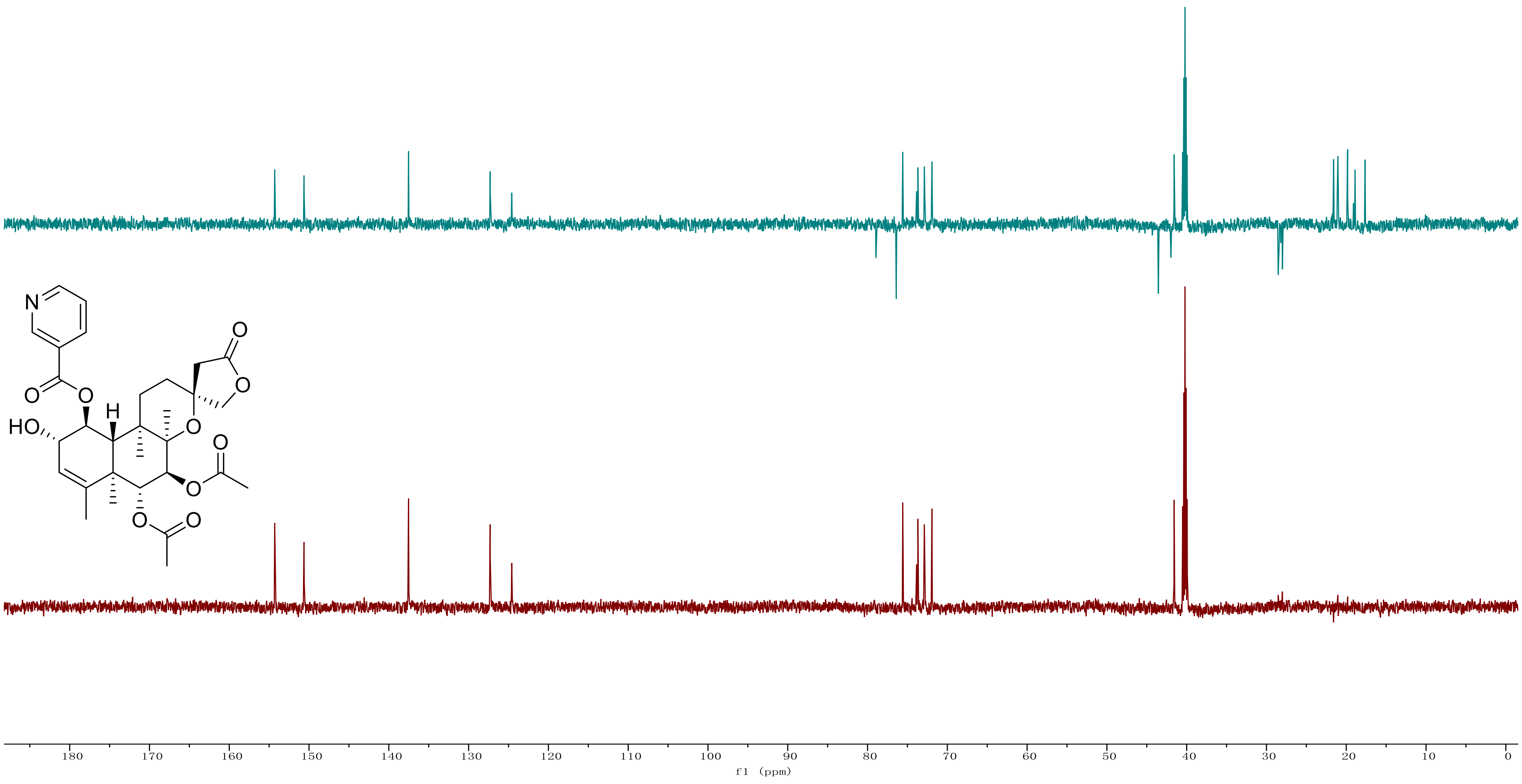

Supplementary Figure S60. ^{1}H–^{1}H COSY spectrum of **7**

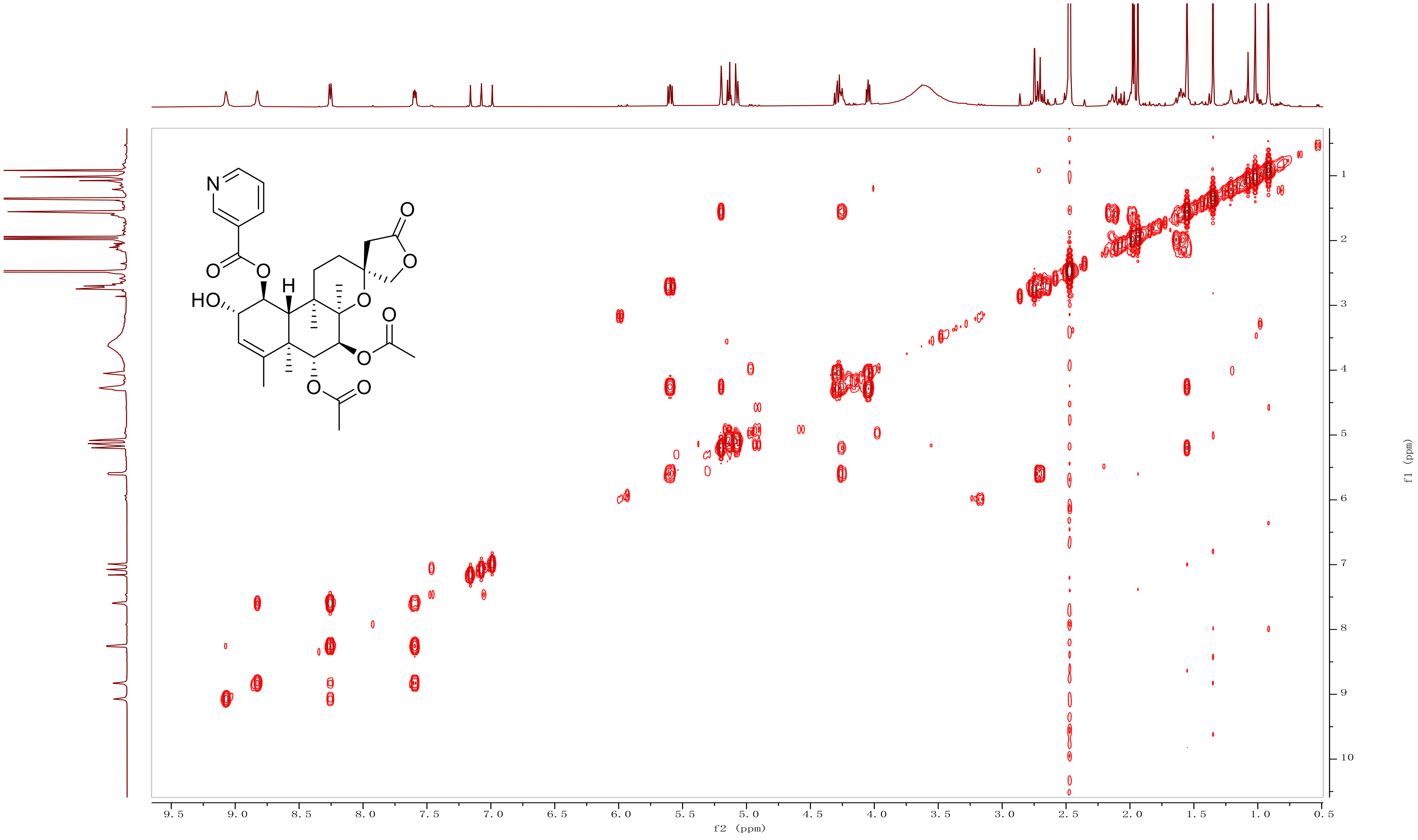

Supplementary Figure S61. HSQC spectrum of **7**

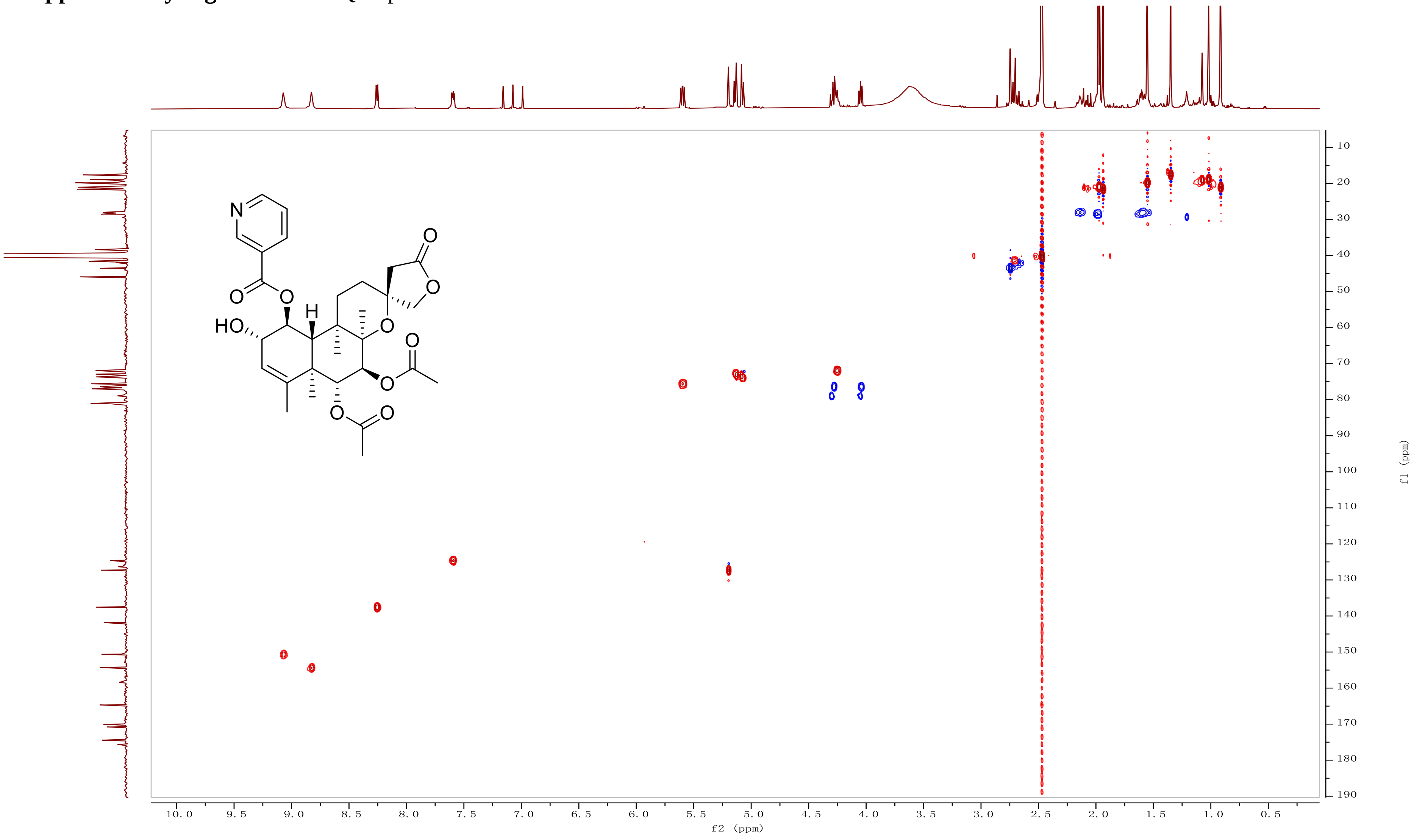

Supplementary Figure S62. HMBC spectrum of **7**

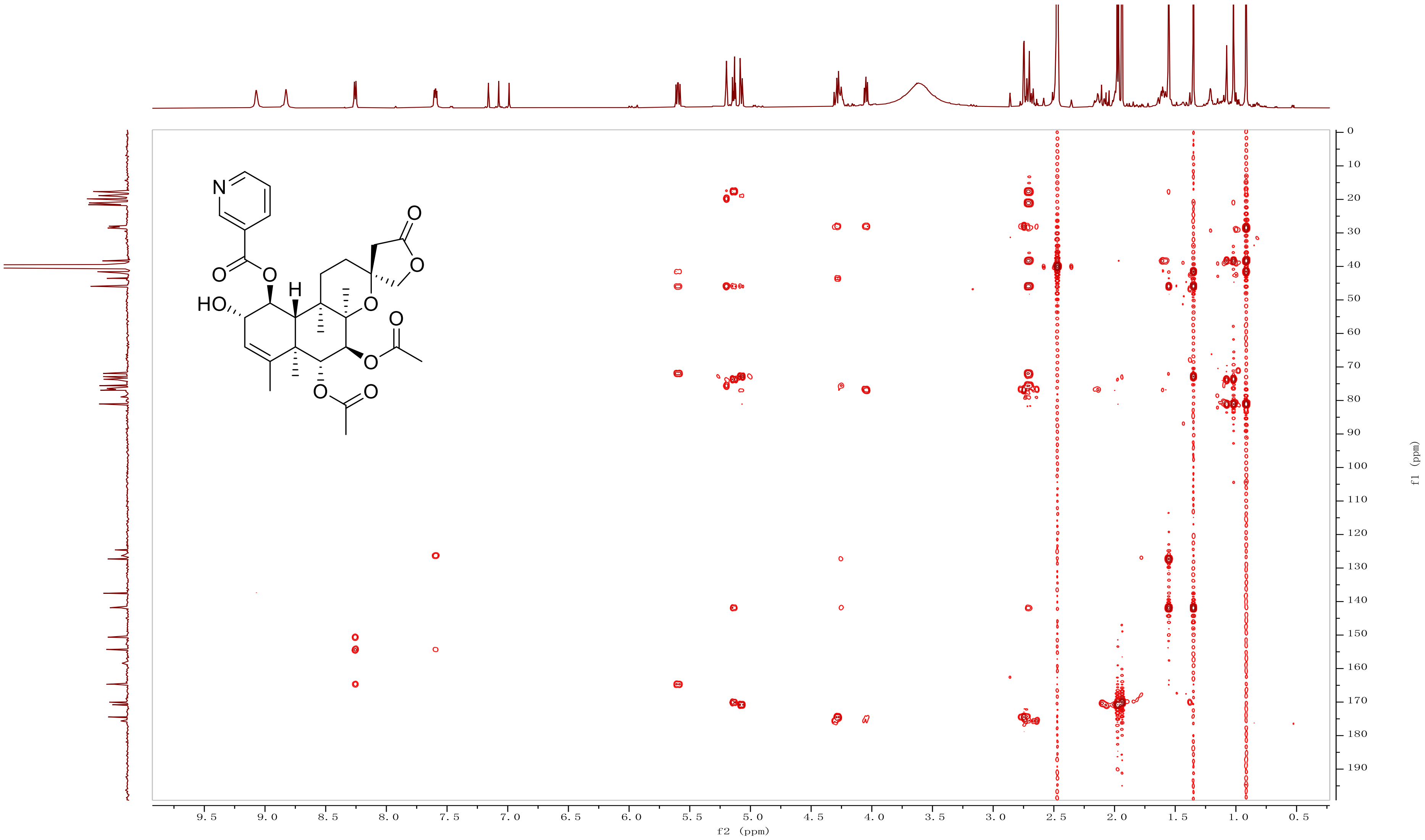

Supplementary Figure S63. NOESY spectrum of **7**

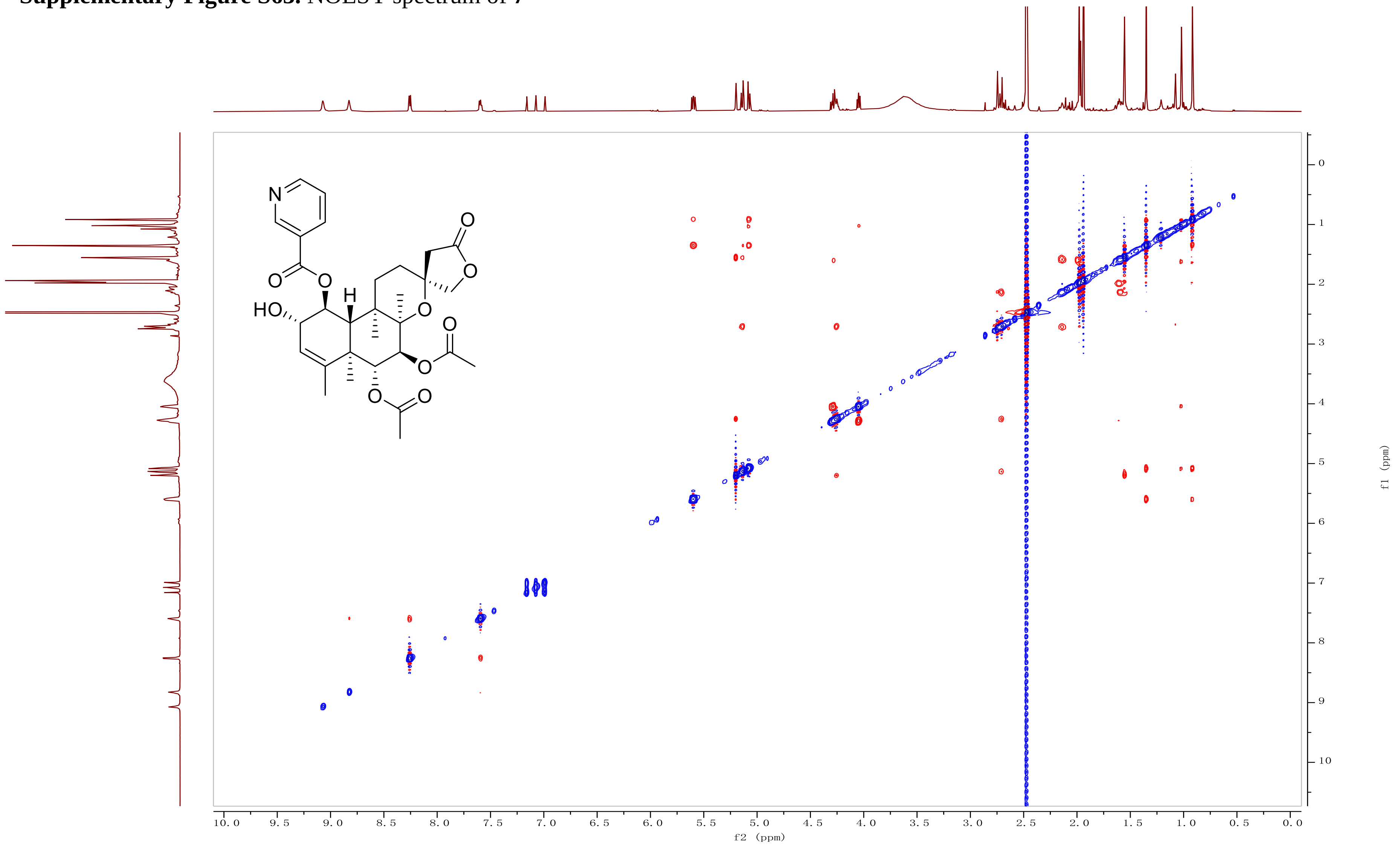

Supplementary Figure S64. HRESIMS spectrum of **7**

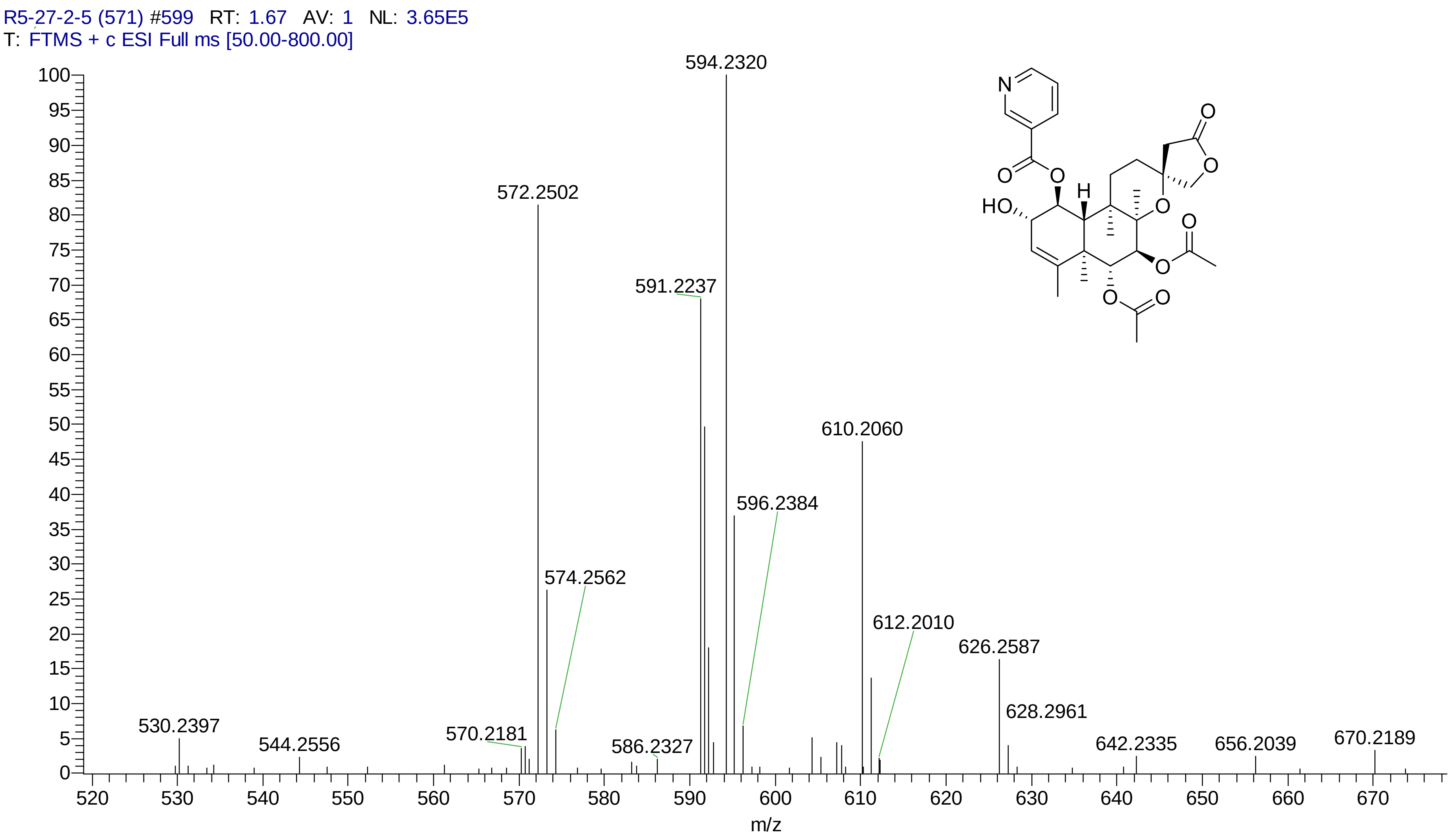

Supplementary Figure S65. IR spectrum of **7**

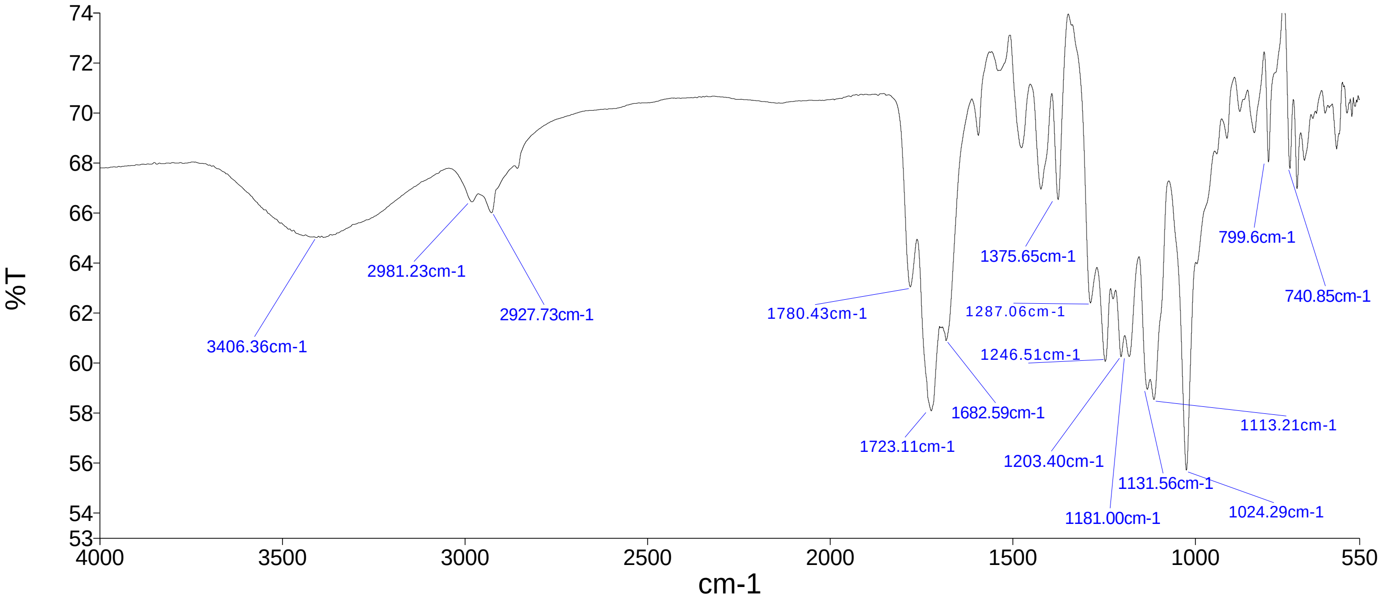

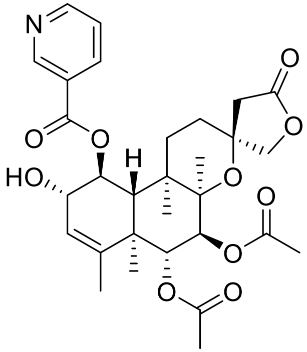

Supplementary Figure S66. UV spectrum of **7**

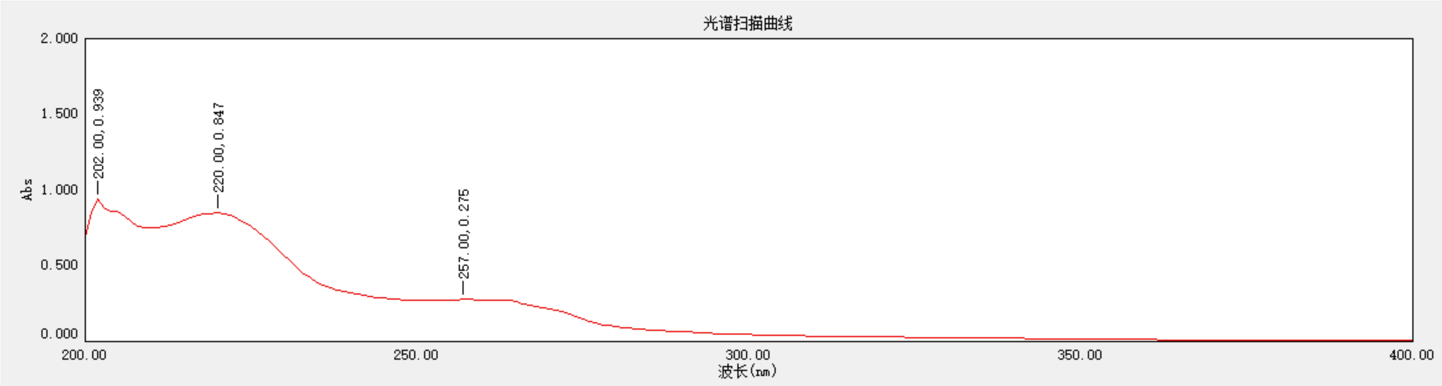

Supplementary Figure S67. CD spectrum of **7**

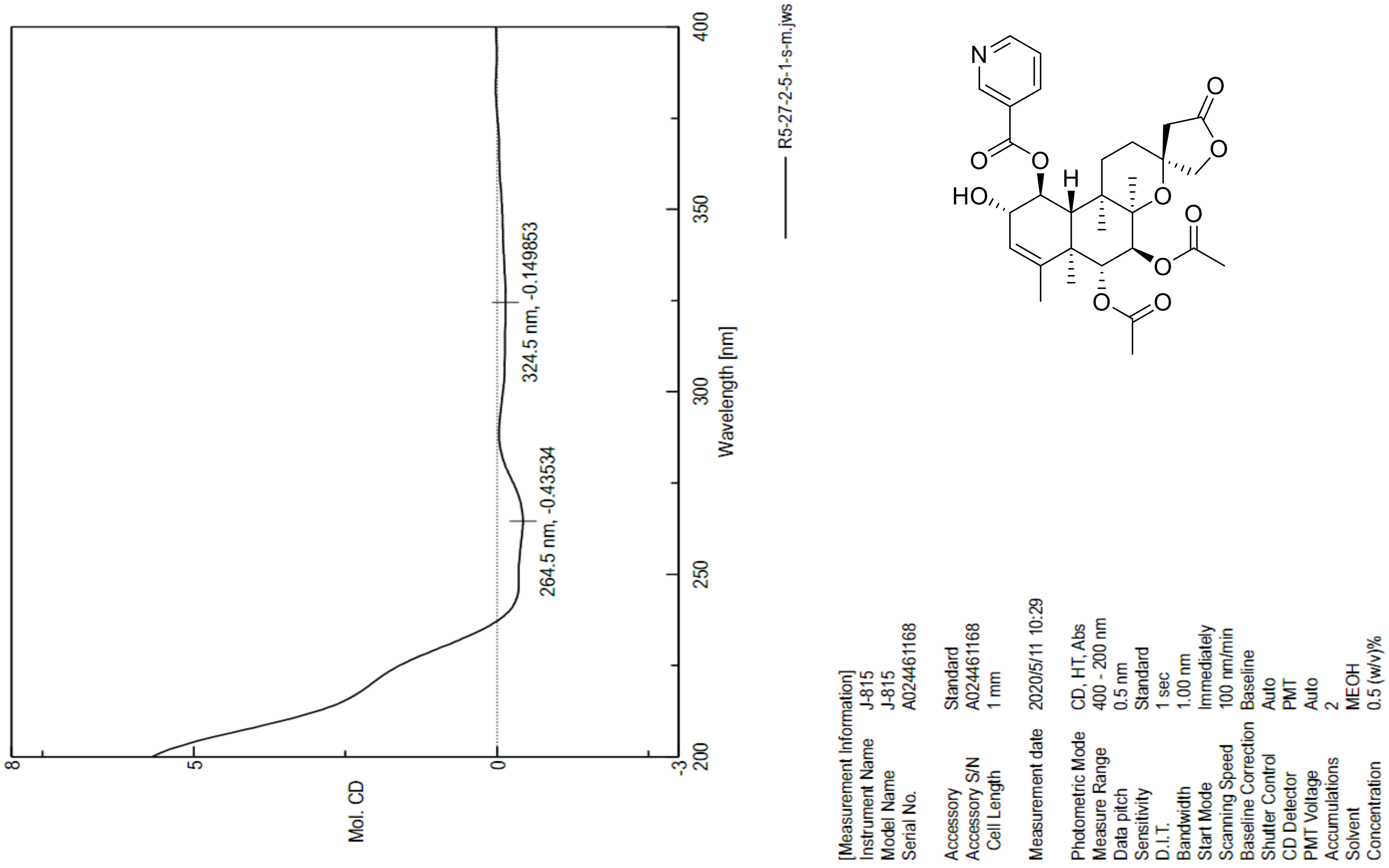

Supplementary Figure S68. ^{1}H NMR spectrum of **8**

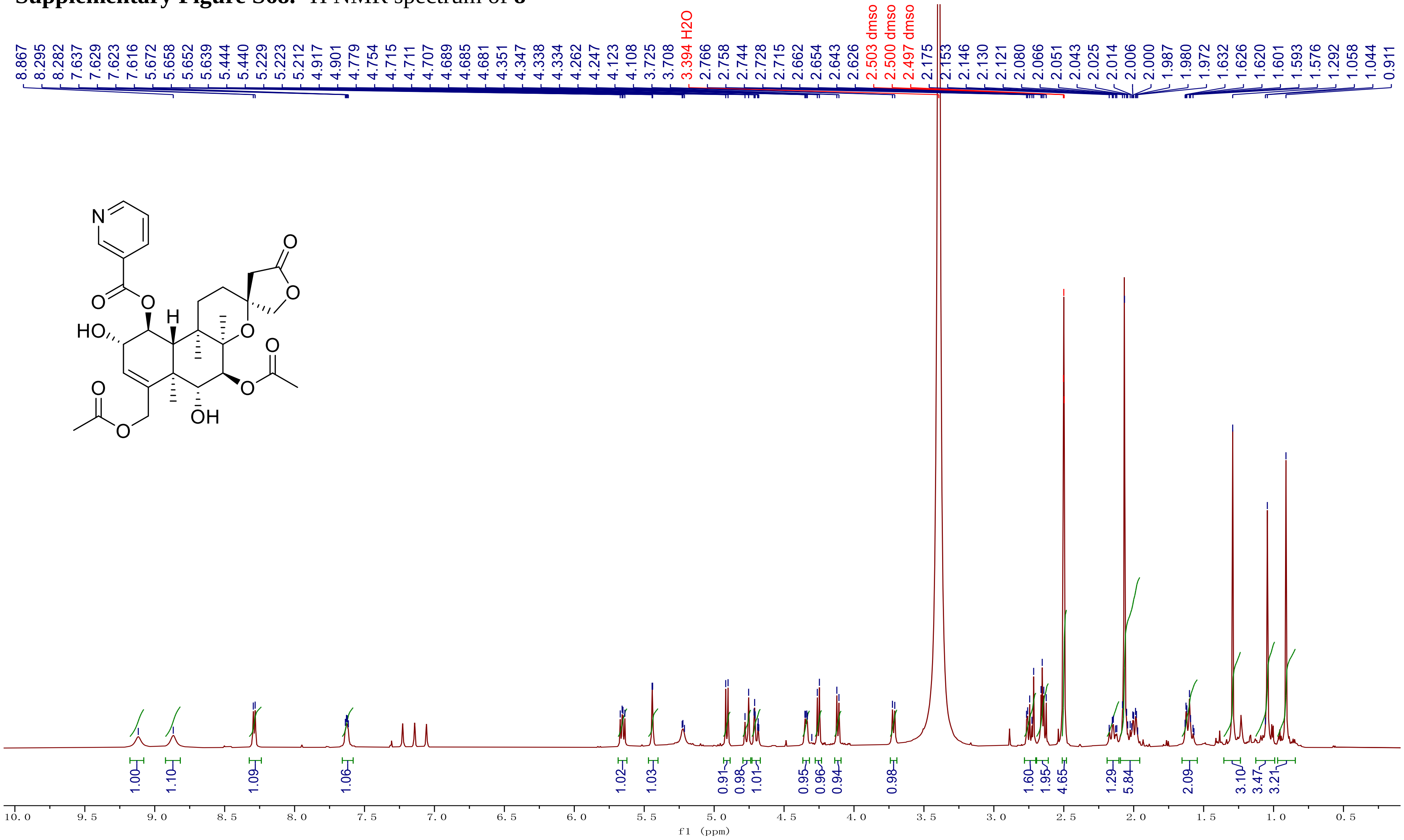

Supplementary Figure S69. ^{13}C NMR spectrum of **8**

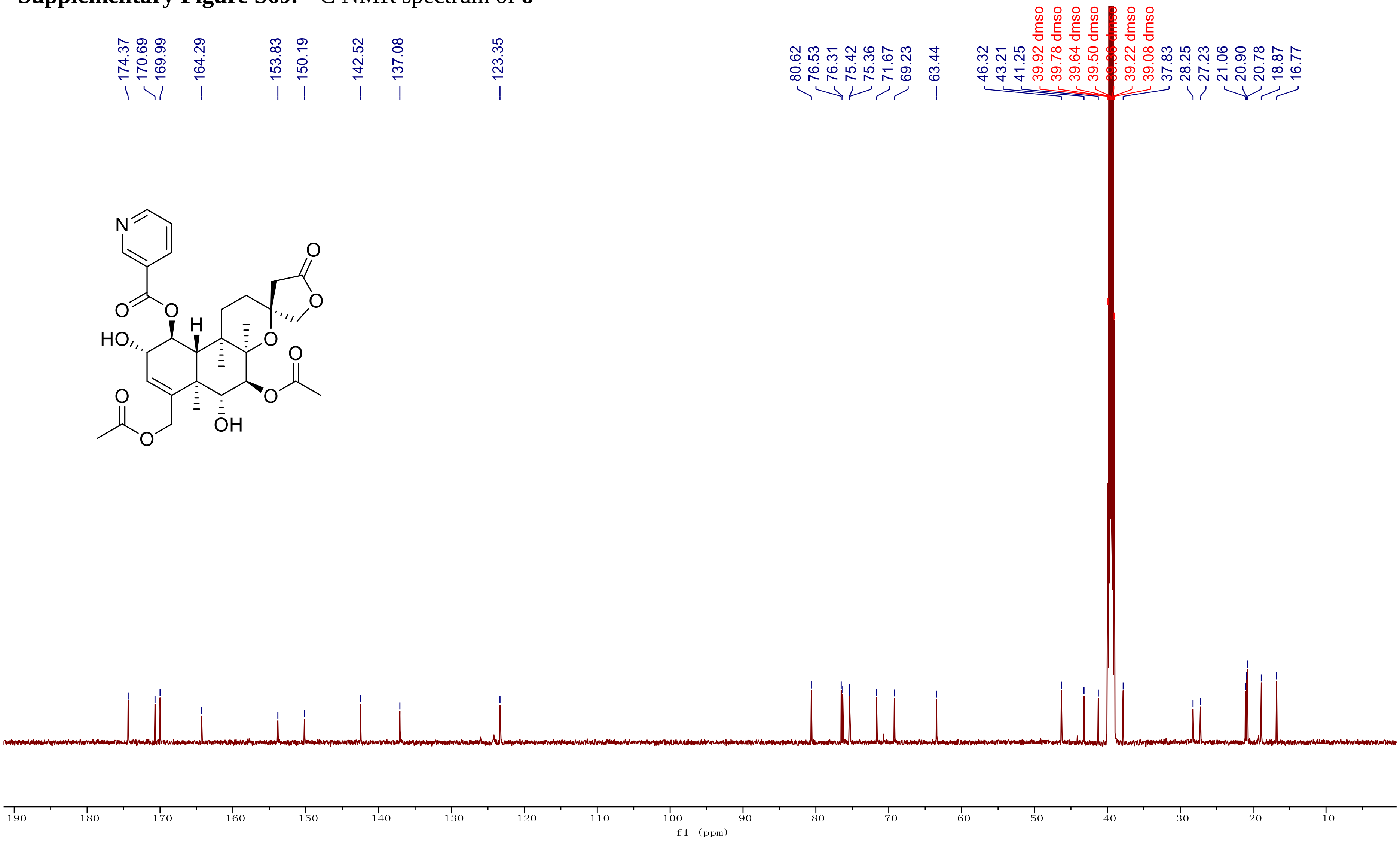

Supplementary Figure S70. DEPT spectrum of **8**

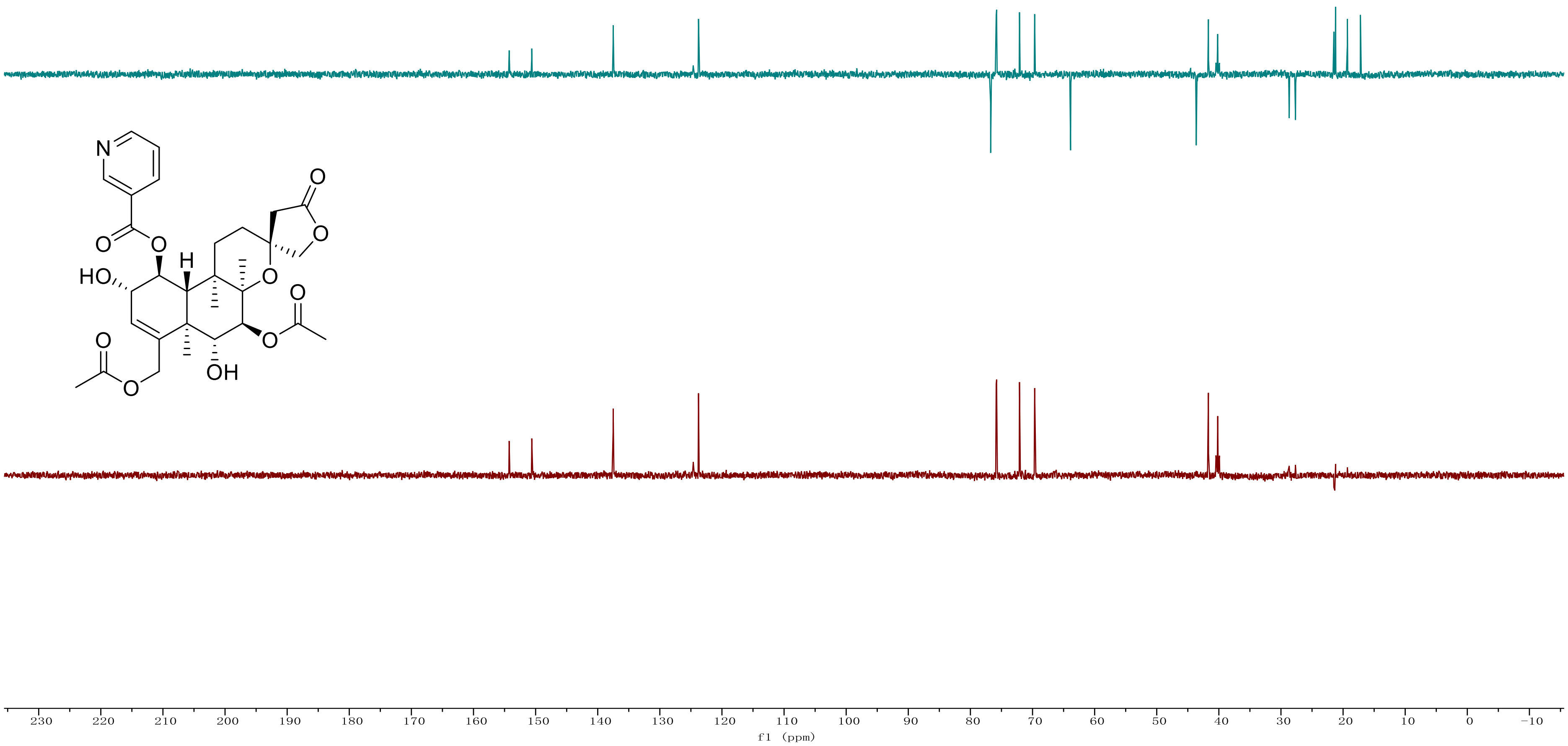

Supplementary Figure S71. ^{1}H–^{1}H COSY spectrum of **8**

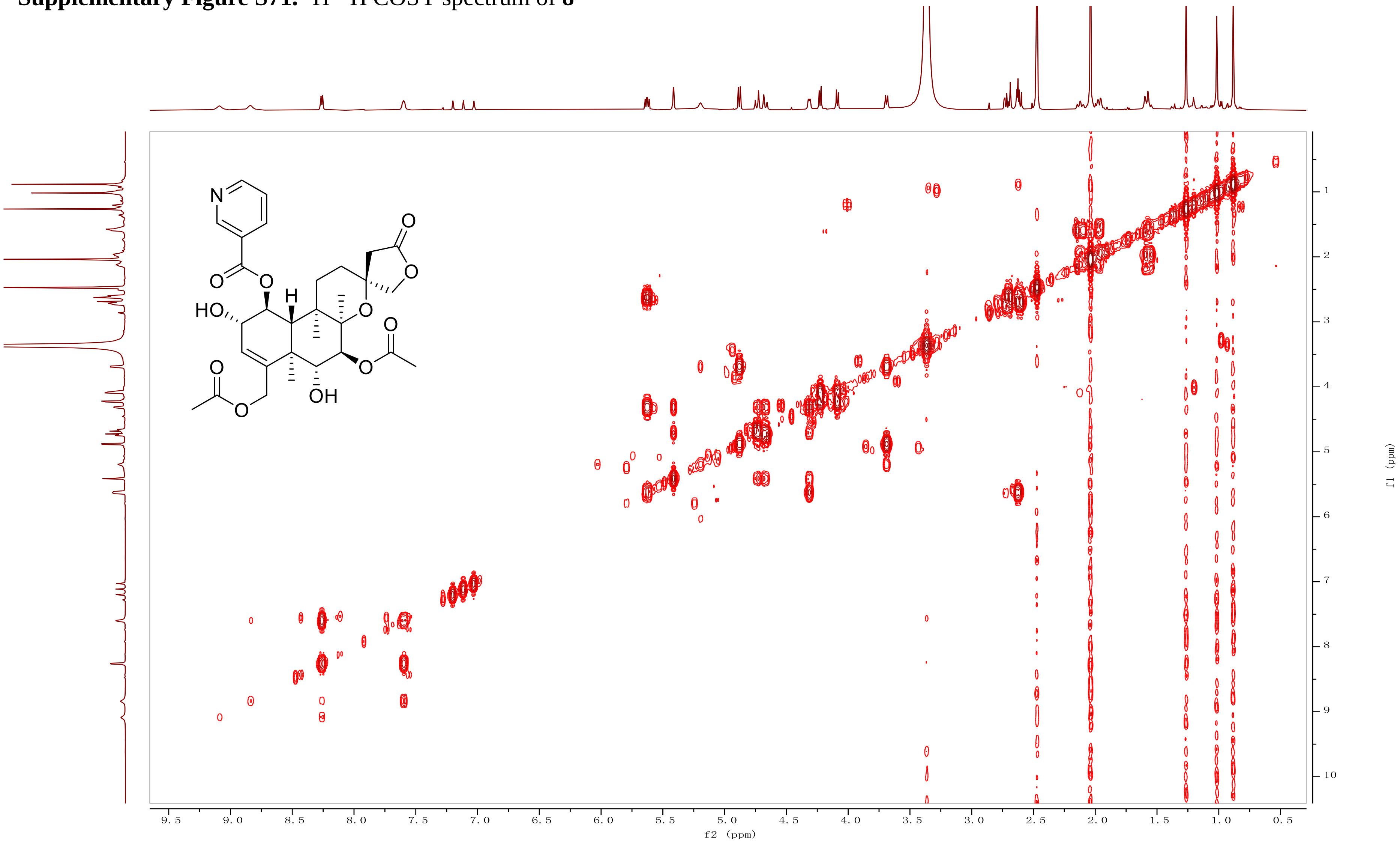

Supplementary Figure S72. HSQC spectrum of **8**

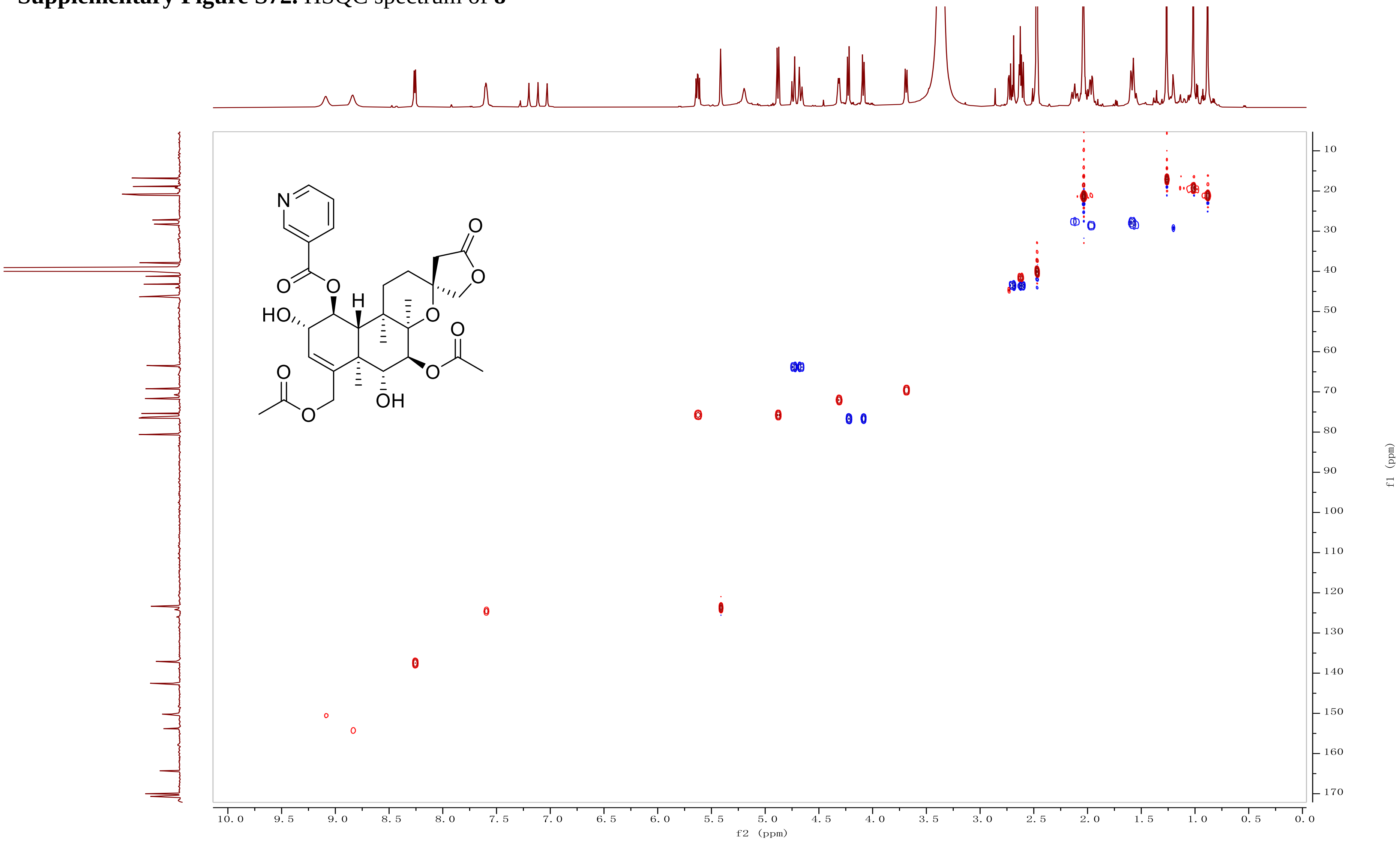

Supplementary Figure S73. HMBC spectrum of **8**

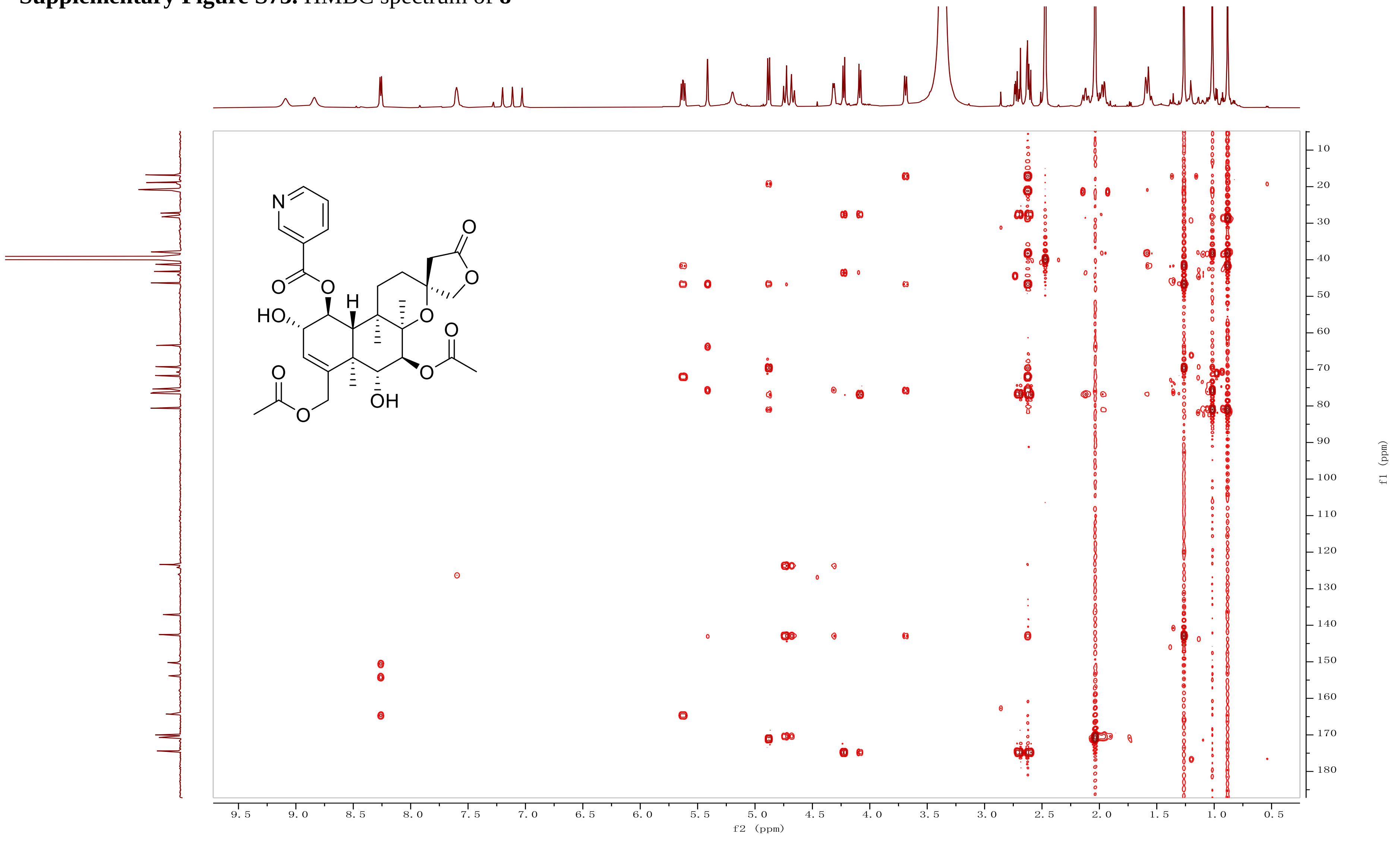

Supplementary Figure S74. 1D NOE spectrum of **8**

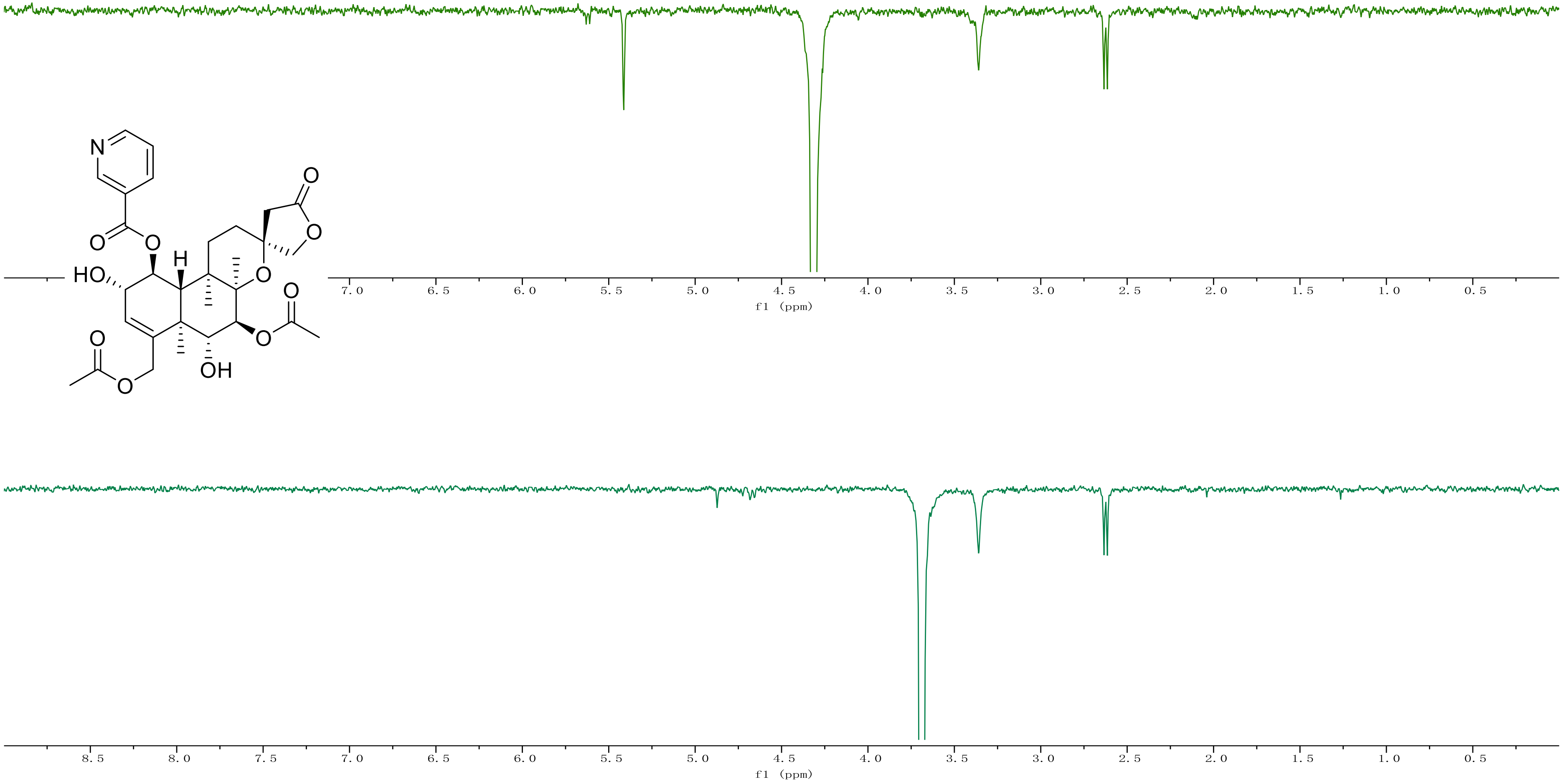

Supplementary Figure S75. HRESIMS spectrum of **8**

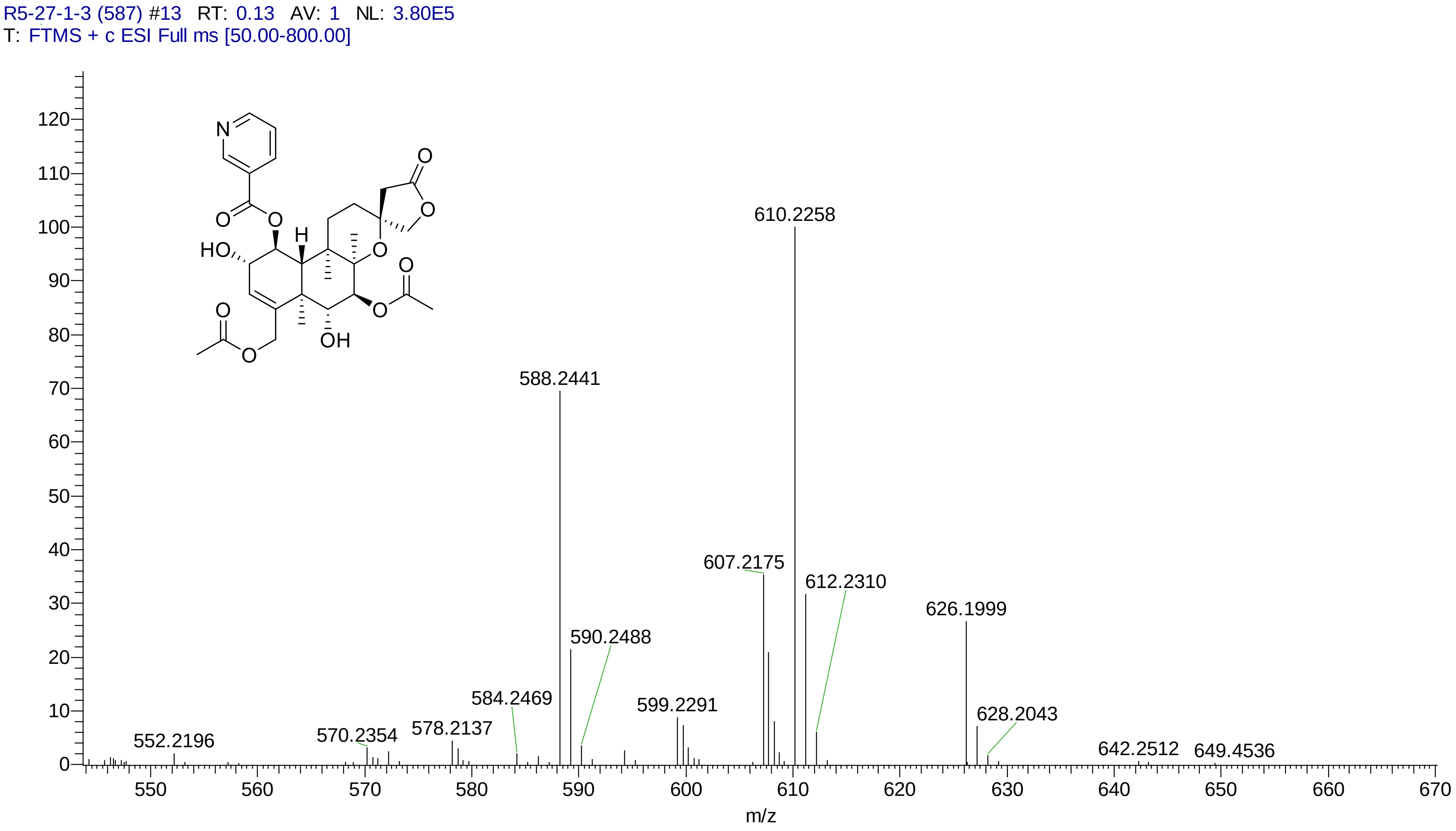

Supplementary Figure S76. IR spectrum of **8**

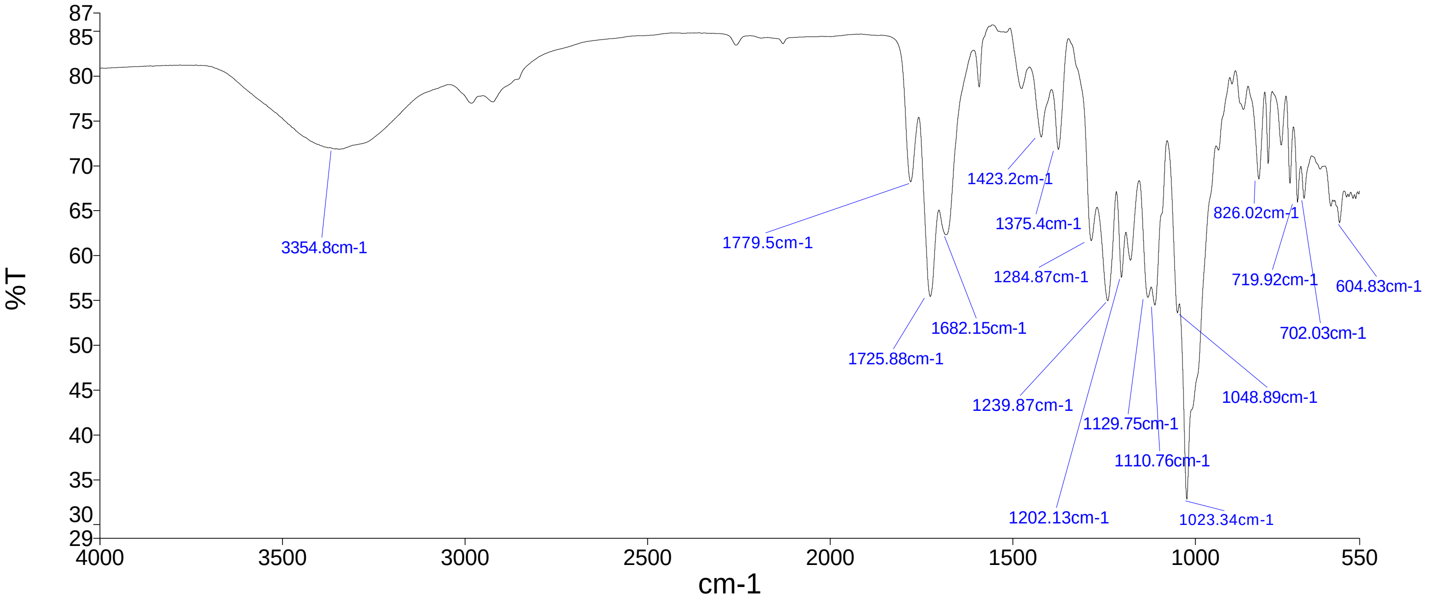

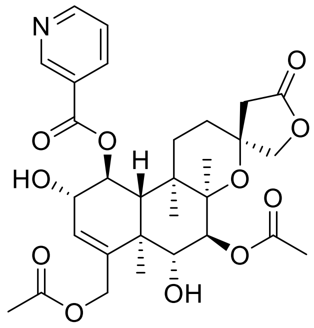

Supplementary Figure S77. UV spectrum of **8**

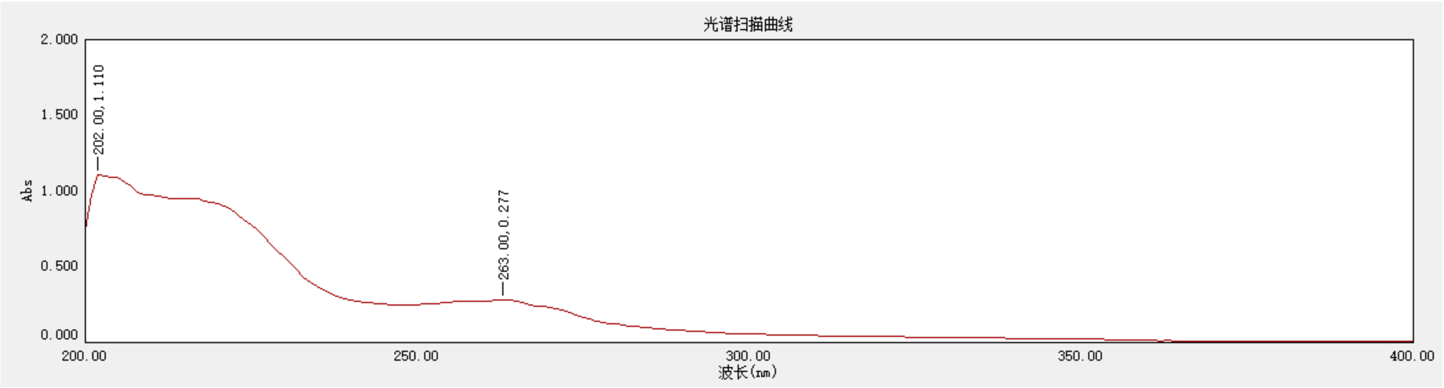

Supplementary Figure S78. CD spectrum of **8**

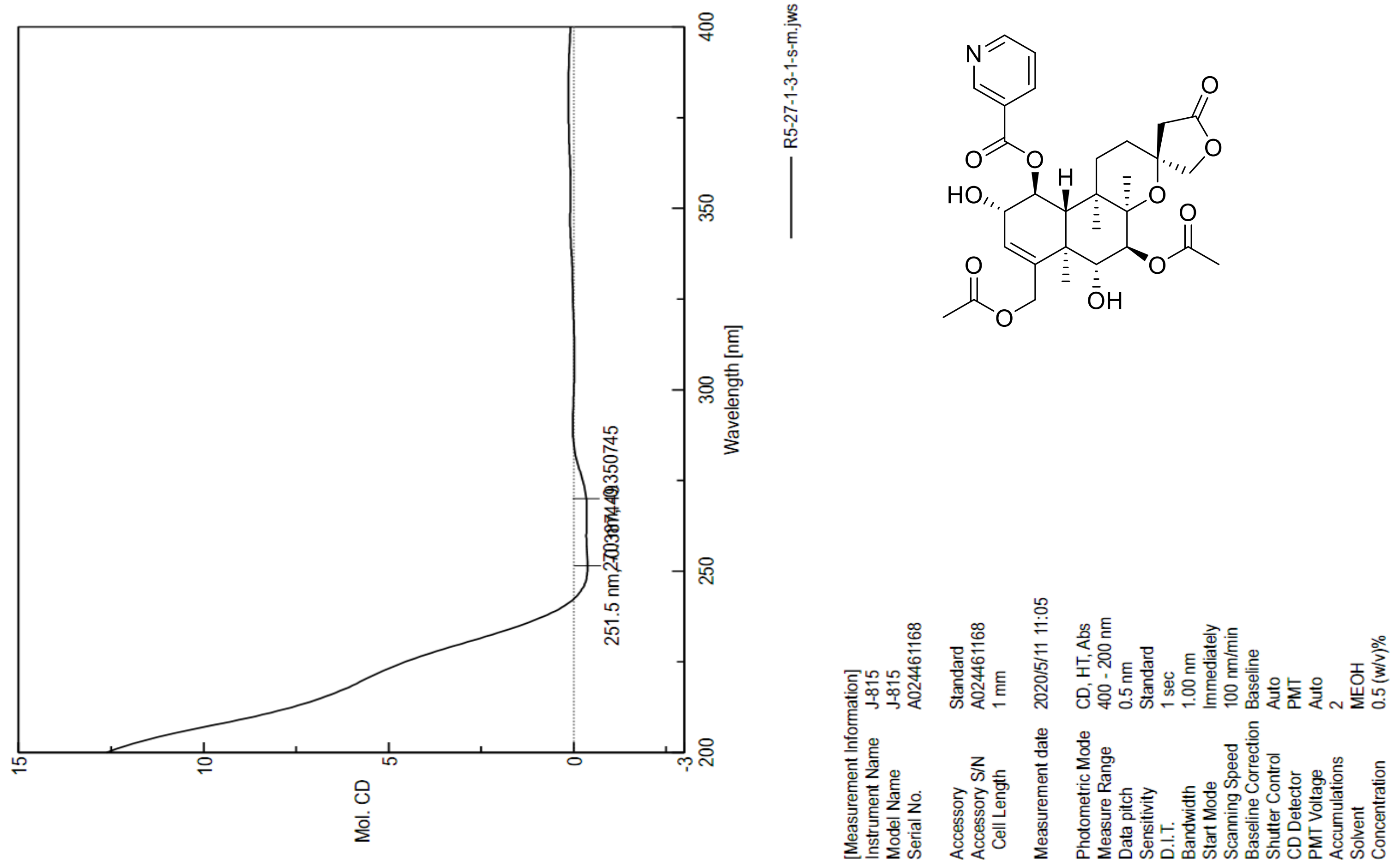

Supplementary Figure S79. ^{1}H NMR spectrum of **9**

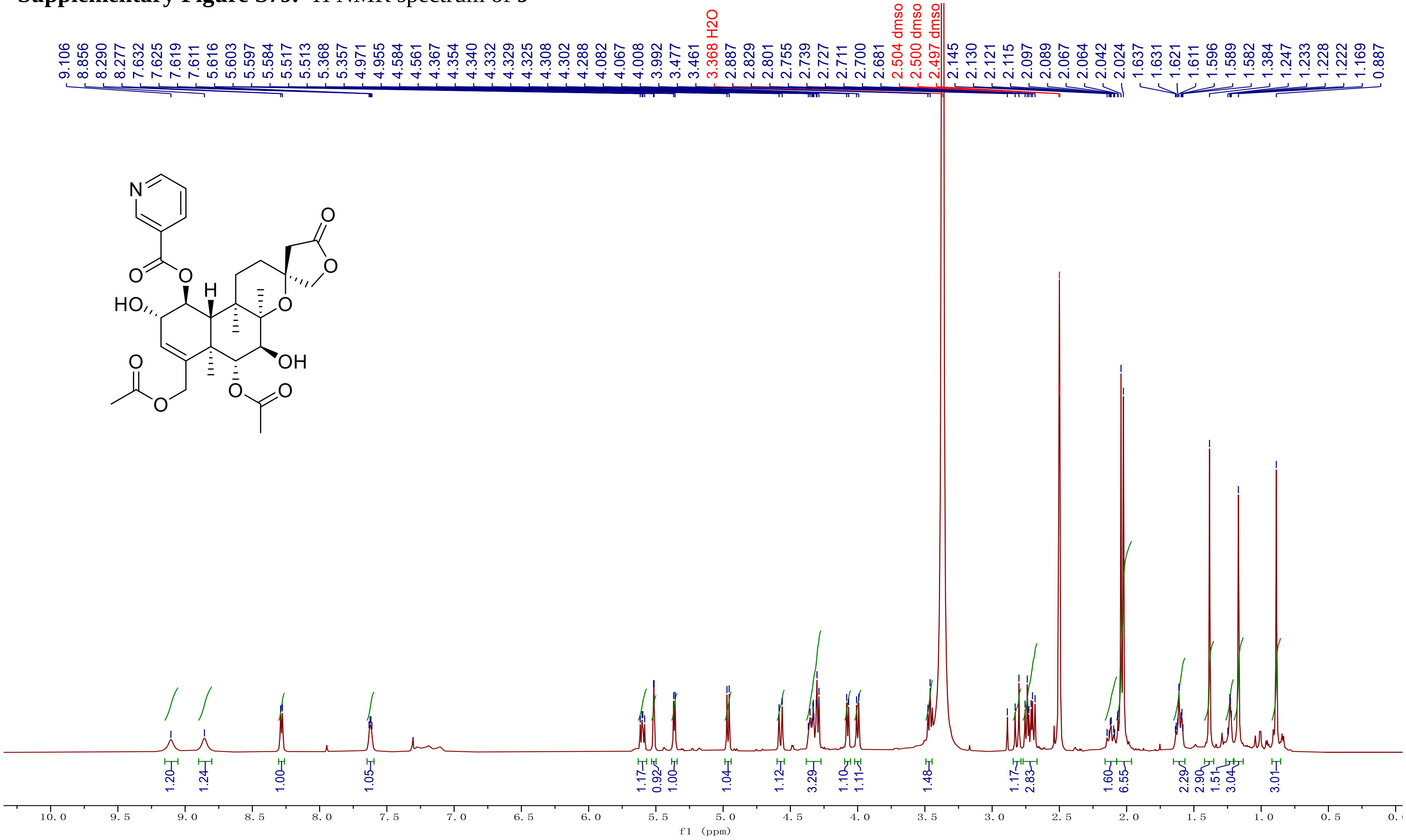

Supplementary Figure S80. ^{13}C NMR spectrum of **9**

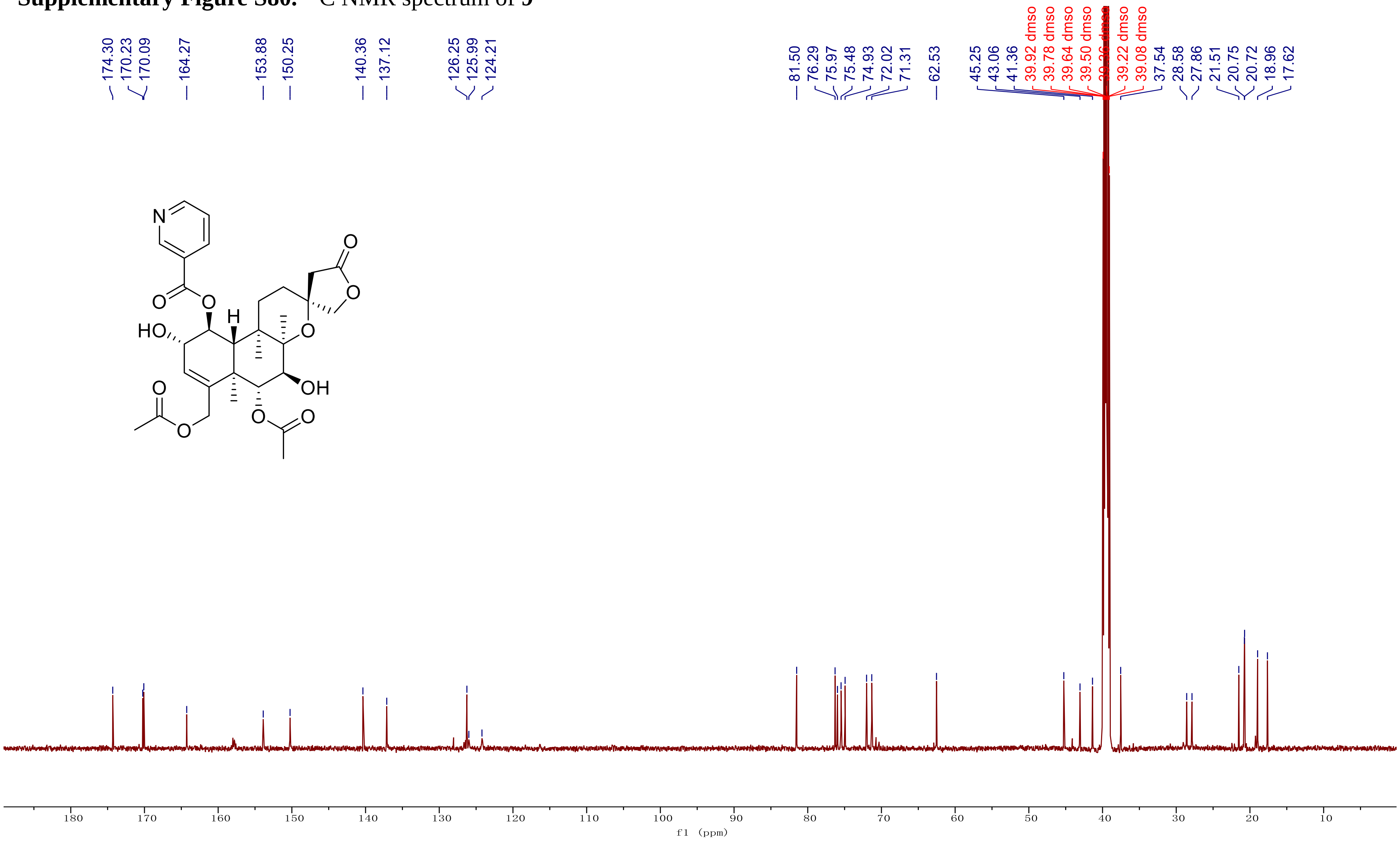

Supplementary Figure S81. DEPT spectrum of **9**

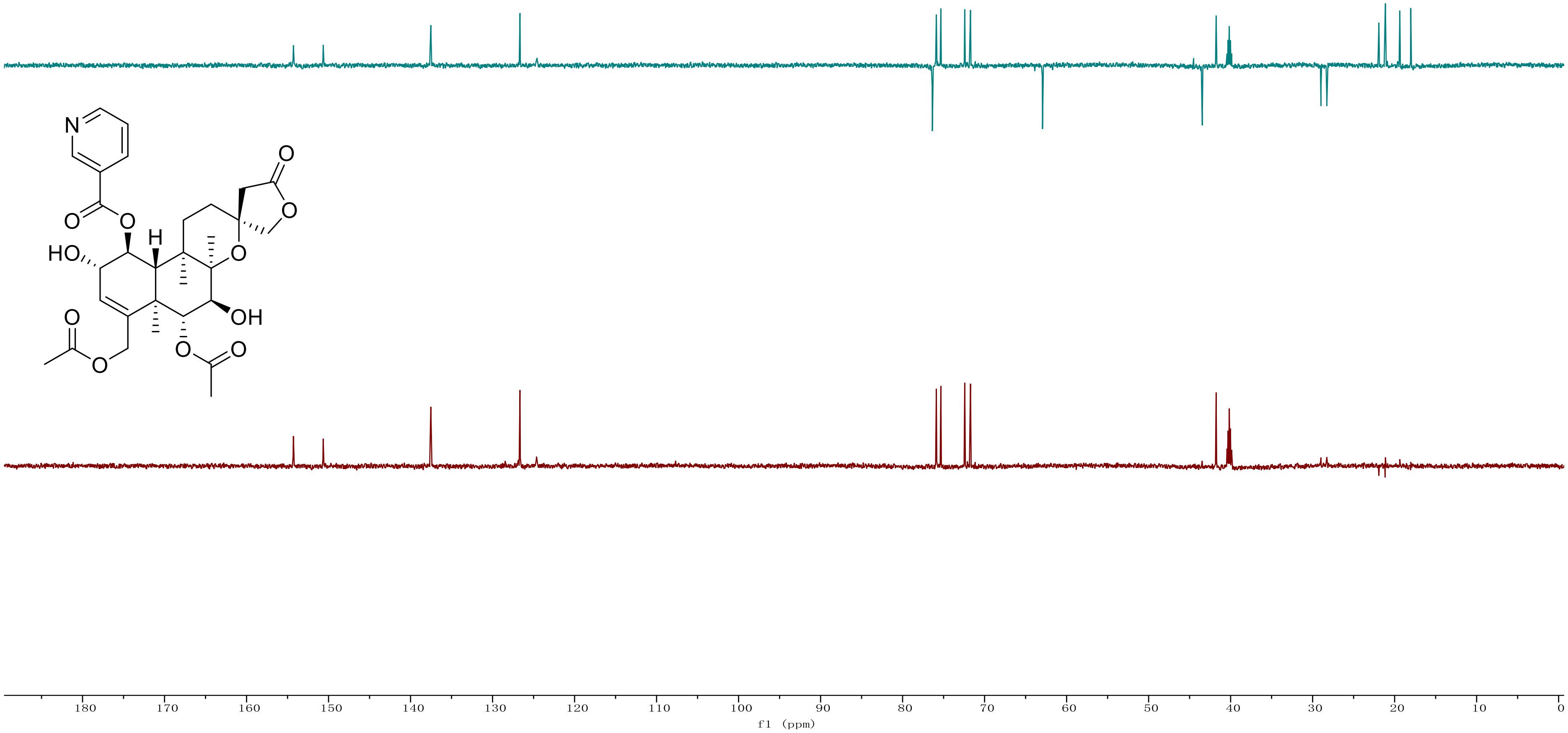

Supplementary Figure S82. ^{1}H–^{1}H COSY spectrum of **9**

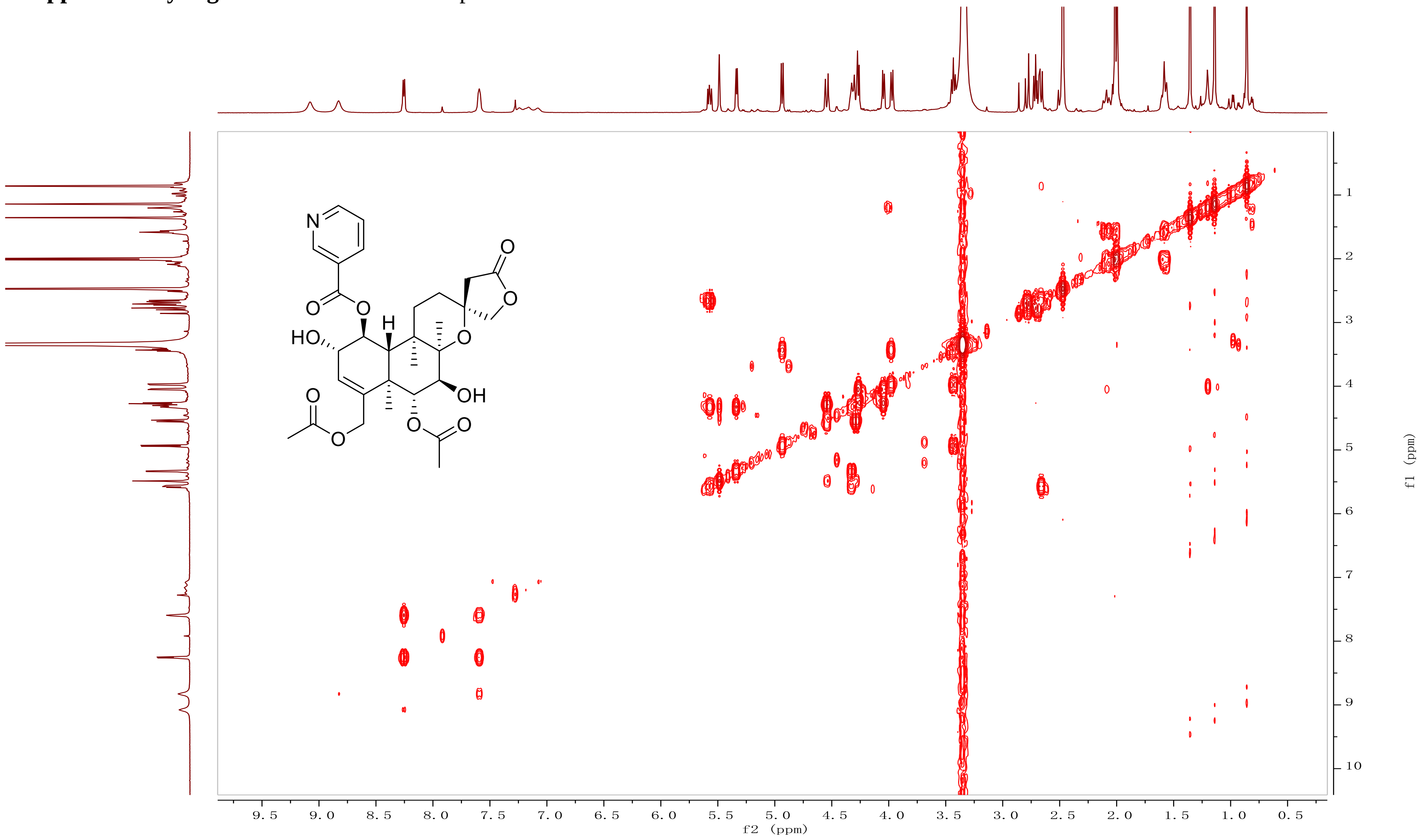

Supplementary Figure S83. HSQC spectrum of **9**

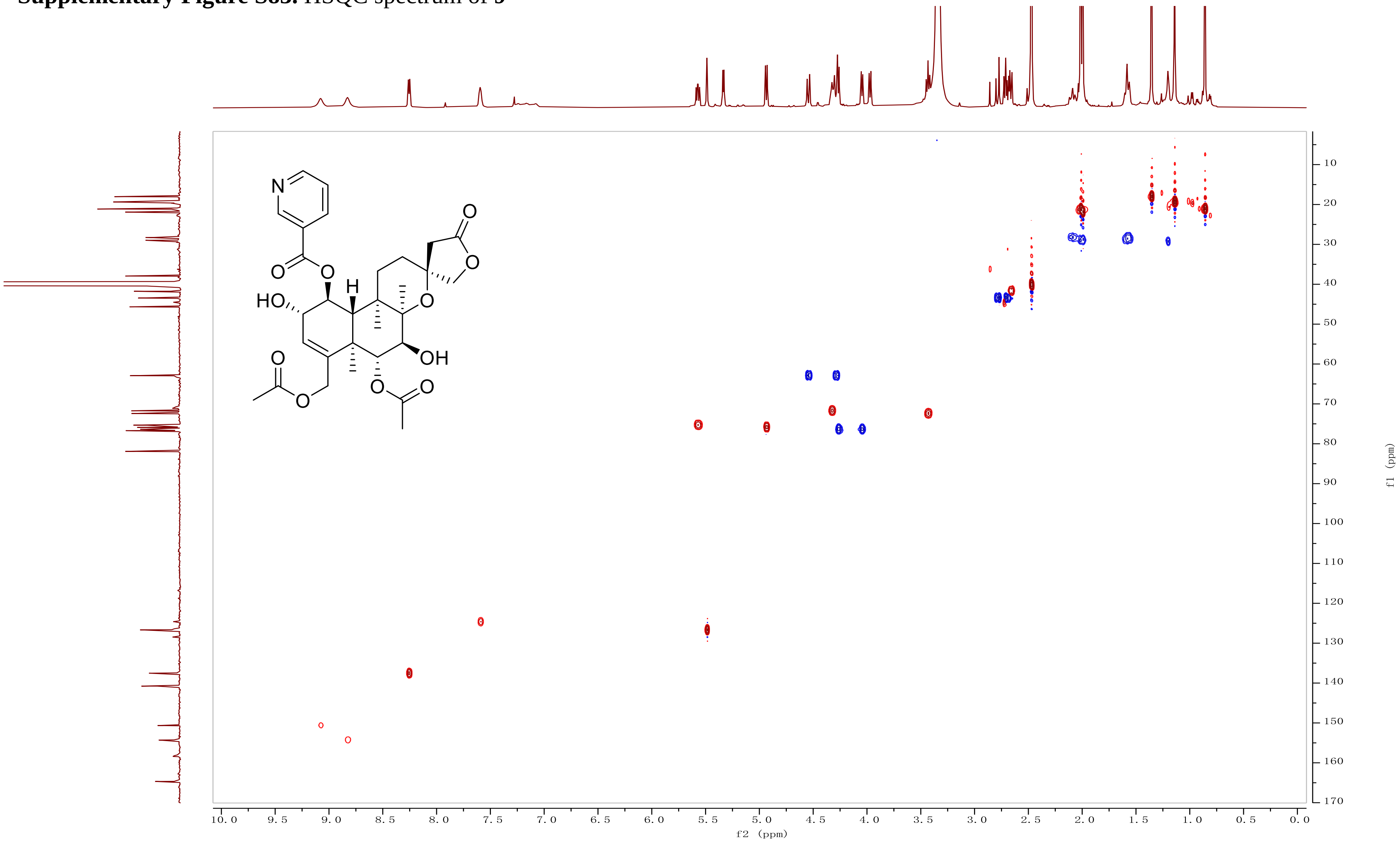

Supplementary Figure S84. HMBC spectrum of **9**

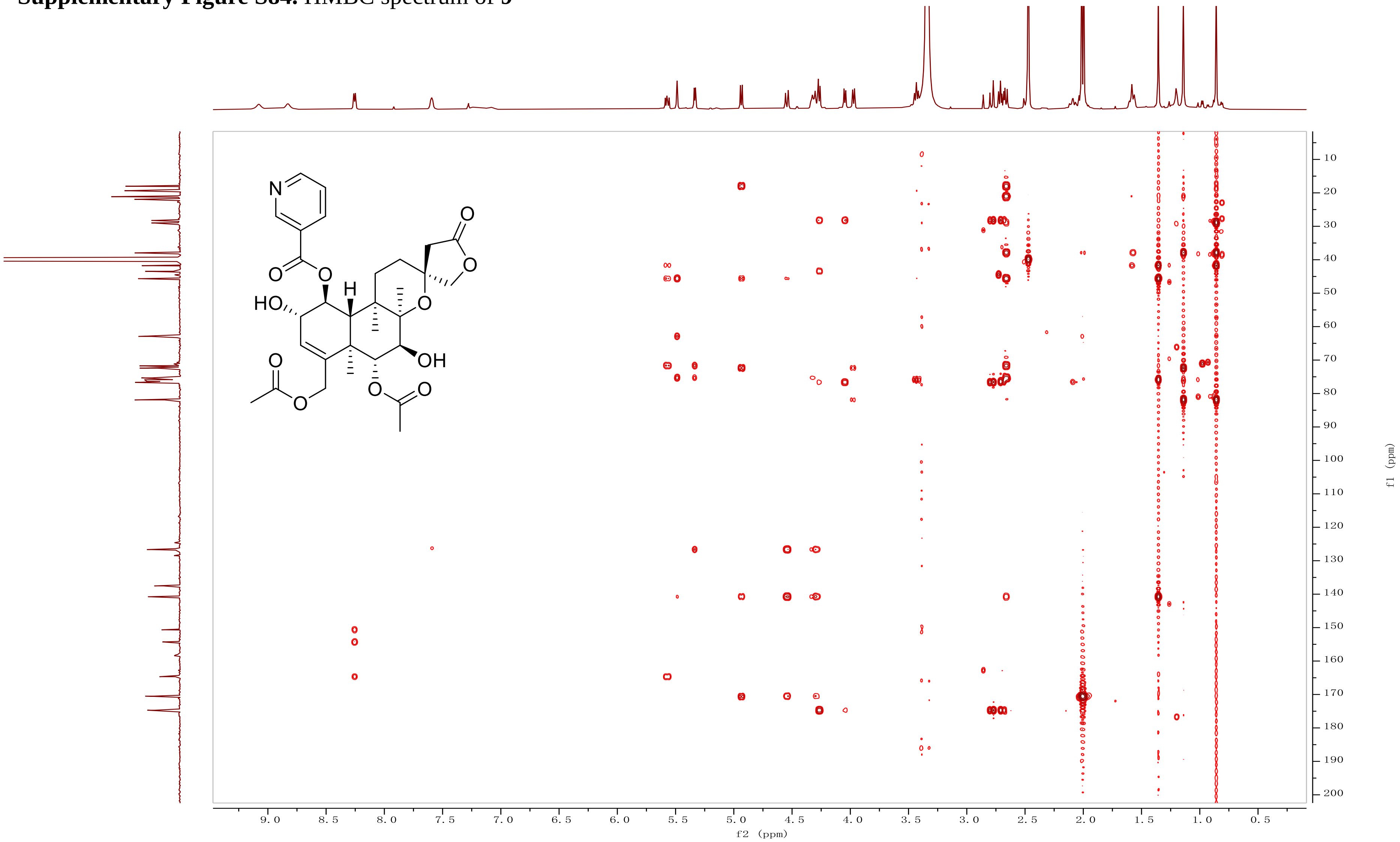

Supplementary Figure S85. NOESY spectrum of **9**

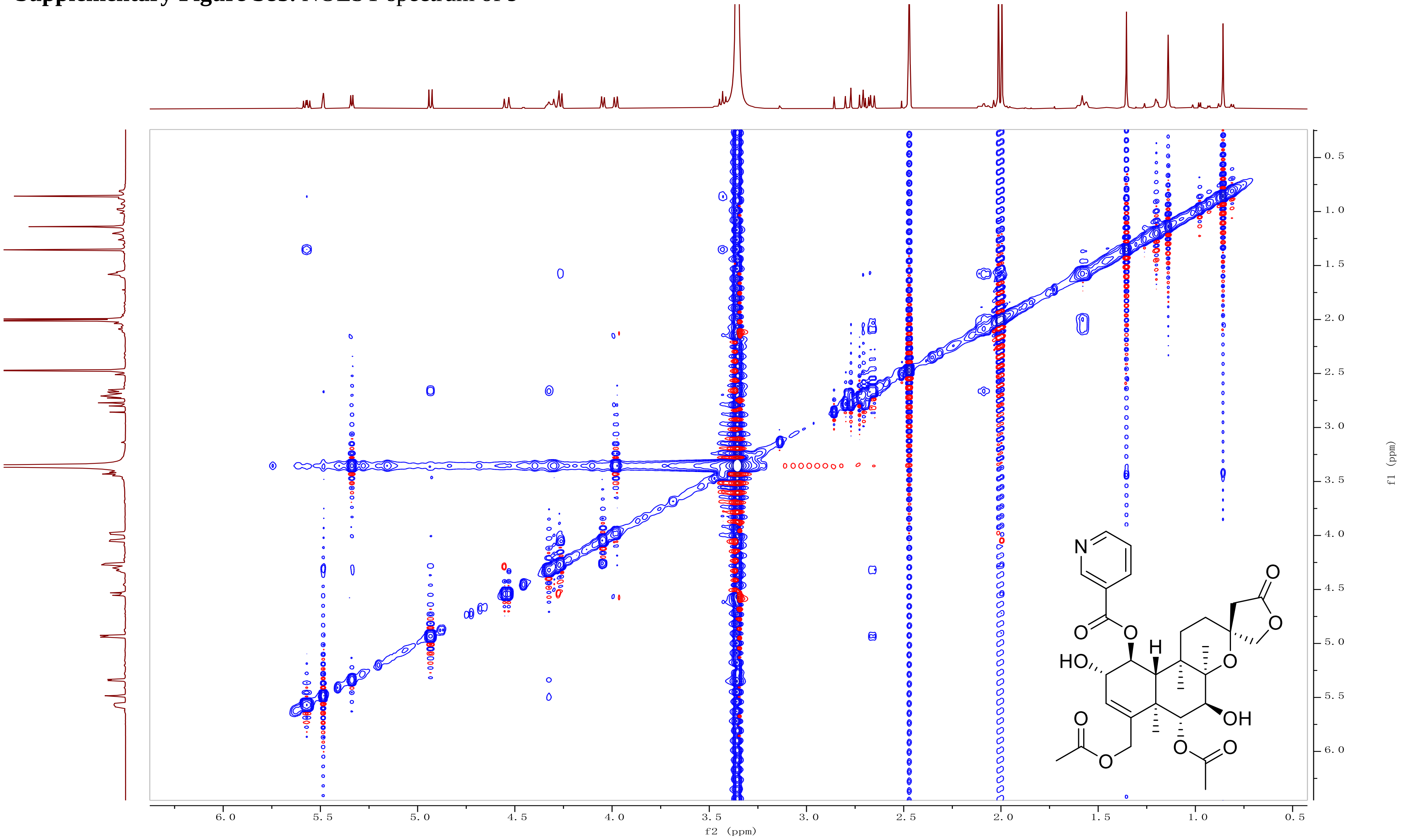

Supplementary Figure S86. HRESIMS spectrum of **9**

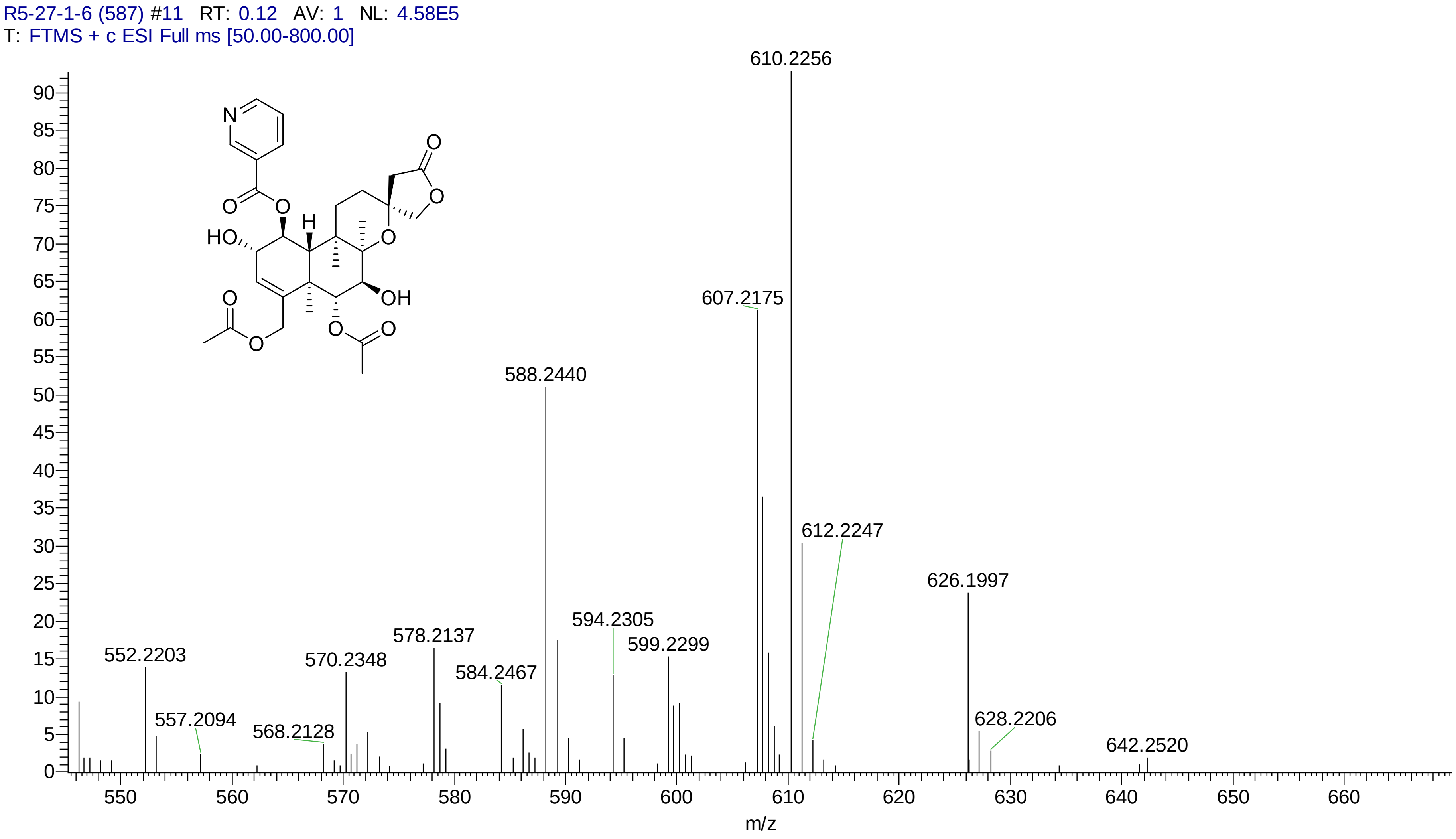

Supplementary Figure S87. IR spectrum of **9**

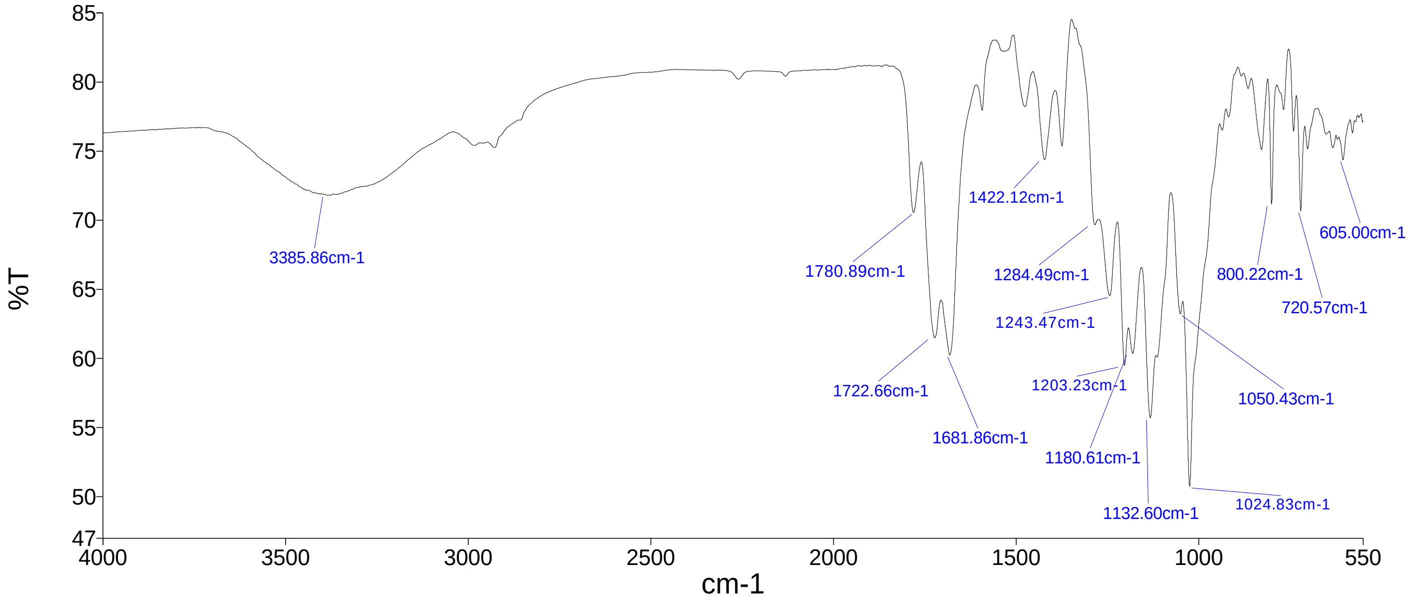

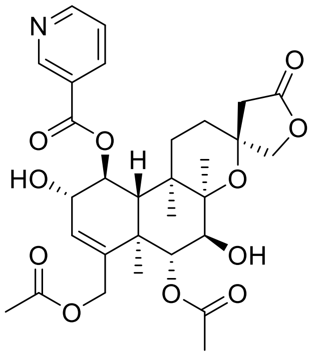

Supplementary Figure S88. UV spectrum of **9**

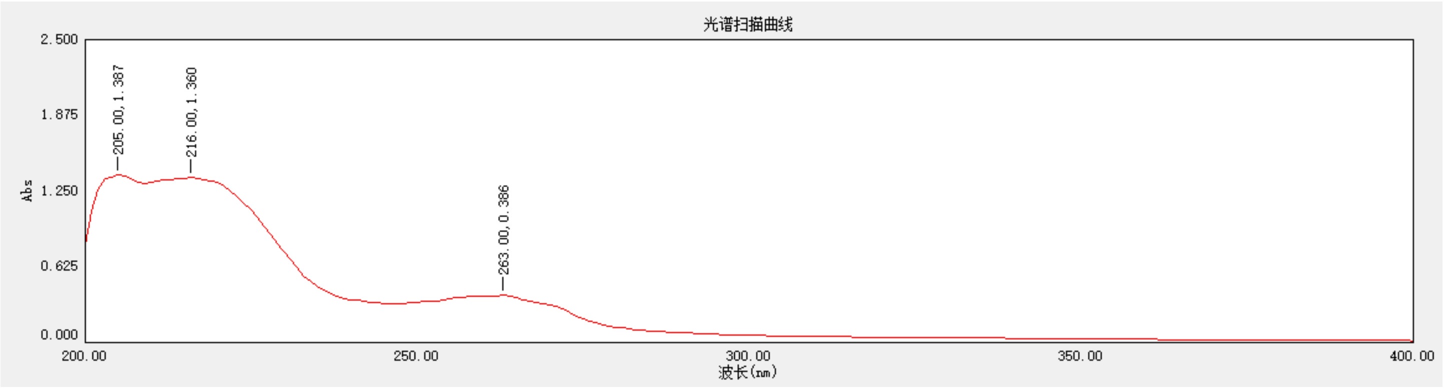

Supplementary Figure S89. CD spectrum of **9**

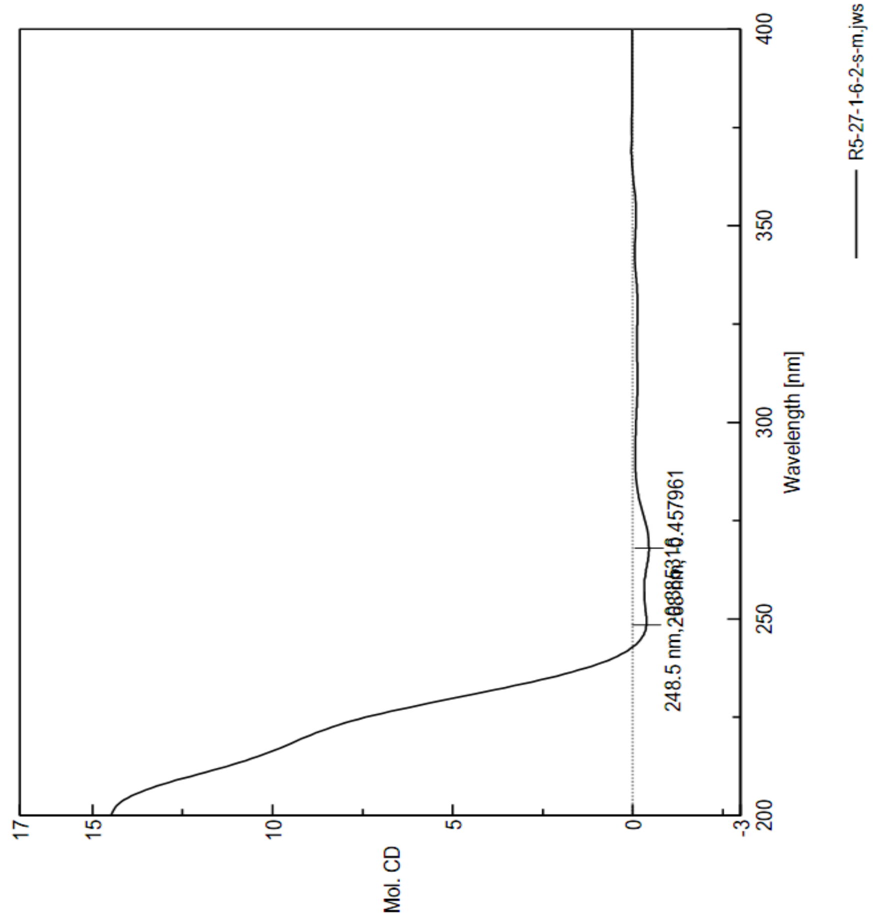

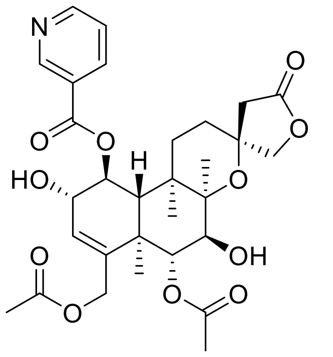

[Measurement Information]	
Instrument Name	J-815
Model Name	J-815
Serial No.	A024461168
Accessory	Standard
Accessory S/N	A024461168
Cell Length	1 mm
Measurement date	2020/5/11 10:58
Photometric Mode	CD, HT, Abs
Measure Range	400 - 200 nm
Data pitch	0.5 nm
Sensitivity	Standard
D.I.T.	1 sec
Bandwidth	1.00 nm
Start Mode	Immediately
Scanning Speed	100 nm/min
Baseline Correction	Baseline
Shutter Control	Auto
CD Detector	PMT
PMT Voltage	Auto
Accumulations	2
Solvent	MEOH
Concentration	0.33 (w/v)%

Supplementary Figure S90. ^{1}H NMR spectrum of **10**

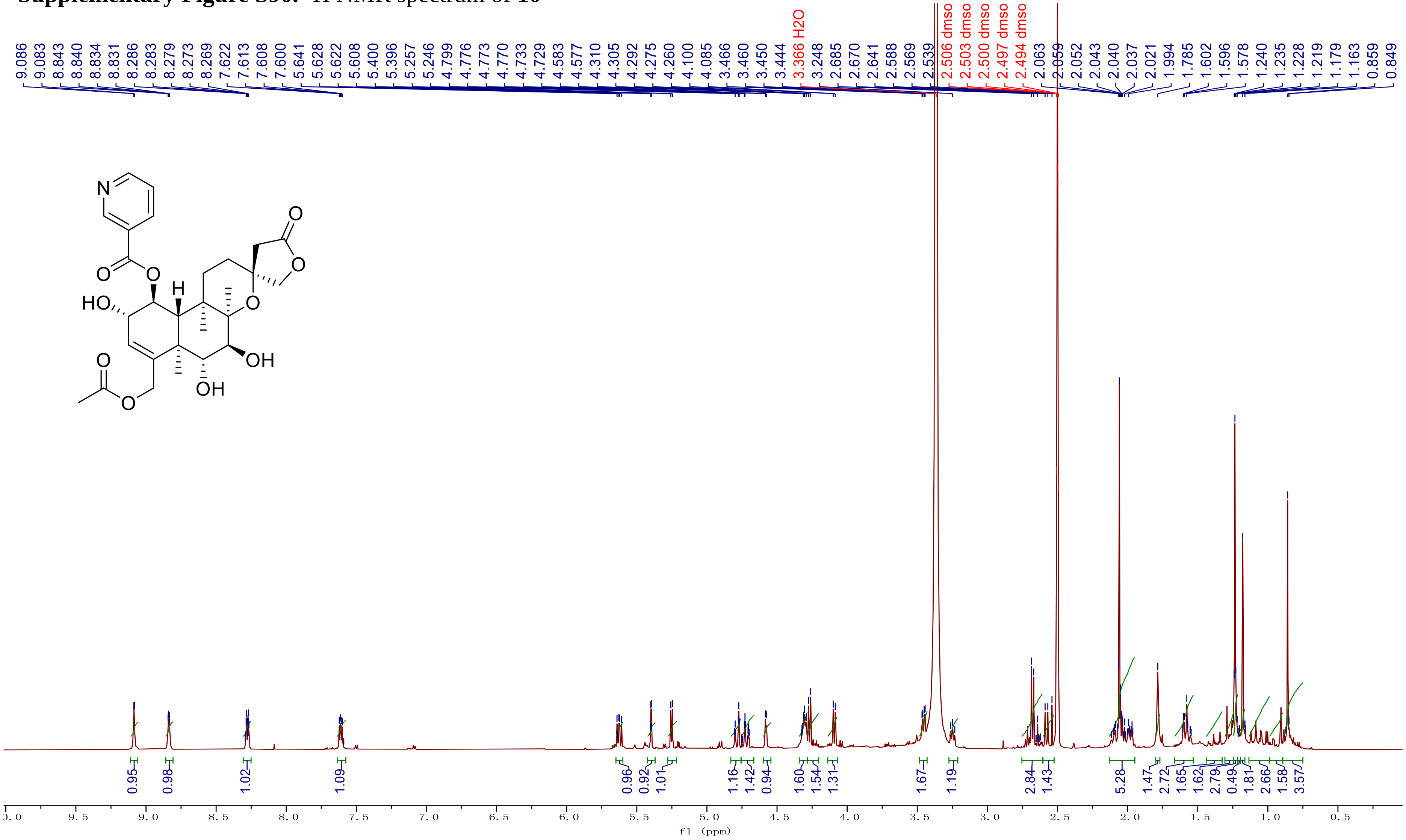

Supplementary Figure S91. ^{13}C NMR spectrum of **10**

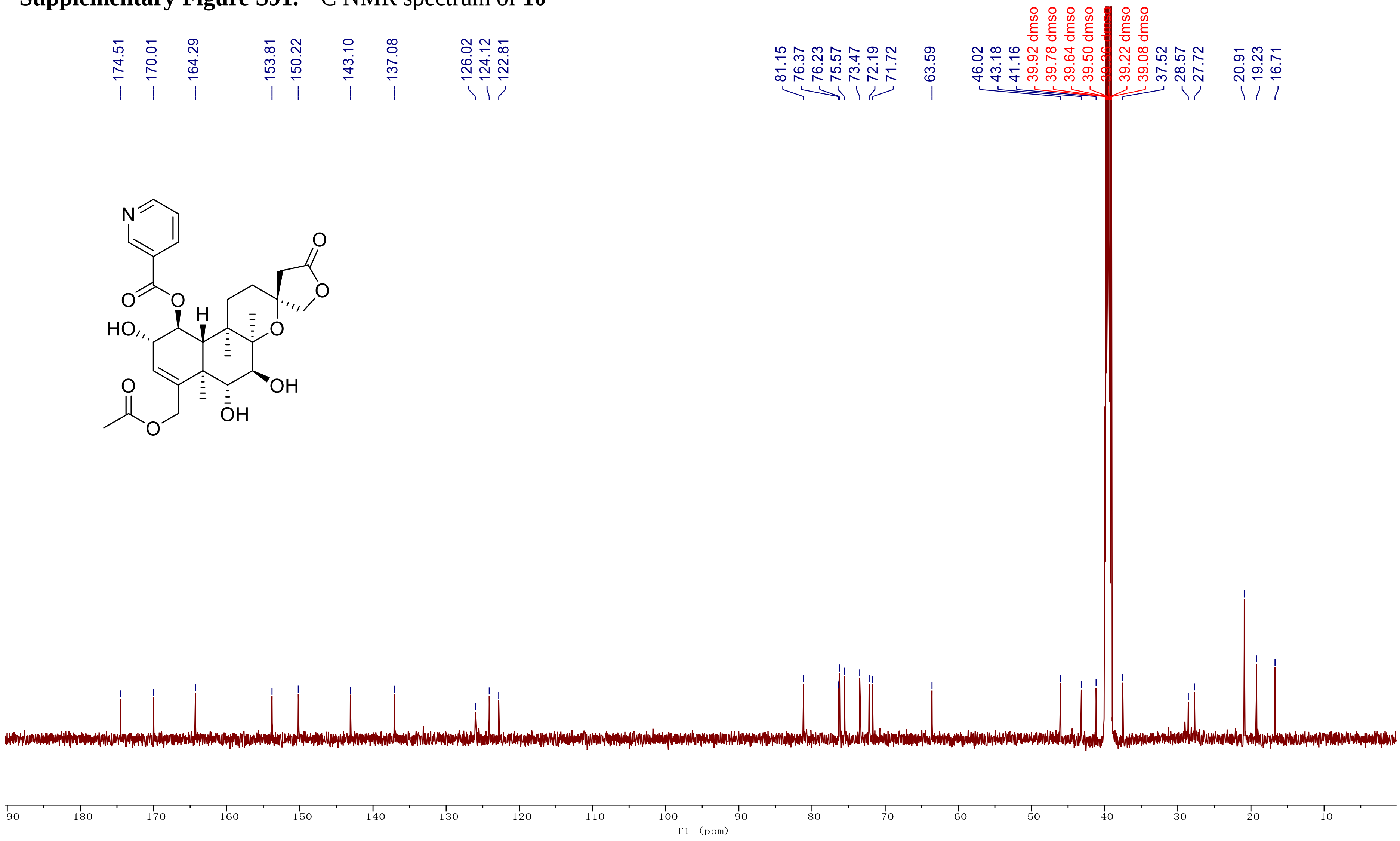

Supplementary Figure S92. DEPT spectrum of **10**

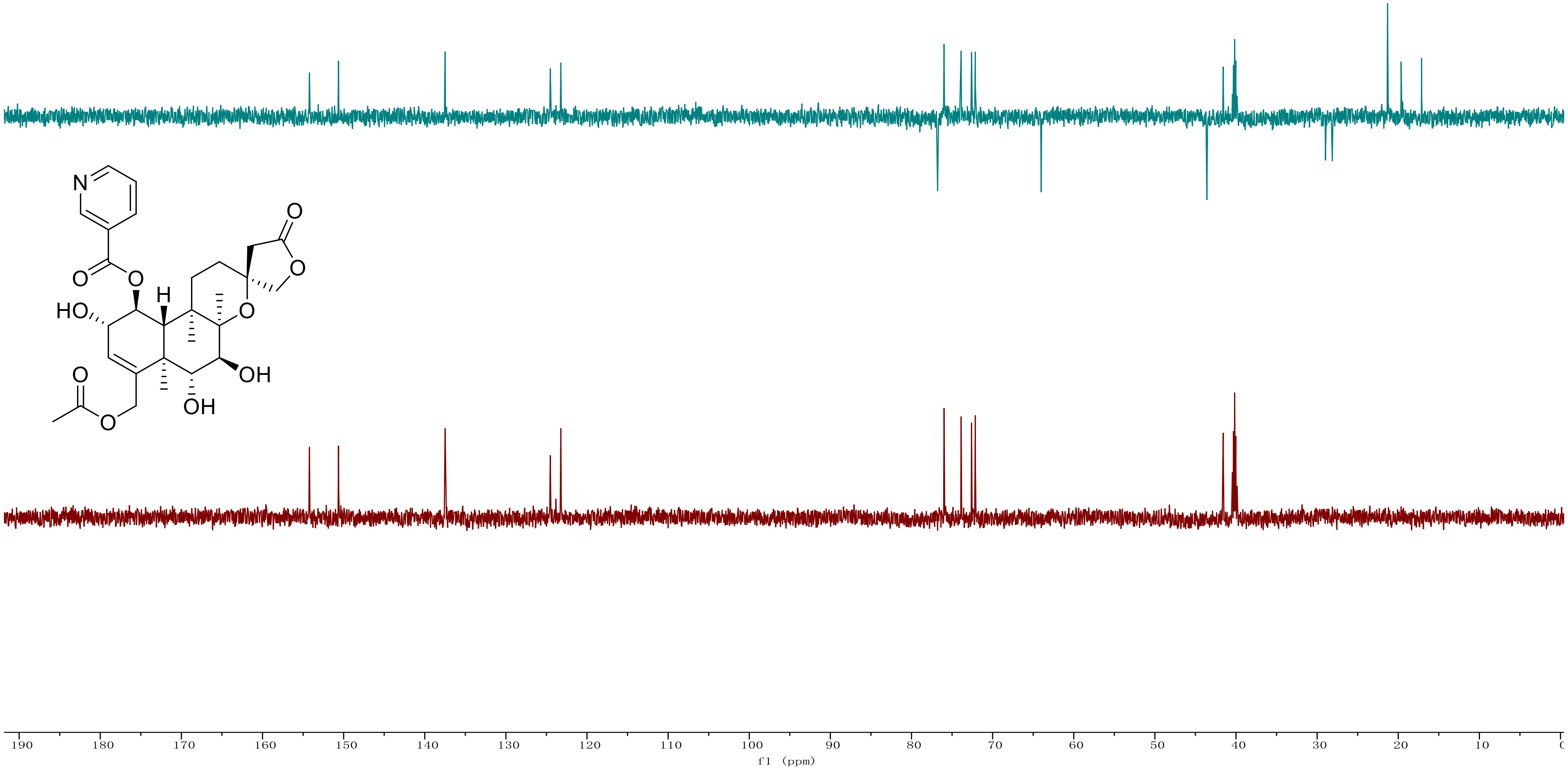

Supplementary Figure S93. ^1H–^1H COSY spectrum of **10**

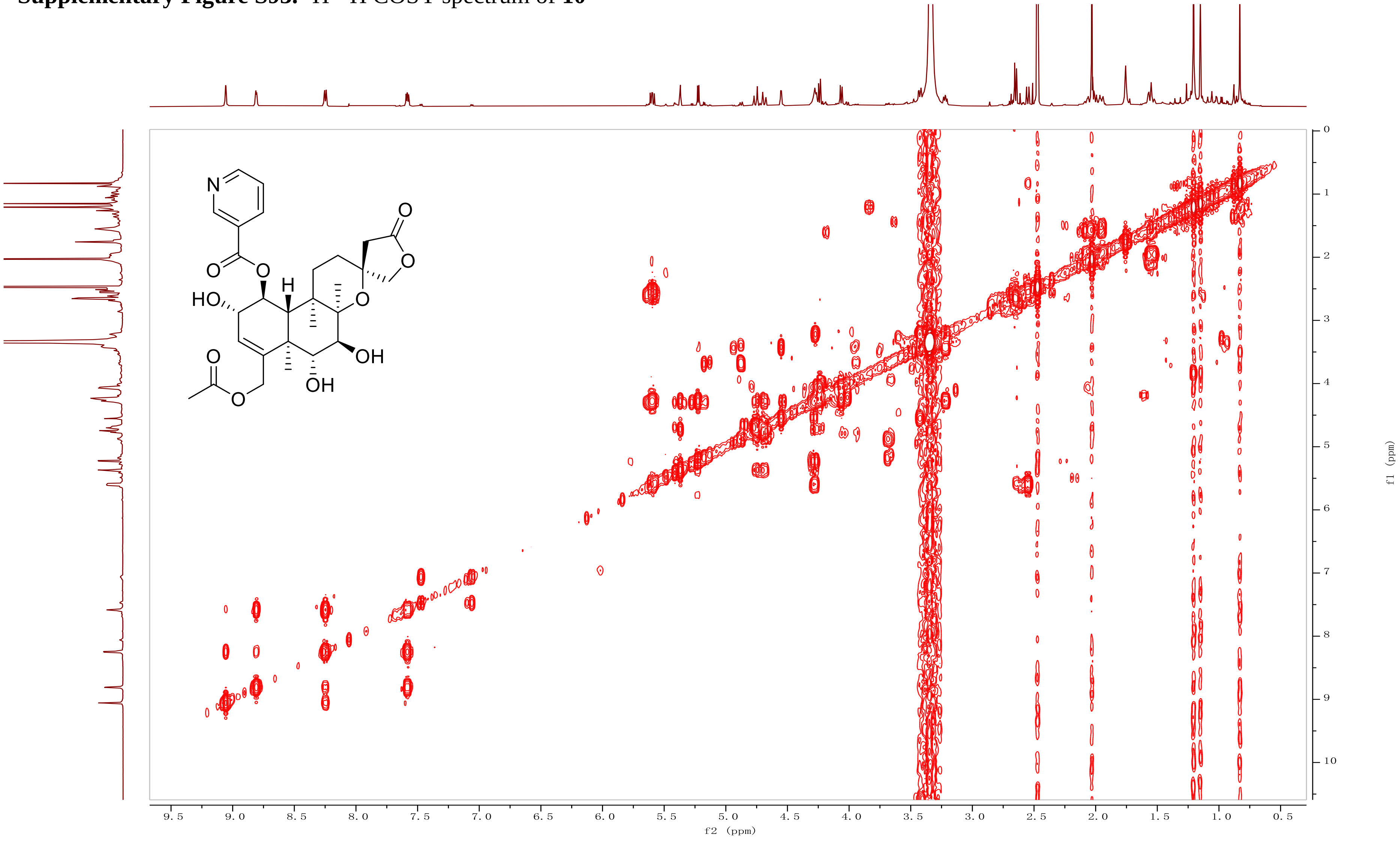

Supplementary Figure S94. HSQC spectrum of **10**

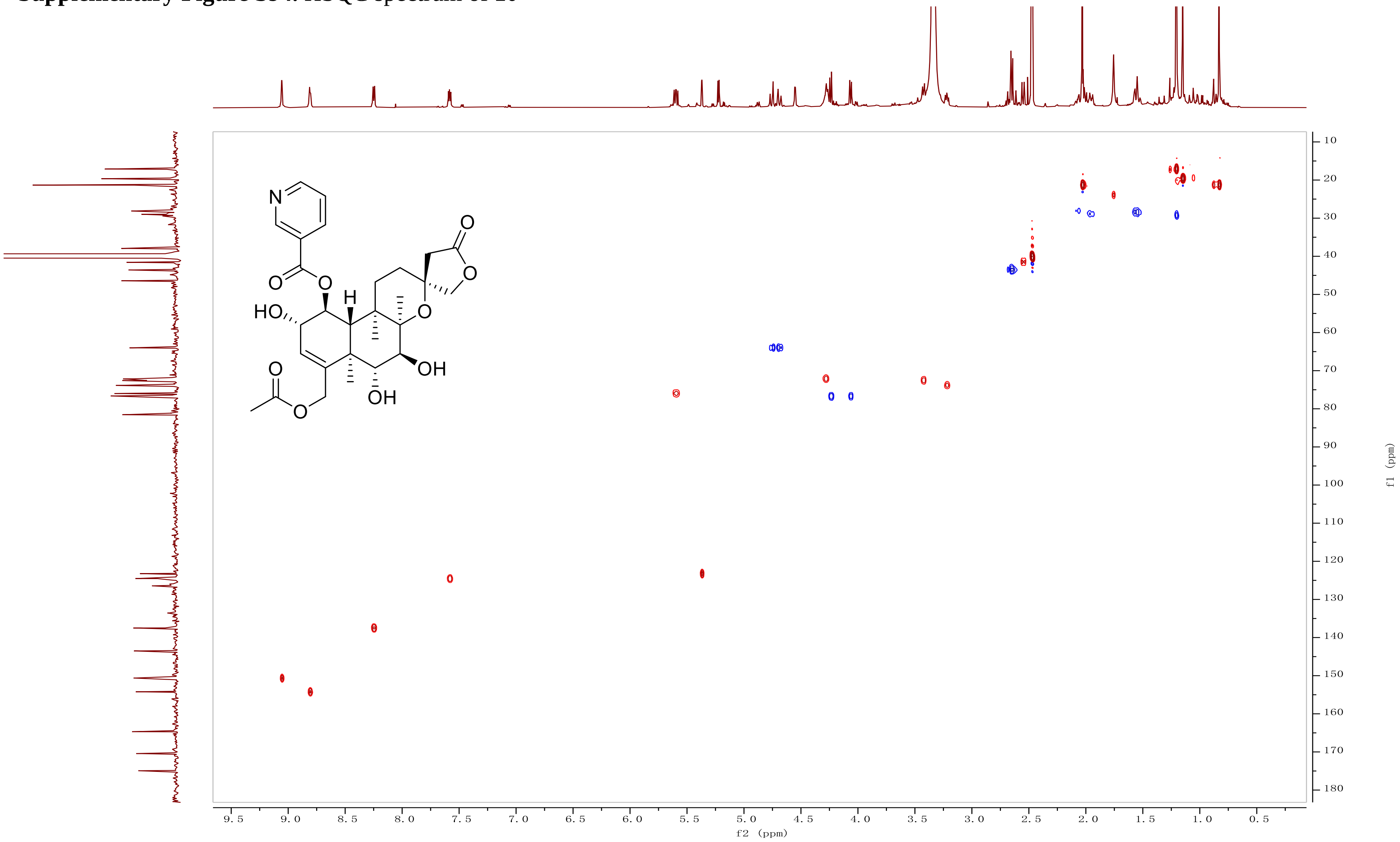

Supplementary Figure S95. HMBC spectrum of **10**

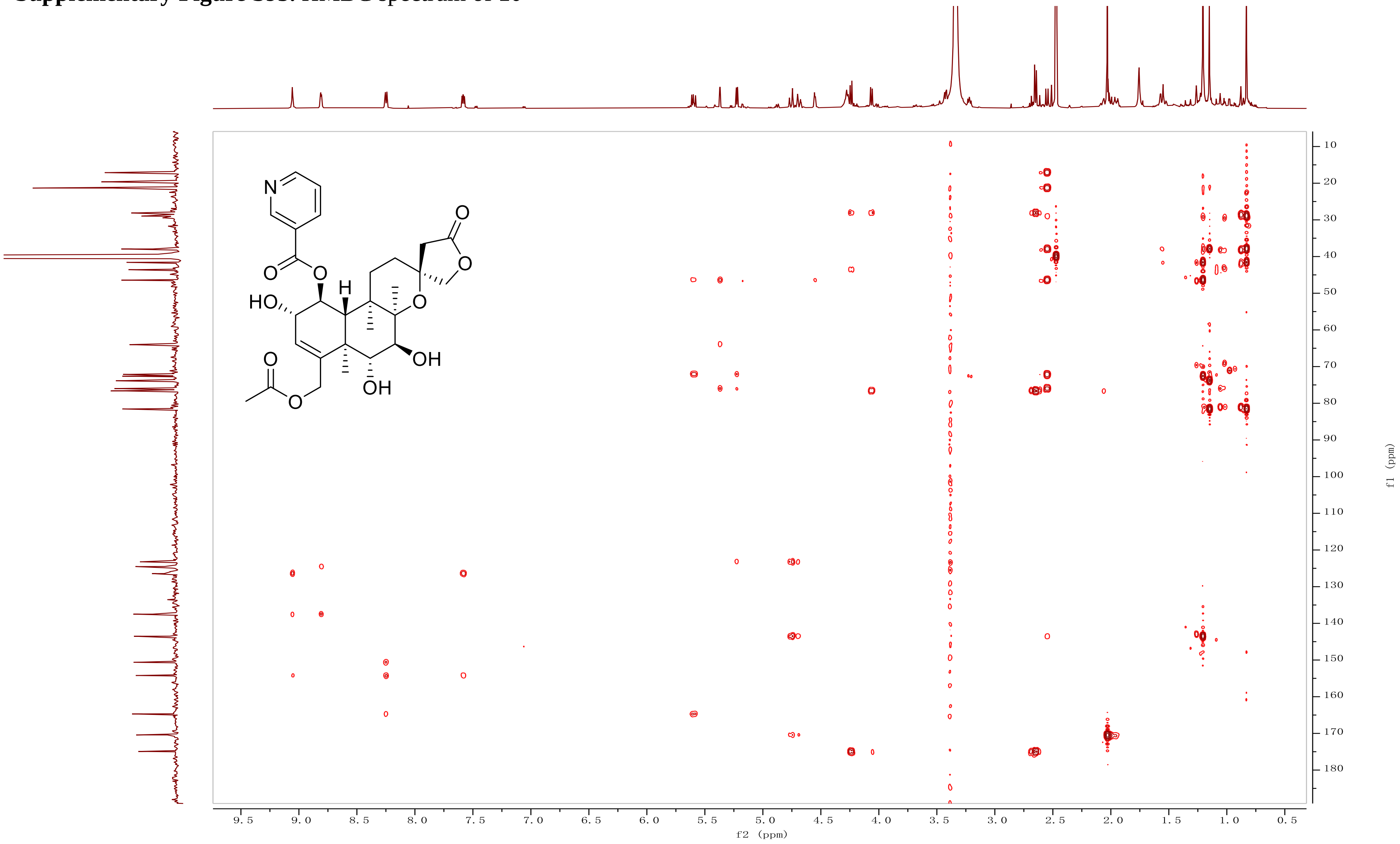

Supplementary Figure S96. NOESY spectrum of **10**

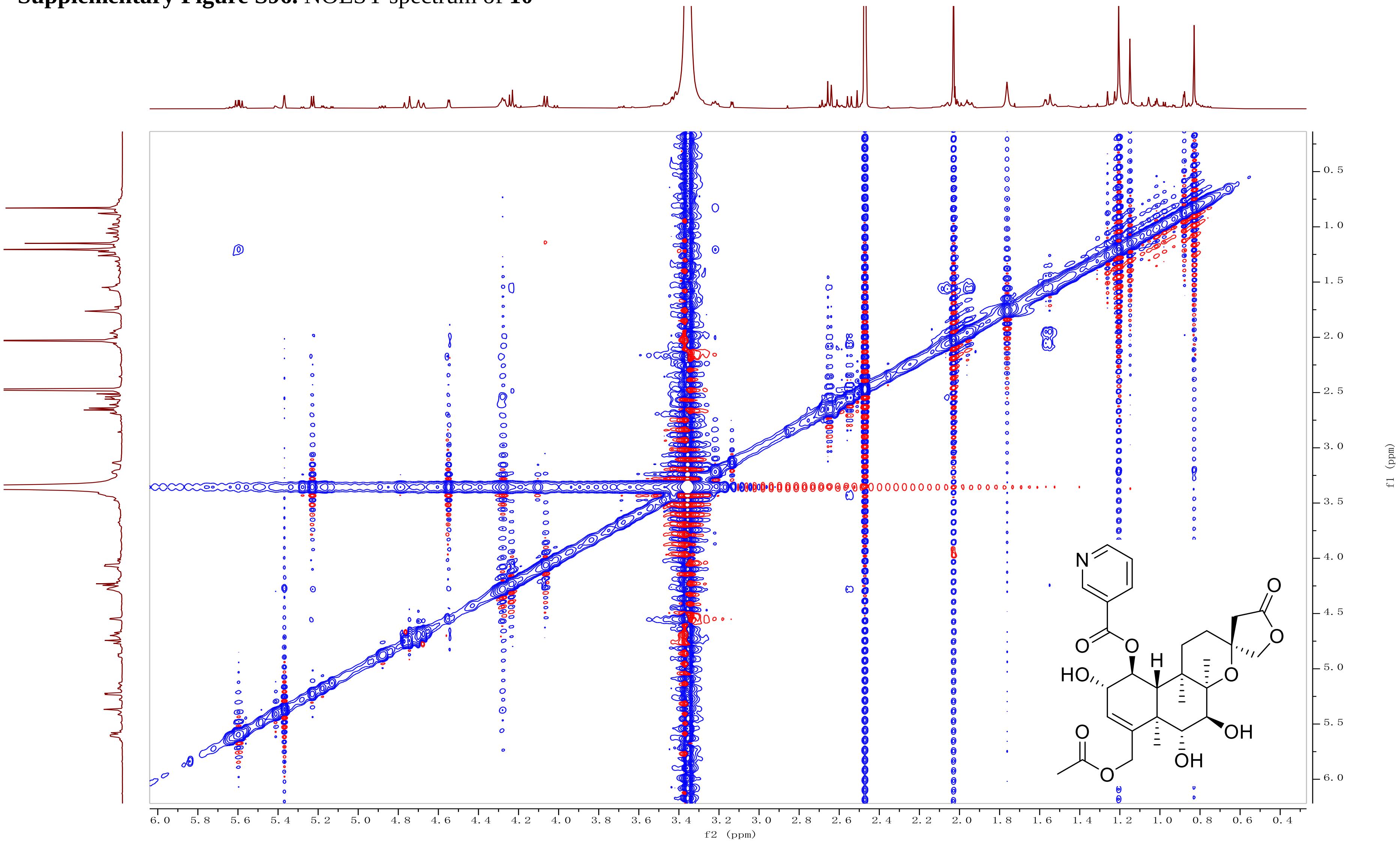

Supplementary Figure S97. HRESIMS spectrum of **10**

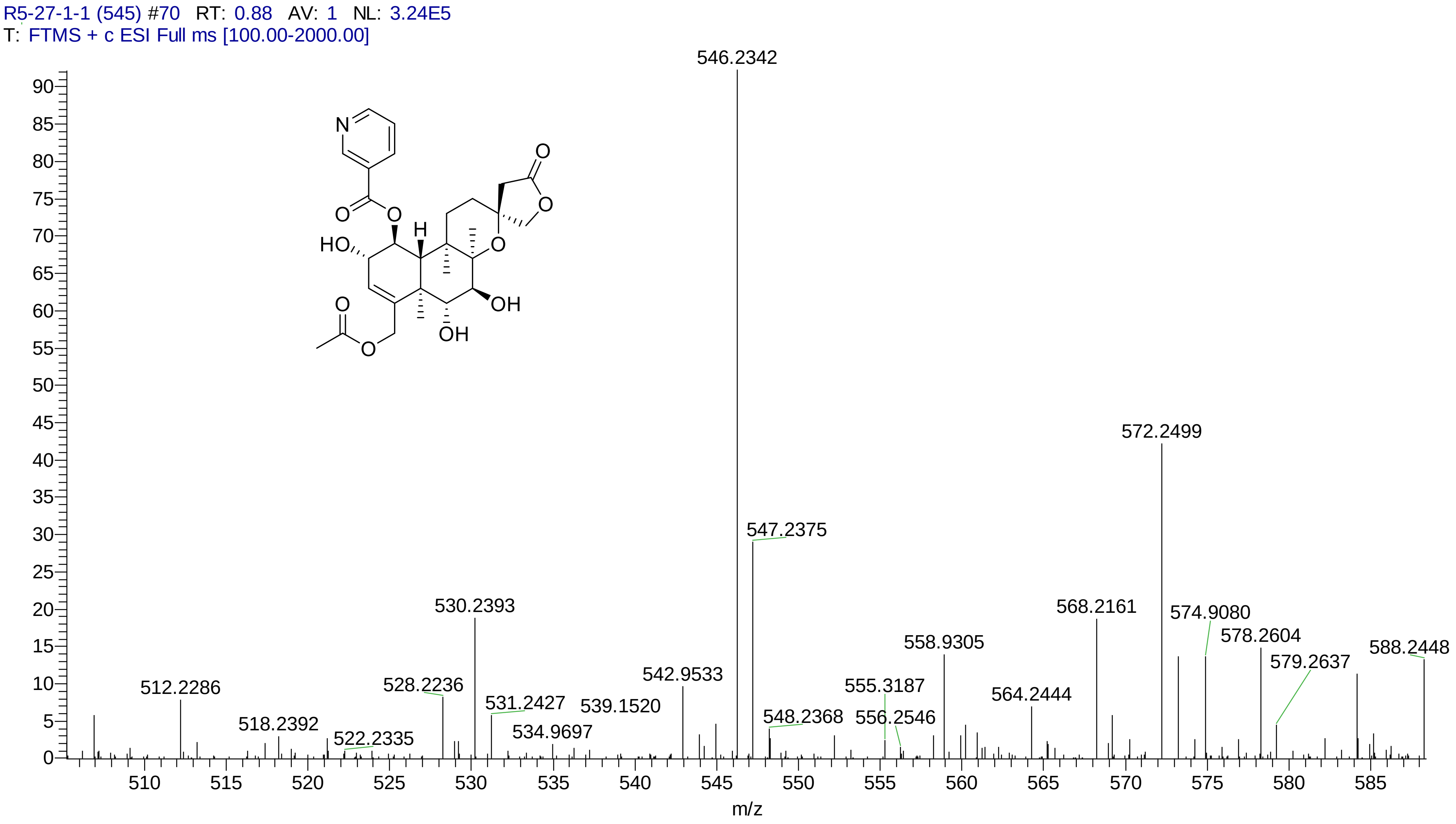

Supplementary Figure S98. IR spectrum of **10**

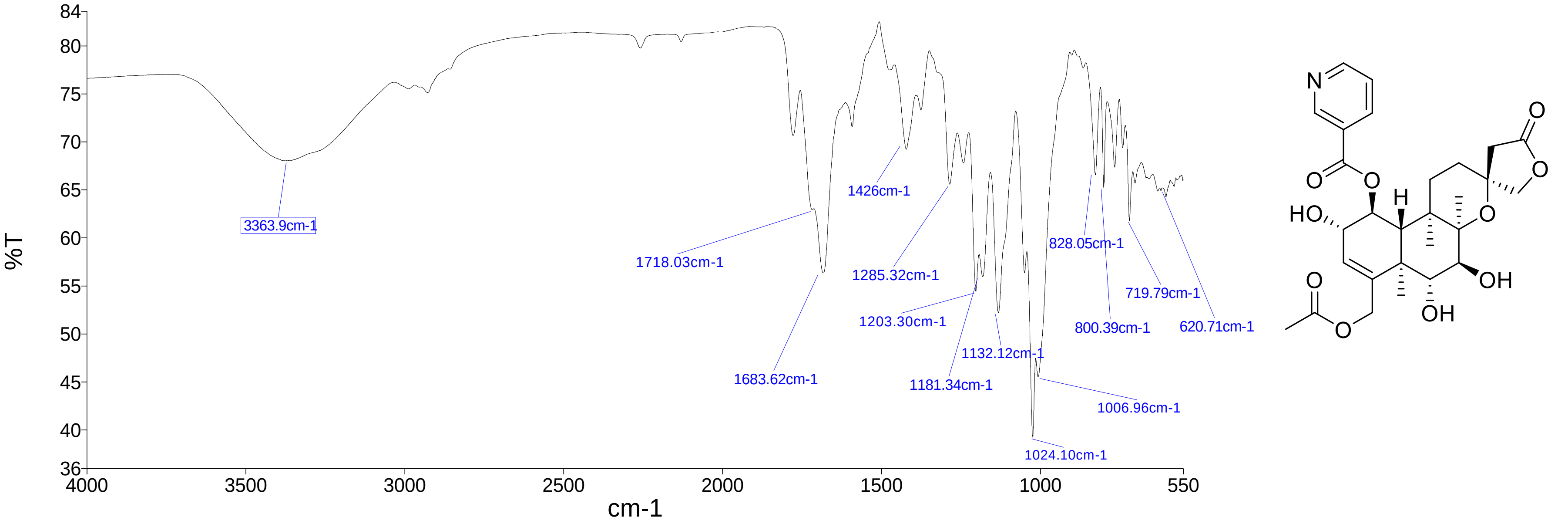

Supplementary Figure S99. UV spectrum of **10**

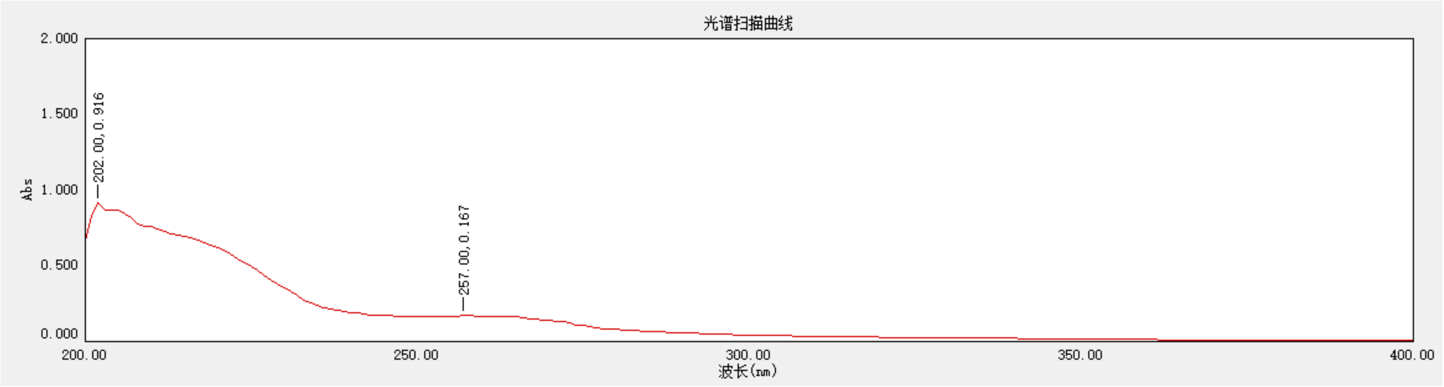

Supplementary Figure S100. CD spectrum of **10**

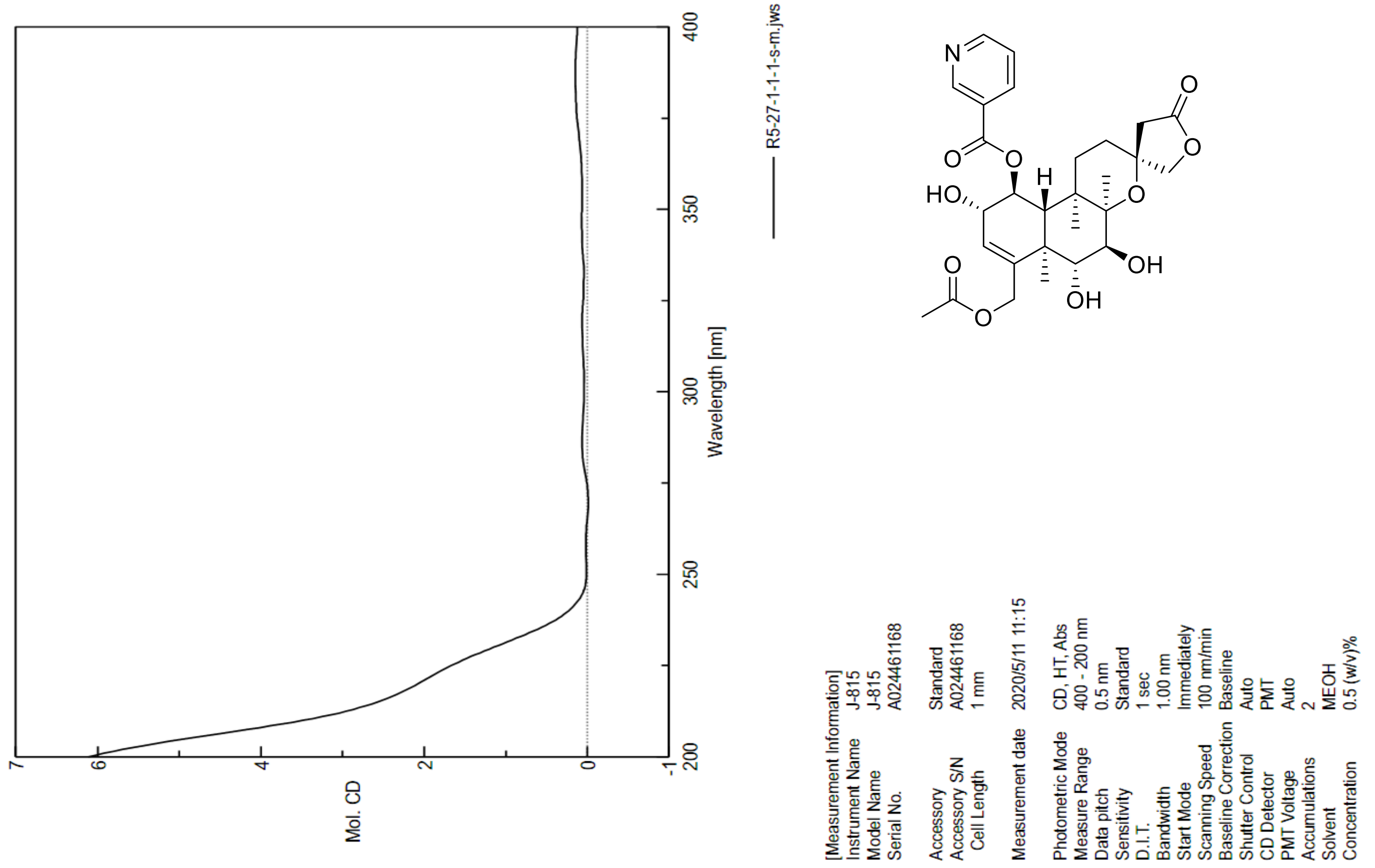

Supplement: Supplementary file 1 [file Data_Sheet_1.pdf]
